# Identification of the HECT E3 ligase UBR5 as a regulator of MYC degradation using a CRISPR/Cas9 screen

**DOI:** 10.1038/s41598-020-76960-z

**Published:** 2020-11-18

**Authors:** Lina Schukur, Tamara Zimmermann, Ole Niewoehner, Grainne Kerr, Scott Gleim, Beatrice Bauer-Probst, Britta Knapp, Giorgio G. Galli, Xiaoyou Liang, Angelica Mendiola, John Reece-Hoyes, Melivoia Rapti, Ines Barbosa, Markus Reschke, Thomas Radimerski, Claudio R. Thoma

**Affiliations:** 1 Novartis Institutes for Biomedical Research (NIBR) Oncology, Novartis, Basel, Switzerland; 2 NIBR Chemical Biology and Therapeutics, Novartis, Cambridge, USA; 3 Genomics Institute of the Novartis Research Foundation, LaJolla, USA; 4 Present Address: Basilea Pharmaceutica, Basel, Switzerland; 5 Present Address: Pharmaceutical Research and Early Development, Roche Innovation Center Basel, F. Hoffmann-La Roche Ltd, Basel, Switzerland

**Keywords:** Biological techniques, Cancer, Cell biology, Oncology

## Abstract

MYC oncoprotein is a multifunctional transcription factor that regulates the expression of a large number of genes involved in cellular growth, proliferation and metabolism. Altered MYC protein level lead to cellular transformation and tumorigenesis. MYC is deregulated in > 50% of human cancers, rendering it an attractive drug target. However, direct inhibition of this class of proteins using conventional small molecules is challenging due to their intrinsically disordered state. To discover novel posttranslational regulators of MYC protein stability and turnover, we established a genetic screen in mammalian cells by combining a fluorescent protein-based MYC abundance sensor, CRISPR/Cas9-based gene knockouts and next-generation sequencing. Our screen identifies UBR5, an E3 ligase of the HECT-type family, as a novel regulator of MYC degradation. Even in the presence of the well-described and functional MYC ligase, FBXW7, UBR5 depletion leads to accumulation of MYC in cells. We demonstrate interaction of UBR5 with MYC and reduced K48-linked ubiquitination of MYC upon loss of UBR5 in cells. Interestingly, in cancer cell lines with amplified MYC expression, depletion of UBR5 resulted in reduced cell survival, as a consequence of MYC stabilization. Finally, we show that MYC and UBR5 are co-amplified in more than 40% of cancer cells and that MYC copy number amplification correlates with enhanced transcriptional output of UBR5. This suggests that UBR5 acts as a buffer in MYC amplified settings and protects these cells from apoptosis.

## Introduction

Protein abundance is not only regulated by transcriptional and translational processes but also by the regulation of protein degradation, an important process that acts at the posttranslational level in order to maintain homeostasis^1^. Two major pathways mediate protein degradation in eukaryotes, the lysosomal proteolysis and the ubiquitin–proteasome pathway^2^. The half-lives of intracellular proteins can range between minutes to several days. Transcription factors (TFs) are amongst the most rapidly degraded proteins^3^. Their rapid turnover allows their level to adapt promptly to external stimuli, and as such enables a switch-like control mechanism of signaling nodes. TFs are involved in a large number of human diseases, including cancer, and are strongly associated cancer cell dependencies^4,5^.

A prominent example of such a TF is MYC, a very well described oncogene and a crucial driver of cancer cell growth. Supraphysiological abundance of MYC protein is known to mediate its oncogenic functions and is regulated at multiple levels, including MYC gene amplification, up-regulation of MYC promoter activities, regulation of MYC mRNA level as well as reduced MYC protein degradation by E3 ligases^6^. The E3 ligase tumor suppressor protein F box protein 7 (FBXW7) has been described as a key regulator of MYC protein turnover in cells by recognizing MYC phosphorylation sites at Thr58 and Ser62 and mediating MYC ubiquitination for subsequent proteasomal degradation^7,8^.

Covalent ligation of ubiquitin, a 76-amino acid polypeptide, to substrate proteins serves as key recognition signal for rapid degradation by the proteasome complex and is mediated by a highly regulated ATP-dependent enzymatic cascade^2,9–11^. This cascade begins with ubiquitin activation through the E1 ubiquitin activating enzyme, followed by ubiquitin conjugation by an E2, and finally the attachment of ubiquitin to lysine residues by a diverse group of substrate-specific E3 ligases^12^. E3 ligases are grouped into two main families: RING ligases that include RING and RING-like ligases and their accessory proteins (encoded by about 600 genes) and the HECT E3 ligases (around 30 in the human genome)^13^. A key difference between the RING and HECT type family is that the RING domain serves as an E2 recruitment domain to optimally position the ubiquitin-charged E2 for direct transfer of the ubiquitin to the substrate. Whereas HECT-type ligases do have a catalytic cysteine, that first accepts the ubiquitin via thioester bond formation from an ubiquitin-charged E2 for subsequent direct transfer to a substrate.

An imbalance of this tightly regulated protein degradation process is observed in many types of cancers, causing cellular malfunctions and disease development^14^.

MYC controls a wide spectrum of cellular functions, such as cell cycle, differentiation, survival, transcription, translation, immune surveillance, and metabolism^6^. In healthy cells, MYC increase has no effect or can be detrimental to the cell by inducing proliferation arrest, apoptosis or senescence. In contrast, in cancer cells, increased MYC activity was shown to cooperate with other oncogenic events to drive malignant transformation, for example by overcoming senescence (e.g. through overexpression of BCL-2, loss of p53 or loss pf 19ARF)^15–17^. Furthermore, MYC level can significantly influence the outcome of its activation on the cell fate. Low increase in the level of MYC seems to be linked to cellular proliferation, while robust activation of MYC appears to be more essential to DNA damage and apoptosis through the activation of the ARF/p53 tumor surveillance pathways^16,18^.

Although multiple studies have focused on identifying the transcriptional regulation network that drives MYC expression in cancer, little efforts are invested into studying the posttranslational regulation. In this study, we aimed at developing a screening tool to identify components of the ubiquitin proteasome system (UPS) pathway that drive the degradation of proteins of interest. In particular, we focused on MYC as a prototype for undruggable transcription factors.

To focus on genes that potentially directly influence MYC protein level, we developed a CRISPR/Cas9-based screen combined with fluorescent MYC sensor in order to discover modifiers of MYC stability. We could identify FBXW7, a known MYC E3 ligase, confirming the validity of the screen. In addition to this known regulator, we discovered the HECT E3 ligase UBR5 as a direct regulator of MYC protein abundance. We validated these findings in various cell lines, and could show that depletion of UBR5 reduces cellular viability in cell lines with high MYC expression level through the increase of MYC protein level. This observation further confirms previous studies indicating that high level of MYC protein can drive the cell into apoptosis^16,19^. Finally, we show that in cancer cell lines there is a strong correlation between MYC and UBR5 amplification, suggesting a selection pressure for co-amplification and a potential vulnerability in this subpopulation towards loss of function of UBR5.

## Results and discussion

### Combination of fluorescence-based sensors and CRISPR/Cas9 technology for the identification of protein stability modulators in mammalian cells

**Figure 1:**
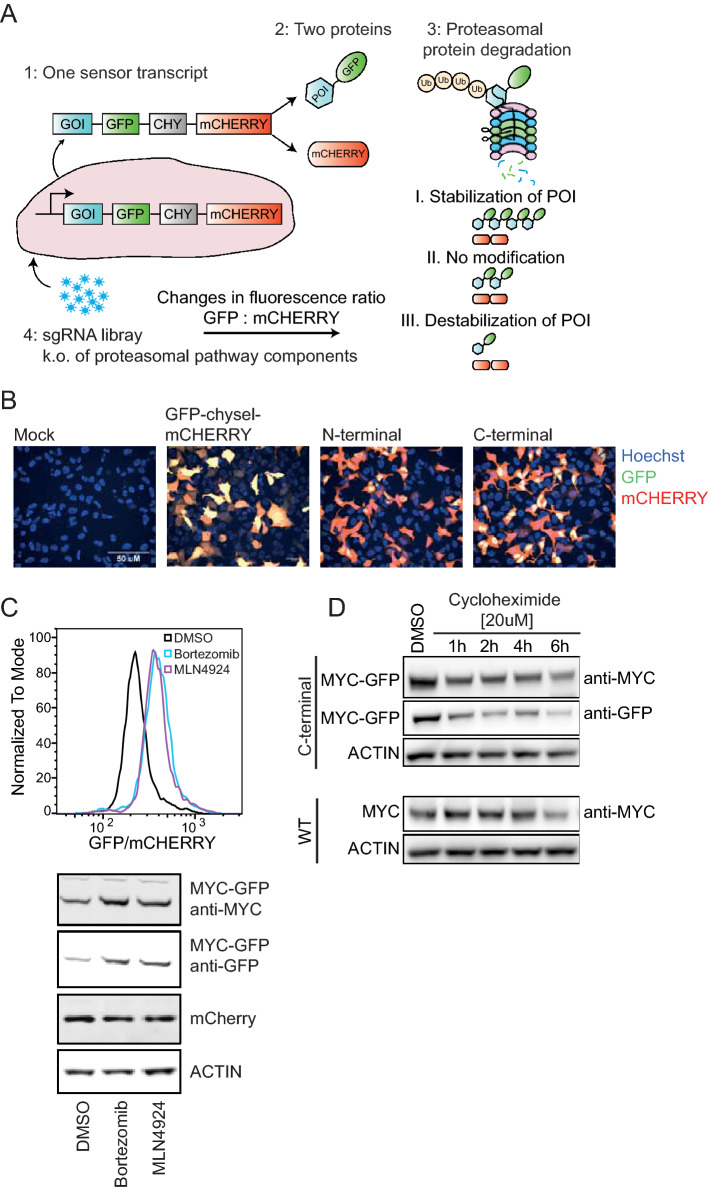
Design and assembly of the UPS screen. (**A**) The UPS gene cassette drives the expression of a single transcript containing the GFP-fusion to the gene of interest (GOI) and mCHERRY, separated by the chysel peptide (CHY). The sgRNA library targeting the proteasomal pathway components is introduced into mammalian cells that stably express the UPS cassette and Cas9 through lentiviral infection. Stability modulation through sgRNA-mediated knockout is represented by changes in GFP/mCHERRY signal ratio and FACS-sorted into separate bins, followed by NGS sequencing. (**B**) Confocal microscopy of HEK293A cells expressing either Mock DNA, GFP-chysel-mCHERRY, or N- or C-terminal GFP-CHYSEL-mCHERRY tag to MYC ORF. (**C**) Single cell clone B6 was treated with MLN4942 ([1 µM]) and Bortezomib ([50 nM]) for 24 h and GFP/mCHERRY ratio was compared to DMSO control using FACS based analysis as well as western blot. The Y-axis scaling (Normalized To Mode) is a feature in the FACS analysis program flowjo based on normalizing to the peak height at mode of the distribution (the maximum Y-axis value in the absolute count = 100%).(**D**) Cycloheximide chase assay to compare the half-life of wild type MYC and MYC-GFP-chysel-mCHERRY in HEK293A.

The UPS screening tool is a cell-based method that combines CIRSPR/Cas9-mediated genetic modification with fluorescence-based readouts^20,21^ to allow the discovery of protein stability modulators (Fig. 1A). We constructed a fluorescent sensor, embedded into a bicistronic viral vector, which drives the expression of a single transcript encoding two proteins: (1) a GFP fusion protein (here MYC-GFP) and (2) mCHERRY, separated by the self-cleaving chysel (CHY) peptide sequence^22^. The same transcript serves as a baseline signal for the overall expression of the bicistronic gene cassette. Changes in MYC protein level were monitored by measuring GFP versus mCHERRY ratios using FACS-based readouts. Upon ubiquitination and subsequent degradation of MYC-GFP, the GFP signal is reduced in comparison to mCHERRY. In contrast, MYC stabilization can be measured by the increase in GFP signal relative to mCHERRY. This principle is applied in combination with CRISPR/Cas9-based knockout, induced by a sgRNA library targeting components of the proteasome system pathway, such as E3 ligases or deubiquitinases. Depending on the changes in GFP to mCHERRY ratio (GFP/mCHERRY), stabilization or destabilization of MYC is linked to the respective gene knockout using next generation sequencing analysis^23^.

For the identification of MYC stability modulators, we used mammalian HEK293A cells that harbor the above-described fluorescence-based MYC sensor, which was fused either at the N- or C-terminus of the MYC coding sequence (Fig. 1B). We used confocal microscopy to assess the intracellular localization of GFP. In line with previous studies^24^, visual inspection of transiently transfected cells (determined by mCHERRY expression) confirmed nuclear localization of MYC (reflected by the GFP signal) with the C-terminal tag, while N-terminal GFP-MYC tagging reduced nuclear localization (Fig. 1B). Consequently, we engineered HEK293A cells that stably express the MYC-GFP-chysel-mCHERRY sensor and Cas9 protein and generated single cell clones derived from the stable MYC-sensor and Cas9 expressing cells (Supplementary Fig. 1). In order to define criteria for choosing a single cell clone with the optimal property for the UPS screen, we utilized MLN4924, which inhibits protein neddylation, a reaction catalyzed by the NEDD8-activating enzyme 1 (NAE1) and required for the activation of the cullin ring ligase (CRL)-mediated ubiquitination^25,26^. Previous studies showed that MYC degradation is regulated by FBXW7^7,8^, an E3 ligase receptor of the CRL1 complex. Inhibition of NAE1 through MLN4924 would therefore lead to reduced MYC degradation by FBXW7. Indeed, we observed increased GFP/mCHERRY in the presence of MLN4942, with the highest level reached in the single cell clone B6 (Supplementary Fig. 1A). Interestingly, in MYC-GFP sensor-expressing B6 clone as well as two other clones (clone A1 and A5), wild type MYC protein expression was suppressed in comparison to parental HEK293A cells (Supplementary Fig. 1B). This observed reduction in the wild type MYC protein level could be due to negative autoregulation of endogenous MYC expression^27^ through the overexpression of MYC-GFP in those cells. Using flow cytometry, we assessed GFP and mCHERRY expression levels in the B6 clone (Supplementary Fig. 1C) as well as Cas9 functionality by transfecting sgRNAs that target GFP or mCHERRY (Supplementary Fig. 1D), which resulted in the decrease in GFP or mCHERRY signal in comparison to a non-targeting (NT) sgRNA, respectively, indicating the presence of functional Cas9 protein. We further characterized the MYC-GFP single cell clone B6 by treatment with the proteasomal inhibitor Bortezomib and MLN4924 and measured MYC-GFP stabilization. A shift in the GFP/mCHERRY ratio observed by flow cytometry due to enhanced levels of MYC-GFP without a change in mCHERRY levels, as confirmed by western blot suggests that those cells can be used as a cellular model to identify proteasome-mediated MYC regulation (Fig. 1C).

Finally, we used cycloheximide, a translational inhibitor, to uncouple MYC degradation from expression, allowing us to compare the half-lives of MYC-GFP fusion protein to wild type MYC in B6 sensor cells and HEK293A wild type cells, respectively. The results shown in Fig. 1D indicate no significant changes in the protein half-life through the fusion of GFP to MYC protein compared to endogenous MYC, both proteins show a similar half-life of approximately 1–2 h. We concluded that the C-terminal MYC tag in mammalian HEK293A can serve as an adequate model for the identification of MYC stability modulators.

### UPS screening setup and profiling of MYC protein regulation by proteasomal components

**Figure 2:**
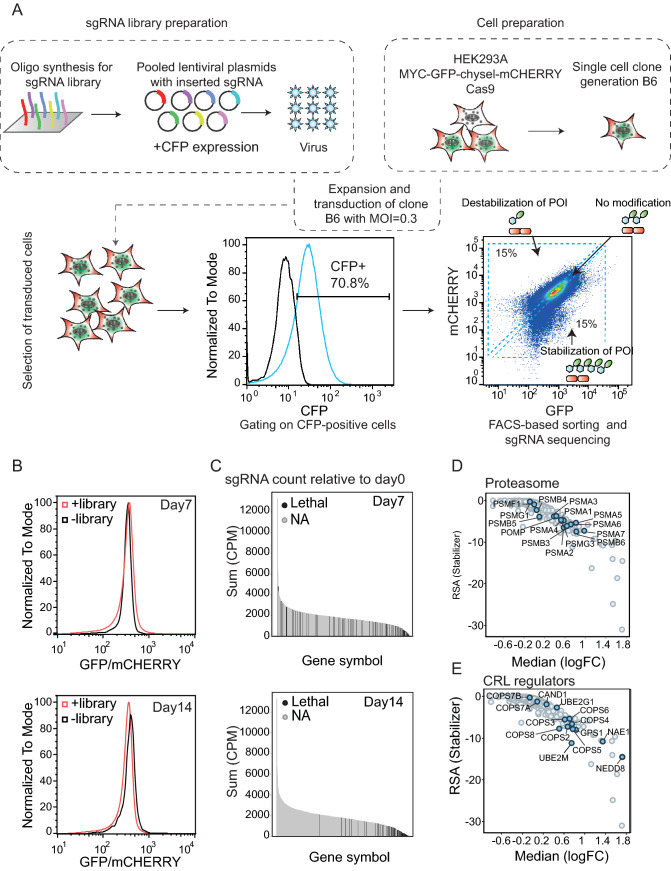
UPS screen workflow and selection of MYC stability modulators. (**A, B**) HEK293A cells are engineered to stably overexpress C-terminal MYC sensor and Cas9. A single cell clone (B6) is generated from a multi-clonal cell population, expanded and infected with the CFP-labeled sgRNA encoding lentiviral library at a multiplicity of infection (MOI) of 0.3. After seven or 14 days of infection, puromycin-selected and CFP-positive cells (+ library) were FACS-sorted based on their GFP/mCHERRY ratio (high, low, or unmodified) and compared to uninfected cells (− library). Enrichment of sgRNA in the respective bins is compared to unsorted cell population. (**C**) sgRNA count on day 7 and day 14 relative to day 0 in cells with high GFP/mCHERRY ratio comparing genes essential for cell survival (lethal) to nonessential genes (NA). (**D, E**) Plots showing gene enrichment score in high GFP/mCHERRY-sorted cells relative to unsorted cell population visualized as LogFC.

We next performed a UPS screen, using a focused pooled library covering the ubiquitin proteasome system involved in protein degradation pathways. The target gene content included following gene families: E1-, E2-, and E3 ligases and regulators, proteasomal genes, deubiquitinases as well as multiple control genes and direct screen controls, such as GFP and mCHERRY. For each gene, at least 10 different sgRNAs were designed and synthesized as described previously^28^ as oligos on a chip for subsequent cloning as a pool into a lentiviral expression vector co-expressing CFP as a control (Fig. 2A). Lentivirus produced from this library was then used to infect a HEK293A single cell clone (B6) expressing the MYC-GFP/mCHERRY stability sensor using a MOI of 0.3. Library-infected cells were selected with puromycin until CFP-positive cells were sorted into three bins based on the GFP/mCHERRY signal ratio. In total, a representation of 1000 cells were collected per sgRNA present in the library. The first bin contained 15% of total cell population with low GFP/mCHERRY, representing cells infected with sgRNAs that reduce MYC-GFP protein level; the second bin with 15% of total cell population with high GFP/mCHERRY representing cells infected with sgRNAs that enhance MYC-GFP protein level. Lastly, the third bin collecting infected cells with an unmodified GFP/mCHERRY ratio that express sgRNAs without effect on the modulation of GFP/mCHERRY (Fig. 2A,B).

From sorted cells, the integrated sgRNA was amplified and analyzed using next generation sequencing (Fig. 2A,B). When comparing GFP/mCHERRY signal in sgRNA library-infected versus uninfected cells on the whole population level on day 7 or day 14 post-infection, we found that the GFP/mCHERRY modulation (increase or decrease) was only observed on day 7, while no changes were observed on day 14 (Fig. 2B). This indicates that modulation of MYC is time-dependent, and the most significant changes are seen at earlier time points. However, it does not exclude significant enrichment of very few sgRNAs at later time points. In order to further understand the role of the sorting time point on the screen outcome, we assessed the sgRNA count for each gene on the respective day (day 7 or day 14) and compared the data to the sgRNA count on day 0 (time point of the infection) (Fig. 2C). For this analysis, we refer to the previously published project DRIVE, in which the viability effects of shRNA-mediated knockdown for 7837 genes in 398 cancer cell lines were assessed^29^ and use the classification as published in this study for lethal genes in our sub-library. In DRIVE, a gene was classified as “lethal” if the shRNA construct of that particular gene was depleted in most of the screened cell lines, indicating that the respective gene is essential for cell viability. For example, proteasomal subunits are essential for cell viability and therefore have been shown to be depleted over time compared to other non-essential genes. In line with this study, our analysis shows a strong reduction of the sgRNA count of such lethal genes on day 14, the same time point that was used for project DRIVE, whereas those sgRNAs could still be detected and are not depleted on day 7. These results suggest that the lack of enrichment of sgRNAs targeting lethal genes at day 14 is caused by reduced cell fitness. Thus, we concluded that later time points are more suitable for screens aiming at identifying lethality, as those screens rely on the detection of sgRNA depletion^29^. In contrast, for sgRNA enrichment analysis, shorter time points are more suitable, e.g. to identify protein stability modulation^30^. This may explain why we did not observe signal enrichment in low and high in GFP/mCHERRY bins on day 14 (Fig. 2B). Therefore, we performed all our analyses screening data obtained on day 7 after infection.

The lentiviral library contained sgRNAs targeting MYC, GFP and mCHERRY as internal controls. As predicted, we found enrichment of cells with sgmCHERRY in the high GFP/mCHERRY population; however, sgMYC and sgGFP were not identified in the low GFP/mCHERRY population (Supplementary Fig. 2A, B). The reason for the latter observation is that the mCHERRY coding sequence is downstream of MYC and GFP in the C-terminal MYC sensor cassette and the knockout of either MYC or GFP would result in a frame-shift in the downstream DNA sequence. We further validated this by single knockout of GFP or mCHERRY over time (Supplementary Fig. 2C, D) and found that the knockout of GFP reduced the GFP and mCHERRY level in comparison to sgNT, whereas targeting mCHERRY only resulted in the reduction of the mCHERRY signal. We did observe a slight decrease in the GFP signal upon transfection with sgmCHERRY and we hypothesize that the latter signal decrease occurred due to the high DNA sequence homology of GFP and mCHERRY, which may have resulted in reduced specificity of the sgmCHERRY sequence.

In accordance with our data that proteasome and NAE1 inhibition (Fig. 1C and Supplementary Fig. 1A) lead to enhanced MYC-GFP level, we confirmed that knockout of the majority of proteasomal subunits (Fig. 2D) as well CRL regulators (Fig. 2E) are enriched in the high GFP/mCHERRY population. The latter analysis included NAE1, UBE2M and NEDD8 as positive CRL regulators via NEDD8 neddylation of cullins^31^. On the other hand, we also identified negative CRL regulators, such as COP9 signalosome subunits that act by removing the NEDD8 mark and hence deactivate the CRL complex. It has been previously shown that the CRL complex could only maintain functionality when also the negative regulators are present, suggesting that removal of NEDD8 is as crucial to CRL activity as it’s attachment^32^. A recent study using sgRNA-mediated knockout has shown that the loss of the COP9 signalosome as well as CAND1, UBE2G1 impaired lenalidomide-dependent CRL4-CRBN functionality^33^. Aligning with this study, we show that the knockout of those above-mentioned regulators also lead to stabilization (impaired degradation) of MYC-GFP. In summary, we discovered key regulators as hits in our screen on day 7 covering multiple levels of the UPS pathway from early upstream regulators such as E1 (NAE1) via E2 (UBE2M and UBE2G1) towards more downstream regulators such as Cullin E3 ligase regulators (COPS) and the most downstream proteasome subunits. In addition, we found also one deubiquitinating enzyme (DUB) USP22, previously described in literature to mediate MYC stability in breast cancer cell lines (Supplementary Fig. 3A)^34^. In contrast, we could not validate USP28, a well-described DUB for MYC^35^. Discovering genes that further destabilize MYC and reduce its half-life might be challenging in this system where MYC-GFP is turned over rapidly (see Fig. 1D) as well as we cannot exclude cell-type specific dependencies towards MYC protein stability regulation. A complete list of the genes included in the library with the respective LogFC and RSA values for cells sorted into the low and high GFP/mCHERRY bins on day 7 and day 14 is summarized in Supplementary table 1.

**Figure 3:**
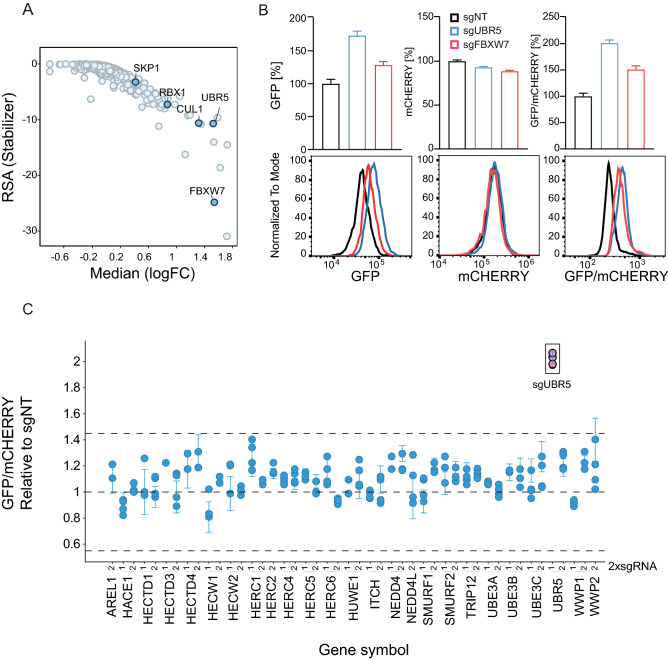
UBR5 is a modulator of MYC stability. (**A**) Gene enrichment score in high GFP/mCHERRY-sorted cells relative to unsorted cell population on day 7 of library infection visualized as LogFC. (**B**) sgRNA-mediated knockout of UBR5 (sgUBR5), FBXW7 (sgFBXW7) or a non-targeting sgRNA control (sgNT) in B6 clone and analysis of GFP, mCHERRY, and GFP/mCHERRY ratio using FACS 7 days post-knockout. (**C**) MYC degradation screen using an arrayed sgRNA sublibrary. MYC-GFP/mCHERRY cells were infected with lentiviruses encoding for sgRNAs targeting HECT E3 ligases. 2 sgRNAs were used per gene (indicated as 1, 2). Seven days after infection and selection with puyromycin, GFP/mCHERRY was measured using flow cytometry and the increase in GFP/mCHERRY ratio upon knockout of the singlegenes was compared to a non-targeting control (sgNT).

In summary, our data highlight the robustness of our screening system to discover novel MYC stability mediators, especially genes whose knockout resulted in enhanced MYC-GFP signal at early time points.

### Discovery of the HECT-type E3 ligase UBR5 as a MYC protein regulator

Beyond the above mentioned generic UPS pathway regulators and components, we also found the whole F-box and WD repeat domain containing protein 7 (FBXW7) CRL complex, including CUL1, SKP1, RBX1 and the MYC substrate receptor FBXW7^7,8^ (Fig. 3A). FBXW7 is the best described and validated E3 ligase F-box receptor of MYC. In addition, two other F-box proteins FBXO32^36^ and FBXL14^37^ have also been proposed as MYC regulators but could not be validated in our screen (Supplementary Fig. 3A). We cannot rule out either compensatory regulation via FBXW7, or cell type specific MYC protein stability regulation. However, even in the presence of strong FBXW7-mediated MYC degradation in our cellular screening system, which could compensate for other potential ligases, we discovered another E3 ligase that has a strong effect on MYC protein turnover: UBR5 (Fig. 3A). UBR5 is a HECT-type ligase also known as EDD, EDD1, HHYD, KIAA0896 or DD5^38^. Our data demonstrate that the screening approach faithfully recapitulate known pathways involved in MYC post-translational stability regulation (e.g. FBXW7) and further identifies UBR5 as a novel MYC E3 ligase. During manuscript preparation, Qiao et al. published their findings of UBR5 as a novel regulator of MYC stability using an orthogonal siRNA approach in a U2OS MYC-T58A mutant model^19^, thereby both independent studies increase the confidence in the role of UBR5 as a regulator of MYC protein stability.

In order to validate our screening results, we performed a UBR5 knockout in the B6 clone and compared the increase in the GFP/mCHERRY ratio to cells with FBXW7 knockout. Increase in GFP as well as GFP/mCHERRY was observed to a higher extent in UBR5 knockout than in the FBXW7 knockout cells (Fig. 3B). As expected, mCHERRY level remained unmodified, indicating that similar to FBXW7, UBR5 plays a role in post-translational regulation of MYC-GFP rather than on the transcriptional level. To follow up the role of HECT-type ligases and in particular UBR5 as a novel regulator of MYC, we assembled a small orthogonal sub-library focused on HECT E3-ligases (2 different sgRNAs per gene) to perform an arrayed screen in the same MYC-GFP-mCHERRY cell line (B6 clone). We could confirm UBR5 as the only HECT ligase that leads to a strong MYC-GFP stability modulation 7 days post lentiviral transduction with individual sgRNAs (Fig. 3C).

**Figure 4:**
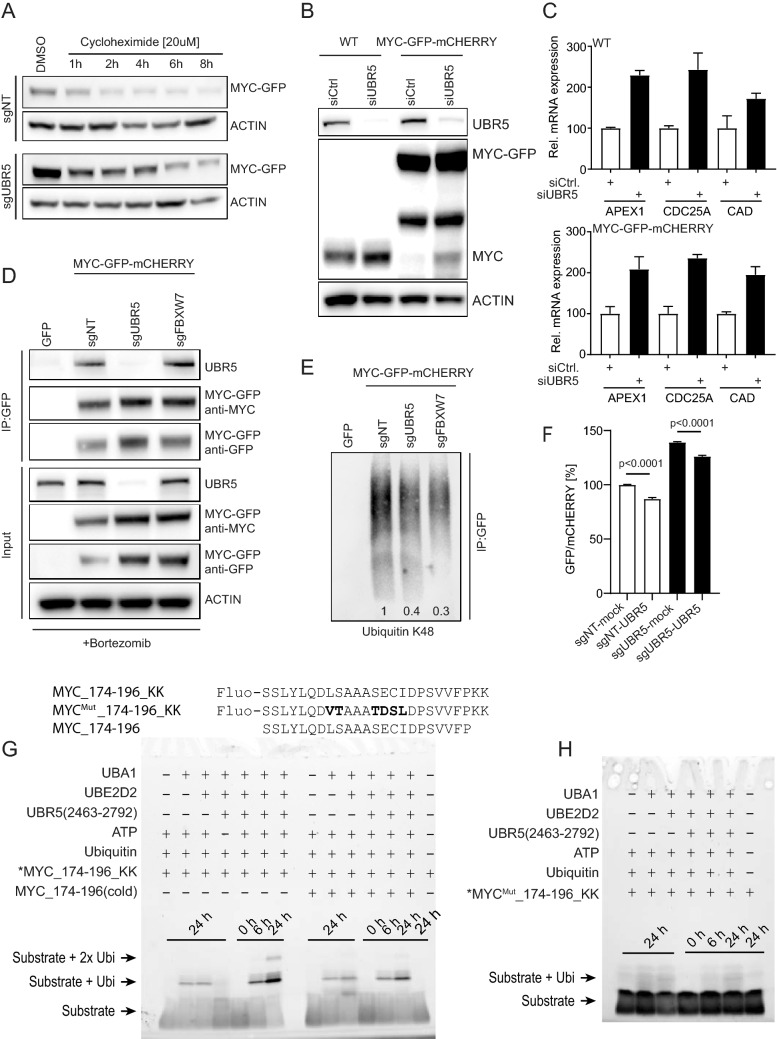
Validation of UBR5 as a regulator of MYC protein stability and interaction. (**A**) B6 clone was transfected with sgNT or sgUBR5 for 7 days, followed by treatment with cycloheximide at 20 µM concentrations. MYC-GFP level were assessed by western blot at the indicated time points (quantification is shown in Supplementary Fig. 4A). (**B**) Increase in the protein level of MYC-wt as well as MYC-GFP fusion protein upon UBR5 knockdown in HEK293A cells (WT) and B6 clone (MYC-GFP-mCHERRY). (**C**) qPCR analysis of MYC target genes upon knockdown of UBR5. (**D**) Immunoprecipitation analysis showing interaction between UBR5 and MYC in B6 clone. (**E**) Decrease in ubiquitination of MYC-GFP immunoprecipitated protein upon knockout of UBR5 and FBXW7. Relative decrease in K48 ubiquitination was normalized to sgNT (1) and values are indicated on the bottom of the corresponding lane. (**F**) B6 clone was transfected with sgNT or sgUBR5 for 4 days and then further transfected with vector expressing mock DNA or UBR5 ORF, respectively to rescue the phenotype. GFP/mCHERRY was measured 72 h after transfection with rescue construct. (**G**) Ubiquitination assay utilizing UBR5(2463–2792) and peptide substrates MYC_174–196_KK (Fluo-SSLYLQDLSAAASECIDPSVVFPKK) and MYC_174–196 (SSLYLQDLSAAASECIDPSVVFP) resolved on a 5–20% SDS-PAGE gel. Fluorescein fluorophore was detected on a ChemiDoc System (Bio-Rad). (**H**) Ubiquitination assay utilizing UBR5(2463–2792) and the conservatively mutated peptide substrate MYC_174–196_KK^Mut^ (Fluo-SSLYLQD**VT**AAA**TDSL**DPSVVFPKK), in which mutated residues are highlighted in bold. Samples were resolved on a 5–20% SDS-PAGE gel and fluorescence was detected on a ChemiDoc System (Bio-Rad).

In line with the MYC stabilization observed after knockout of UBR5, we measure an increase in the half-life of the MYC-GFP fusion protein upon depletion of UBR5 (sgUBR5) using a cycloheximide (CHX) chase assay in comparison to the non-targeting control (sgNT) (Fig. 4A and Supplementary Fig. 4A). Western blot quantification as well as flow cytometry analysis revealed significant MYC protein stabilization upon UBR5 knockout, particularly during early time points of CHX treatment. These findings are in agreement with a previously published study^19^.

To further validate our findings, we used siRNA-mediated knockdown of UBR5. We found that not only MYC-GFP fusion increased upon knockdown of UBR5 in B6 cells but also endogenous wild type MYC (MYC-wt) in HEK293A, confirming that UBR5-mediated MYC regulation is not affected by the GFP-tag (Fig. 4B). Elevation of established MYC target genes (APEX1, CDC25A, and CAD) as a consequence of siUBR5 treatment in both MYC-wt and MYC-GFP-mCHERRY cells further supports this finding (Fig. 4C). In order to gain a mechanistic understanding on UBR5-dependent MYC regulation, we assessed binding of UBR5 to MYC and performed pulldown experiments on MYC-GFP fusion protein in B6 cells using GFP-trap beads. As a control, we used sgRNA-mediated knockout of UBR5 or FBXW7 and compared the data to a non-targeting control or cells expressing GFP only as a negative control (Fig. 4D). Our data show that UBR5 is specifically co-immunoprecipitated with the MYC-GFP fusion protein independent of FBXW7, knockout of UBR5 confirms specificity of the immunopreciptation blot (Fig. 4D). To understand if the interaction between UBR5 and MYC has also a functional role, we measured K48-linked polyubiquitation and observed reduced level on precipitated MYC-GFP after knockout of UBR5 comparable to the reduction observed in cells lacking FBXW7 (Fig. 4E). In a next step, we transfected the B6 clone with sgNT or sgUBR5 for 72 h, followed by transfection of an UBR5-expressing vector. Using flow cytometry measurements, we could show that the overexpression of UBR5 in cells with or without UBR5 knockout reduced the GFP/mCHERRY ratio significantly (Fig. 4F).

Having shown cellular evidence for UBR5-mediated MYC ubiquitination, we sought to investigate if this is mediated through direct interaction using a biochemical in vitro ubiquitination approach. Qiao et al.^19^ used cellular immunoprecipitation assays with MYC deletion constructs to propose amino acids 181–189 of MYC as a potential candidate for UBR5 interaction. Here, we show ubiquitination catalyzed by a C-terminal UBR5 construct spanning amino acids 2463–2792 (including the HECT domain, referred to as UBR5 (2463–2792)) onto two lysines we attached to the C-terminus of a MYC peptide spanning amino acids 174–196 (Fig. 4G, *MYC_174–196_KK). Addition of UBR5 (2463–2792) to the fully reconstituted E1–E2 cascade only in the presence of ATP shows ubiquitination after 6 and 24 h. Mono-ubiquitinated MYC_174–196_KK substrate is > 2.5 fold enhanced after 24 h and in addition, we observe bi-ubiquitinated substrate compared to samples without UBR5 (2463–2792) (Fig. 4G, left gel). Competition of this reaction using the same non-labeled “cold” peptide reduces the ubiquitinated species approximately twofold at both time points (Fig. 4G, *MYC_174–196_KK + MYC_174–196(cold)). Notably, this peptide is lacking the C-terminal acceptor lysines and is therefore not a viable substrate for the ubiquitination reaction and reduction. As a consequence, the observed effect is likely explained by a direct competition for the UBR5 binding site rather than by depleting ATP and ubiquitin from the reaction condition. Finally, we utilized a control peptide carrying 6 amino acid substitutions within amino acids 181–189 that were designed to maintain the general physichochemical properties of the MYC peptide such as size, hydrophobicity, and polarity (L–V; S–T; S–T; E–D; C–S; I–L). The control peptide showed no enhanced ubiquitination after addition of UBR5 (2463–2792) (Fig. 4H), indicating that the interaction between the MYC peptide and UBR5 (2463–2792) is driven by sequence-specific interaction rather than by their general physichochemical properties.

Our data demonstrate that the HECT E3 ligase UBR5 interacts with MYC and enhances its K48 ubiquitination level to accelerate MYC degradation in cells. Direct interaction involving a linear MYC domain (aa 181–189) mediates this interaction with UBR5 (2463–2792).

### UBR5 depletion in cancers with MYC amplification results in reduced cell survival

Uncontrolled MYC expression and activity in cancer can occur due to copy number (CN) amplifications, chromosomal translocation events, or as a consequence of oncogenic or epigenetic events. More than half of human cancers exhibit high expression or activity of MYC^39,40^. However, acute and robust increase in MYC expression could reduce cellular fitness^17^. This was shown by Murphy et al.^16^, in an in vivo study using a switchable MycER^T2^ model, where tamoxifen-induced high level of Myc resulted in apoptosis through triggering the Arf/p53 tumor suppressor pathway in transformed murine cells. The difference in the regulation of MYC in malignant compared to normal cells was previously suggested to be due to additional loss of surveillance mechanisms in tumor cells (e.g. through p53 mutations) or by gain of pro-survival signals, such as BCL-2. This in turn allows malignant cells to proliferate in the presence of high MYC level.

Since the level of MYC in healthy cells typically does not reach levels that are high enough to induce apoptosis, this observation might provide a therapeutic window to target tumor cells with high MYC level while sparing healthy cells from apoptosis. Besides targeting above-mentioned pro-survival pathways to exploit this therapeutic window, direct intervention of MYC degradation mechanisms that lead to an acute rise in MYC level might also result in reduced cellular fitness.

**Figure 5:**
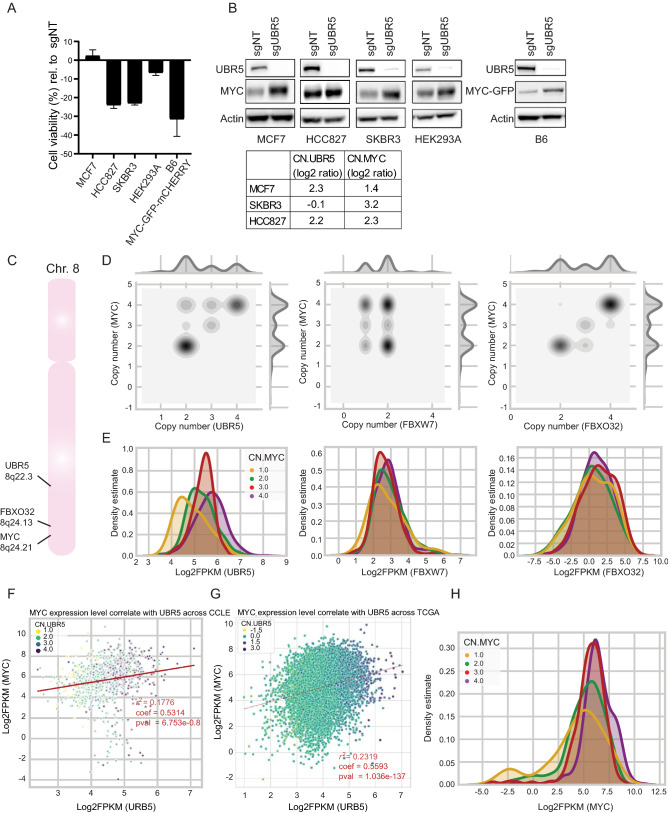
Targeting UBR5 in cell lines with MYC amplification. (**A**) Cell viability analysis using cell titer glow assay in cell lines upon knockout of UBR5. Data are normalized to cells transfected with sgNT. (**B**) Western blot analysis showing knockout of UBR5 and stabilization of MYC in cell lines with or without MYC amplification. (**C**) Localization of UBR5 (8q22.3) and FBXO32 (8q24.13) in proximity to MYC (8q24.21) on Chromosome 8, unlike FBXW7 (4q31.3, not shown). (**D**) Correlation of cell line copy number (CN) estimates of UBR5, FBXO32, and FBXW7 show co-amplification of UBR5 and FBXO32 with MYC amplifications. (**E**) Transcript-level distributions (shown as kernel density estimates of log2 FPKM values) of UBR5, FBXO32, and FBXW7 in cell lines as a function of MYC copy number demonstrate distinct expression upregulation of UBR5. (**F**) Transcriptional correlation of MYC and UBR5 level across CCLE. (**G**) Transcriptional correlation of MYC and UBR5 level across TCGA. (**H**) Transcript-level distributions of MYC as a function of MYC copy number.

Therefore, we hypothesized that the inhibition of UBR5 function in MYC-amplified cells and the consequential increase in MYC level could reduce cellular fitness in cancer cells with high MYC expression. To test this hypothesis and to take advantage of the role of UBR5 in MYC regulation, we assessed cell viability upon knockout of UBR5. We therefore compared cell lines with normal MYC CN (MCF7) to cells with MYC CN amplifications (HCC827, SKBR3). Indeed, upon knockout of UBR5, we observed reduced cell viability in the MYC CN-amplified cells HCC827 and SKBR3, whereas no reduction in viability was observed in MCF7 cells (Fig. 5A,B). Furthermore, we assessed the effect of UBR5 knockout on wild type HEK293A cells and compared it to clone B6, in which the MYC-sensor was overexpressed, thus representing a cell line with synthetically induced high MYC expression. Interestingly, also in this setting we find a significant reduction in cell viability in the B6 clone in comparison to wild type HEK293A cells. Collectively, our results demonstrate that cells with high MYC expression levels depend on the presence of UBR5 to keep MYC protein under control, and that the further boost in MYC protein level following loss of UBR5 might be one of the reasons for suppressed proliferation observed in this context. We therefore suggest that inhibition of UBR5 might provide a novel therapeutic window in targeting cancer cell lines with MYC amplification.

Interestingly, *MYC (8q24.21)* and *UBR5* *(8q22.3)* are both reported to co-localize on chromosome 8q. Another ligase gene that has been suggested as a MYC regulator^36^ *FBXO32* is localized between *MYC* and *UBR5* *(8q24.13)* (Fig. 5C), as opposed to *FBXW7* that is localized on *4q31.3*. To gain further insight into this observation, we analyzed cell lines from the Cancer Cell Line Encyclopedia (CCLE^41^) from various cancer indications and found that *MYC* CN correlates to *UBR5* CN, with 43% co-amplifications and even stronger to *FBXO32* CN with 53% co-amplifications (Fig. 5D and supplementary table 2). This correlation could be due to co-amplification events. In addition, since the *FBXO32* locus is closer to the *MYC* locus than the *UBR5* locus, its correlation to *MYC* CN is stronger. As expected, we did not observe any correlation in *MYC* CN to *FBXW7* CN since those genes are localized on different chromosomes.

We further analyzed the effect of *MYC* CN on the RNA expression level of the respective E3 ligases (UBR5, FBXO32, and FBXW7). Strikingly, we do observe increased UBR5 expression level when *MYC* CN is increased (Fig. 5E). No such correlation was observed for FBXO32, even though it shows a stronger CN to CN correlation with *MYC* than *UBR5*. We also did not identify an increase in FBXW7 RNA level upon increase in *MYC* CN. This data suggest that *UBR5* expression is connected to *MYC* CN amplification. Here, UBR5 might act as a buffer E3 ligase controlling MYC protein level to prevent an additional MYC increase, thereby protecting the cells from apoptosis. Further, we performed correlation analysis of UBR5 RNA expression level to MYC expression level using data collected from the CCLE (Fig. 5F) as well as the cancer genome atlas (TCGA) program (Fig. 5G). Our data show a trend in the correlation in MYC RNA level to UBR5 with a higher significance when using TCGA data. The lower correlation between UBR5 RNA and MYC RNA level (Fig. 5F,G) in comparison to *UBR5* and *MYC* CN (Fig. 5E) is due to poor correlation between MYC RNA expression to its own CN (Fig. 5H). This is probably due to the regulation of MYC activity on multiple level as extensively published and reviewed elsewhere^6,15,42,43^. Interestingly, we analyzed ChIP-sequencing data available from the Cistrome database (www.cistrome.org/db) and identified a MYC-binding site upstream of *UBR5* promoter transcription start site across different cell lines and cancer indications (Supplementary Fig. 5), suggesting a potential regulatory feedback loop in some indications.

In this study and in accordance with the previous study from Qiao et al.^19^, we propose a novel mechanism of MYC regulation through UBR5 that provides a potential therapeutic target in *MYC*-amplified cancers. As a HECT E3 ligase, UBR5 has its own catalytic cysteine to accept ubiquitin from the E2 enzyme before ligating it to its substrate (MYC), providing a starting point for the development of specific therapeutic interventions. Since the catalytic cysteine C2768 of UBR5 has been described previously^44^, it could be used as a specific site for the discovery of a covalent inhibitor.

## Materials and methods

### Cell line and reagents

The following cell lines (HCC827, SKBR3, and MCF7) were obtained from the original CCLE collection^41^ and maintained under standard conditions (37 °C, 5% CO2). HEK293A (#R70507, Invitrogen) were cultured in DMEM, supplemented with 10% Fetal Calf Serum (FCS), HCC827 cells were cultured in RPMI1640 (Gibco, #61870-010), 20% FCS, 10 mM HEPES, 1 mM Sodium Pyruvate. SKBR3, and MCF7 were cultured in RPMI1640 (Gibco, #61870-010), 20% FCS, 10 μg/mL human insulin (Sigma Aldrich, #I9278). Single cell clone B6 was cultured in HEK293A cell culture media, supplemented with 10 μg/mL blasticidin and 300ug/mL hygromycin.

Cycloheximide was purchased from Sigma Aldrich (#1001316970), MLN4924 from R&D systems Inc. (#I-502-01M), and Bortezomib from Sigma Aldrich (#5043140001) and were used at [20 μM], [1 μM], and [50 nM], respectively.

### Immunoprecipitation and western blot analysis

Mammalian cells were either treated with indicated compounds or transfected with sgRNA/Cas9 and collected for western blot (WB) or immunoprecipitation (IP) analysis. Therefore, cells were washed with 1 × PBS and lysed in RIPA lysis buffer 10x (#20-188, Millipore), diluted in H_2_O and supplemented with PhosSTOP EASYpack (#04906837001, Roche) and Complete Mini-Protease inhibitor cocktail tablets (11836153001, Roche). Protein concentrations were then determined using a BSA kit (#23225, Pierce). For gel electrophoresis, samples were added NuPAGE Sample Reducing Agent (10X, Invitrogen) diluted 1:10 with NuPAGE LDS Sample Buffer (4x, Invitrogen) and then boiled at 70 °C for 10 min. Proteins were resolved by SDS-PAGE using mini-gels and transferred to a PVDF membrane using wet blotting system (Invitrogen). Membranes were blocked in 5% Milk-PBS-Tween 20 for 1 h and incubated with primary antibody over night at 4 °C. Secondary antibodies were incubated for 1 h at room temperature (RT) before blots were developed using the detection kit SuperSignal West Femto Maximum Sensitivity Substrate (Thermo Fisher, #34096). Blots were either exposed on film or on a western blot imager (Fusion FX, Vilber). The following antibodies were used for the analysis: MYC, rabbit mAb (Abcam, #3ab2072); Actin, mouse mAb (Millipore Sigma, MAB1501); UBR5 (D6O8Z), rabbit mAb (Cell Signaling Technologies (CST), #65344), GFP, mouse mAb (Roche, #11814460001); V5, mouse mAb (Invitrogen, #R960-25); K48-linkage Specific Polyubiquitin (D9D5) mAb (CST, #8081); secondary antibody: anti-rabbit, goat pAb to Rb-IgG (Abcam, #7090); secondary antibody: anti-mouse, sheep IgG HRP-linked (GE healthcare, #NA931V).

Immunoprecipitation (IP) was performed on lysed samples (described above) using the ChromoTek GFP-Trap affinity resin (Chromotek, #gta-20) following manufacturer’s instructions.

A full-scan of all performed western blots is provided in Supplementary Fig. 6.

### Quantitative real-time PCR

For mRNA expression analysis, total RNA was extracted from cells using an RNeasy mini kit (Qiagen, #74136). Real time PCR was then performed using iTaq Universal Probes One-Step Kit (Biorad, #172-5140) according to manufacturer’s instruction. Expression was normalized to human 18S. The following predesigned labeled primers and probe sets (Taqman assays) from Integrated DNA Technologies (IDT) were used: h18S #HS.PT.39a.22214856 g, hMYC #Hs.PT.58.26770695, hAPEX1 #Hs.PT.56a.3182919, hCDC25A #Hs.PT.58.800341, hCAD #Hs.PT.56a.39091521.

### Cell viability assay

To assess cell growth, cells were plated in 96-well plates at 10.000 cells/well and treated according to the experiment. At indicated time points, cells were lysed using CellTiter-Glo Luminescent Cell Viability Assay (Promega, #G7572) followed by luminescence measurements using a luminescence reader (Berthold). The data are normalized to the respective control for each experiment.

### Pooled CRISPR screen

#### Screening procedure

A genetic knockout screen using CRISPR/Cas9 and targeting the proteasomal pathway components (750 E3s, 100 DUBs, 40 E2s, 8 E1s and 7 regulators, 14 proteasomal genes, controls) was performed in B6 single cell clone (HEK293A, stably expressing MYC-GFP-chysel-mCHERRY and SpCas9). The sgRNA library contained 10 sgRNAs per gene and was packaged in lentiviruses. Single cell clone B6 was infected with the sgRNA library and selected with puromycin over seven or 14 days. On day seven or 14, cells were sorted based on the GFP/mCHERRY ratio into three bins. The outer most bins, representing high GFP/mCHERRY or low GFP/mCHERRY contained 15% of the total population, respectively, whereas the inner bin with no changes in GFP/mCHERRY ratio contained the remaining cells of the sorted population. The enrichment of sgRNA in each bin was assessed using next-generation sequencing (NGS) and compared to the total sgRNA abundance in the unsorted cell population on the respective day.

#### Quantification and statistical analysis

The score for each sgRNA was quantified using Bioconductor package edgeR^45^. Counts for all samples (low and high GFP/mCHERRY ratio, unsorted cell populations) were normalized using the Trimmed Mean of M-values (TMM) normalization. A counts per million (CPM) value was then calculated for each sgRNA in each sample. At day 7 and day 14, the sgRNA abundance in high and low populations were compared to unsorted populations, calculated using the general linear model log likelihood ratio test (glmLRT) method in edgeR. This corresponds to the log fold change (LogFC) enrichment of each sgRNA in each cell population. A gene level call was taken as the median LogFC of the 10 sgRNAs targeting the respective gene. In addition, an enrichment score of each gene in each population was assessed using the redundant siRNA activity (RSA) concept^46^. RSA uses all the sgRNAs per gene to provide a measure of the statistical significance of the enrichment of those sgRNAs in each population. The method is directional and was used to capture overrepresented genes only.

#### Lethal gene identification

Based on project DRIVE^29^ (a large RNAi screen in which viability effects of mRNA knockdown were assessed for 7837 genes using an average of 20 shRNAs per gene in 398 cancer cell lines), a gene was classified as “Lethal” if it had a median RSA sensitizing^46^ score of <  − 7, indicating that the knockdown of that gene is lethal in most of the cell lines screened.

### Arrayed CRISPR screen

Individually arrayed sgRNA-library targeting HECT E3 ligases were prepared in a 96-well plate. Two sgRNAs per gene were used as an average to assess the knockout effect on MYC protein modulation. Viral particles were produced in 96-well plates and used for infection. B6 cells were plated in 96-well plates and infected with the prepared virus. 24 h post-transfection, media was replaced with puromycin-supplemented medium and cells were incubated for 7 days. On day 7, FACS-based analysis were performed to assess GFP/mCHERRY in knockout cells. Acquired data was normalized to GFP/mCHERRY signal in B6 cells infected with sgNT expressing virus.

### In vitro validation assays through genetic knockdown or knockout

#### siRNA-mediated knockdown

Mammalian cells were plated either in 96-well plates or 6-well plates for 24 h and transfected with a pool of two individual siRNAs targeting UBR5 (Dharmacon, #J-007189-06 (GCACUUAUAUACUGGAUUA), #J-007189-07 (GAUUGUAGGUUACUUAGAA)) using Lipofectamine RNAiMAX (Invitrogen, #13778030) according to manufacturer’s instructions. 24 h post-transfection, medium was changed and cells were collected for WB, FACS, or qRT-PCR analysis where indicated at different time points.

#### sgRNA-mediated knockout

A pool of three individually designed sgRNAs targeting UBR5 (CAGACTTACTCTGAACTTCA, AGCTGGAAAACAGTTTATAT, TATTCTTTAGGTTGCTACAT) was premixed with purified SpCas9 at RT and electroporated into mammalian cells (HEK293A—wild type or clone B6) using the neon transfection system (Invitrogen, #MPK1025) according to manufacturer’s instructions. Cells were harvested at indicated time points for further analysis. Knockout of GFP (sgGFP: GGTGAACCGCATCGAGCTGA), mCHERRY (sgmCHERRY: CGAGTTCGAGATCGAGGGCG), or the non-targeting control (sgNT: ACGGAGGCTAAGCGTCGCAA) was performed using the same method.

### Confocal microscopy

In order to detect localization or GFP-tagged MYC as well as mCHERRY, cells were transfected with the respective constructs using Lipofectamine 3000 reagent (Invitrogen, #L3000008) according to manufacturer’s instructions. After 24 h of transfection, medium was replaced with Hoechst (Invitrogen, #H2570) containing medium at 10 μg/mL. Images were acquired using the confocal microscope Yokogawa CV7000. Image analysis was conducted with the CV7000 Analysis software (version 2.08.08).

### MYC-GFP protein abundance flow cytometry assay in HEK293A cells

Protein level of MYC were measured in HEK293A cells expressing either MYC-GFP and mCHERRY or GFP-MYC and mCHERRY from a stably integrated bicistronic MYC-GFP-chysel-mCHERRY or mCHERRY-chysel-GFP-MYC 293A cell lines, respectively. Changes in the GFP signal measured by flow cytometry served as readout for MYC protein abundance.

### MYC sensor generations and library construction

#### Cloning of the pLenti6-MYC-GFP-chysel-mCHERRY, mCHERRY-chysel-GFP-MYC sensor vectors

The bicistronic GFP-chysel-mCHERRY constructs are based on a pLenti6-DEST vector backbone where two cassettes were introduced: Xho1-GFP-chysel-mCHERRY-Xho1 or Spe1-mCHERRY-chysel-GFP-Spe1 to generate either pL6-CMV-DEST-GFP-chysel-mCHERRY or pL6-CMV-mCHERRY-chysel-GFP-DEST destination vectors for LR cloning, respectively. Both cassettes were synthesized by an external vendor (GeneART) and cloned into linearized pLenti6-DEST vector using Gibson assembly (GA) according to manufacturer’s instructions (New England Biolabs, #E5510). Linearization of pLenti6-DEST with Xho1 and GA with Xho1-GFP-chysel-mCHERRY-Xho1 fragment resulted in gateway compatible pLenti6-DEST-GFP-chysel-mCHERRY vector, linearization of pLenti6-DEST with Spe1 and GA with Spe1-mCHERRY-chysel-GFP-Spe1 fragment resulted in gateway compatible pLenti6-mCHERRY-chysel-GFP-DEST vector.

pLenti6-mCHERRY-chysel-GFP-MYC was generated by gateway LR cloning between pENTR221-MYC and pLenti6-mCHERRY-chysel-GFP-DEST vectors. To clone the pLenti6-MYC-GFP-chysel-mCHERRY sensor vector, the STOP codon was mutated to a Leucine using a Quickchange reaction (Agilent Technologies, #210518) on the existing pENTR221-MYC vector with the following primers (pENTR221_MYC_Quikchange_STOP-LEU_fw, cagctgcggaactcctgcgccttgtacccagctttcttgtac, pENTR221_MYC_Quikchange_STOP-LEU_rev, gtacaagaaagctgggtacaaggcgcaggagttccgcagctg). LR gateway cloning between pLenti6-DEST-GFP-chysel-mCHERRY with pENTR221-MYC(STOP-Leu) vectors resulted in pLenti6-MYC-GFP-chysel-mCHERRY. All vectors described have been sequenced for verification.

#### Engineering of stably expressing HEK293A MYC-GFP-chysel-mCHERRY and mCHERRY-chysel-GFP-MYC sensor cells

HEK293A MYC-GFP-chysel-mCHERRY and mCHERRY-chysel-GFP-MYC sensor cells were generated by lentiviral vector transduction using the MYC sensor construct described above. Lentiviral particles were produced in HEK293FT cells (ATCC, #CRL-112268) by co-transfection of 500 ng pLenti6-MYC-GFP-chysel-mCHERRY, 500 ng delta8.71 and 200 ng pVSVG diluted in 100μL OptiMEM serum free medium (Invitrogen, #11058-021) that was mixed after 5 min preincubation with 3μL of Lipofectamine2000 (Invitrogen, #11668-019) in 97μL OptiMEM serum free medium. The mix was incubated for 20 min at RT and then added to 1 mL of a freshly prepared suspension of HEK293FT cells in a well of a 6-well plate (concentration 1.2 × 106 cells/mL). 24 h after transfection, the medium was replaced with 1.5 mL of complete growth medium (DMEM high Glucose + 10% FCS + 1% L-Glutamine + 1% NEAA + 1% NaPyr.). 48 h post transfection, supernatant containing viral transducing particles was collected and frozen at − 80 °C.

24 h before transduction with viral particles, 1 × 10^5^ HEK293A cells were seeded in 2 mL growth medium in a well of a 6-well plate. Infection was performed with 90μL of collected supernatant containing viral transducing particles in 1 mL medium including 8 μg/mL polybrene. 24 h post infection, stably transfected cells were selected with blasticidin at a concentration of 10 μg/mL referred to as stable HEK293A sensor cells (B6 clone).

### Biochemical UBR5 MYC peptide ubiquitination assay

#### Molecular cloning of UBR5 (2473–2792) and UBE2D2

The DNA sequence encoding E3 ubiquitin-protein ligase UBR5 (2473–2792) was amplified from plasmid DNA and cloned into a pET-derived expression vector. The resulting construct encoded an N-terminal hexahistidine affinity tag followed by a Human Rhinovirus (HRV) 3C cleavage site and the UBR5 HECT domain. DNA encoding full-length Ubiquitin-conjugating enzyme E2 D2 (UBE2D2) was amplified from plasmid DNA and cloned into a pET-derived expression vector. The resulting construct encoded an N-terminal Rubredoxin polypeptide followed by a hexahistidine affinity tag, a 3C cleavage site, and full-length UBE2D2.

#### Protein expression and purification

##### UBR5

The plasmid was transformed into *E. coli* BL21 DE3 cells and cells were grown in TB-MOPS shake culture at 37 °C in the presence of 50 µg/mL kanamycin until the culture reached an optical density of ~ 0.6. Protein expression was induced with 150 µM IPTG and cells were grown at 18 °C over night. Cells were harvested, resuspended in 50 mL buffer A (500 mM NaCl, 50 mM Hepes pH 8.0, 1 mM DTT, 5 mM imidazol, supplemented with one tablet Roche complete Protease Inhibitor Cocktail) and subsequently lyzed by sonication. Lysate was centrifuged for at 1 h at 47,000 rcf and cleared lysate was loaded on a 5 mL QIAGEN Ni–NTA superflow cartridge using a peristaltic pump. The column was washed with 10 CV Buffer A and bound protein was eluted with 5 CV buffer A supplemented with 250 mM imidazol. Eluted protein was incubated with 1% w/w 3C protease at room temperature for 2 h and was subsequently subjected to size exclusion chromatography utilizing a Superdex S75 26/600 column (GE Healthcare) in 500 mM NaCl and 50 mM Hepes pH 7.5. Protein homogeneity and correct mass were verified by SDS-PAGE and LC–MS analysis. Protein was concentrated using a 10,000 MWCO centrifugal concentrator and was subsequently flash-frozen in liquid nitrogen and stored at − 80 °C.

##### UBE2D2

The plasmid was transformed into *E. coli* BL21 DE3 cells and cells were grown at 37 °C in TB-MOPS auto-inducing medium in a 1.5 L fermenter in the presence of 50 µg/mL kanamycin. Cells were grown to an optical density of ~ 6 and then temperature was reduced to 18 °C and cells were grown over night. Cells were harvested, resuspended in 400 mL buffer A (500 mM NaCl, 50 mM Hepes pH 8.0, 1 mM DTT, 5 mM imidazol, supplemented with two tablets Roche complete Protease Inhibitor Cocktail) and subsequently lyzed by high pressure homogenization. Lysate was cleared by centrifugation for 1 h at 47,000 rcf and cleared lysate was loaded on a tandem 5 mL QIAGEN Ni–NTA Superflow cartridge with a peristaltic pump. The columns were washed with 10 CV Buffer A and bound protein was eluted with 5 CV buffer A supplemented with 250 mM imidazol. Eluted protein was pooled, supplemented with 1% w/w 3C protease and dialyzed against 2 L Buffer B (500 mM NaCl, 20 mM Hepes pH 7.5, 1 mM DTT) using 3000 MWCO dialysis cassettes (Slide-A-Lyzer, Thermo Fisher Scientific). To remove the Rubredoxin and hexahistidine tag, the dialyzed and cleaved protein was again loaded on a tandem 5 mL QIAGEN Ni–NTA Superflow cartridge and flow-through was collected and concentrated with a 10,000 MWCO centrifugal concentrator. The concentrated protein was subjected to size exclusion chromatography on a Superdex S75 26/600 column (GE Healthcare) in 500 mM NaCl and 20 mM Hepes pH 7.5. Eluted fractions were pooled, concentrated with 10,000 MWCO centrifugal concentrators and subsequently flash-frozen in liquid nitrogen and stored at − 80 °C.

##### UBA1

Ubiquitin-like modifier-activating enzyme 1 was a generous gift from Laura Tandeske (Novartis, Emeryville). Briefly, UBA1 was expressed in *E. coli* BL21 DE3 cells and cells were lysed by high-pressure homogenization and subsequently purified by immobilized metal ion affinity chromatography and size exclusion chromatography.

#### Ubiquitination assay

Ubiquitination assays were performed by mixing UBA1 (0.6 µM), UBE2D2 (1 µM), UBR5 (1 µM), fluorescein-labelled peptide substrate (400 nM), and Ubiquitin (5 µM) in reaction buffer (100 mM NaCl, 20 mM Hepes pH 7.5, 1 mM MgCl_2_, 5 mM ATP, 1 mM TCEP). Aliquots were taken at indicated time points and reactions were quenched by addition of equal volume SDS loading buffer (250 mM Tris pH 6.8, 10% w/v SDS, 200 mM 2-mercaptoethanol, and 40% glycerol). Signal quantification on gels to calculate fold induction has been performed using ImageJ software (https://imagej.nih.gov/ij/—ImageJ 1.51n).

#### Synthetic peptides

Peptide substrates MYC_174–196_KK (Fluo-SSLYLQDLSAAASECIDPSVVFPKK), MYC-174–196-KK^Mut^ (Fluo-SSLYLQDVTAAATDSLDPSVVFPKK) and MYC_174–196 (SSLYLQDLSAAASECIDPSVVFP) were synthesized by Biosyntan (Berlin, Germany). Peptide purity and mass were confirmed by HPLC and LC–MS analysis.

### Correlation analysis of MYC copy number and RNA expression to UBR5, FBXO32, and FBXW7

#### Copy number estimates

Copy numbers are estimated from the mean log2 ratio signal values determined from Affymetrix SNP 6.0 microarray based on the formula $CN = round\left[ {2^{{log_{2} \left( {\frac{sample}{{reference}} + 1} \right)}} } \right]$ resulting in gene-level integer estimates.

#### Transcriptional expression aggregates

Gene-level expression aggregates are based on log2 transformed FPKM (fragments per kilobase of transcript per million sample reads) values. Correlation analysis follows a simple pairwise linear regression for each gene pair. Kernel density estimates are based on a non-parametric Gaussian distribution model.

## Supplementary information

Supplementary Table 1.

Supplementary Information 1.

Supplementary Information 2.

## Data Availability

All data pertaining to this study are in the paper check with ms.

## References

[1] Vogel C, Marcotte EM (2012). Insights into the regulation of protein abundance from proteomic and transcriptomic analyses. Nat. Rev. Genet..

[2] Varshavsky A (2017). The ubiquitin system, autophagy, and regulated protein degradation. Annu. Rev. Biochem..

[3] Westermarck J (2010). Regulation of transcription factor function by targeted protein degradation: an overview focusing on p53, c-Myc, and c-Jun. Methods Mol. Biol..

[4] Lee TI, Young RA (2013). Transcriptional regulation and its misregulation in disease. Cell.

[5] Bushweller JH (2019). Targeting transcription factors in cancer—from undruggable to reality. Nat. Rev. Cancer.

[6] Miller DM, Thomas SD, Islam A, Muench D, Sedoris K (2012). c-Myc and cancer metabolism. Clin. Cancer Res..

[7] Yada M (2004). Phosphorylation-dependent degradation of c-Myc is mediated by the F-box protein Fbw7. EMBO J..

[8] Welcker M (2004). The Fbw7 tumor suppressor regulates glycogen synthase kinase 3 phosphorylation-dependent c-Myc protein degradation. Proc. Natl. Acad. Sci. USA.

[9] Xu G, Jaffrey SR (2011). The new landscape of protein ubiquitination. Nat. Biotechnol..

[10] Swatek KN, Komander D (2016). Ubiquitin modifications. Cell Res..

[11] Li W, Ye Y (2008). Polyubiquitin chains: functions, structures, and mechanisms. Cell Mol. Life Sci..

[12] Hershko A, Ciechanover A, Varshavsky A (2000). Basic medical research award. The ubiquitin system. Nat. Med..

[13] Berndsen CE, Wolberger C (2014). New insights into ubiquitin E3 ligase mechanism. Nat. Struct. Mol. Biol..

[14] Jang HH (2018). Regulation of protein degradation by proteasomes in cancer. J. Cancer Prev..

[15] Gabay M, Li Y, Felsher DW (2014). MYC activation is a hallmark of cancer initiation and maintenance. Cold Spring Harb. Perspect. Med..

[16] Murphy DJ (2008). Distinct thresholds govern Myc's biological output in vivo. Cancer Cell.

[17] McMahon SB (2014). MYC and the control of apoptosis. Cold Spring Harb. Perspect. Med..

[18] Lowe SW, Cepero E, Evan G (2004). Intrinsic tumour suppression. Nature.

[19] Qiao X (2020). UBR5 is co-amplified with MYC in breast tumors and encodes an ubiquitin ligase that limits MYC-dependent apoptosis. Cancer Res..

[20] Emanuele MJ (2011). Global identification of modular cullin-RING ligase substrates. Cell.

[21] Yen HC, Xu Q, Chou DM, Zhao Z, Elledge SJ (2008). Global protein stability profiling in mammalian cells. Science.

[22] Lo CA (2015). Quantification of protein levels in single living cells. Cell Rep..

[23] Yau EH, Rana TM (2018). Next-generation sequencing of genome-wide CRISPR screens. Methods Mol. Biol..

[24] Arabi A, Rustum C, Hallberg E, Wright AP (2003). Accumulation of c-Myc and proteasomes at the nucleoli of cells containing elevated c-Myc protein levels. J. Cell Sci..

[25] Bennett EJ, Rush J, Gygi SP, Harper JW (2010). Dynamics of cullin-RING ubiquitin ligase network revealed by systematic quantitative proteomics. Cell.

[26] Yang Z (2019). Inhibition of neddylation modification by MLN4924 sensitizes hepatocellular carcinoma cells to sorafenib. Oncol Rep..

[27] Penn LJ, Brooks MW, Laufer EM, Land H (1990). Negative autoregulation of c-myc transcription. EMBO J..

[28] Liu H (2019). Tumor-derived IFN triggers chronic pathway agonism and sensitivity to ADAR loss. Nat. Med..

[29] McDonald ER (2017). Project DRIVE: a compendium of cancer dependencies and synthetic lethal relationships uncovered by large-scale. Deep RNAi Screen. Cell.

[30] Koren I (2018). The eukaryotic proteome is shaped by E3 ubiquitin ligases targeting C-terminal degrons. Cell.

[31] Schwechheimer C (2018). NEDD8-its role in the regulation of Cullin-RING ligases. Curr. Opin. Plant Biol..

[32] Petroski MD, Deshaies RJ (2005). Function and regulation of cullin-RING ubiquitin ligases. Nat. Rev. Mol. Cell Biol..

[33] Sievers QL, Gasser JA, Cowley GS, Fischer ES, Ebert BL (2018). Genome-wide screen identifies cullin-RING ligase machinery required for lenalidomide-dependent CRL4(CRBN) activity. Blood.

[34] Kim D (2017). Deubiquitinating enzyme USP22 positively regulates c-Myc stability and tumorigenic activity in mammalian and breast cancer cells. J. Cell Physiol..

[35] Popov N (2007). The ubiquitin-specific protease USP28 is required for MYC stability. Nat. Cell Biol..

[36] Mei Z (2015). FBXO32 targets c-Myc for proteasomal degradation and inhibits c-Myc activity. J. Biol. Chem..

[37] Fang X (2017). Deubiquitinase USP13 maintains glioblastoma stem cells by antagonizing FBXL14-mediated Myc ubiquitination. J. Exp. Med..

[38] Callaghan MJ (1998). Identification of a human HECT family protein with homology to the Drosophila tumor suppressor gene hyperplastic discs. Oncogene.

[39] Beroukhim R (2010). The landscape of somatic copy-number alteration across human cancers. Nature.

[40] Ciriello G (2013). Emerging landscape of oncogenic signatures across human cancers. Nat. Genet..

[41] Barretina J (2012). The cancer cell line encyclopedia enables predictive modelling of anticancer drug sensitivity. Nature.

[42] Dang CV (2012). MYC on the path to cancer. Cell.

[43] Baluapuri A, Wolf E, Eilers M (2020). Target gene-independent functions of MYC oncoproteins. Nat. Rev. Mol. Cell Biol..

[44] Matta-Camacho E, Kozlov G, Menade M, Gehring K (2012). Structure of the HECT C-lobe of the UBR5 E3 ubiquitin ligase. Acta Crystallogr. Sect. F Struct. Biol. Cryst. Commun..

[45] Robinson MD, McCarthy DJ, Smyth GK (2010). edgeR: a Bioconductor package for differential expression analysis of digital gene expression data. Bioinformatics.

[46] Konig R (2007). A probability-based approach for the analysis of large-scale RNAi screens. Nat. Methods.

