# Supplementary material for: Identification of the HECT E3 ligase UBR5 as a regulator of MYC degradation using a CRISPR/Cas9 screen
Source: Sci Rep. 2020 Nov 18;10:20044. doi: 10.1038/s41598-020-76960-z (PMC7676242; doi:10.1038/s41598-020-76960-z)
Supplement: Supplementary file 3 — Supplementary Information 2. [file 41598_2020_76960_MOESM3_ESM.docx]

**Supplementary information:**

**Identification of the HECT E3 ligase UBR5 as a regulator of MYC degradation using a CRISPR/Cas9 screen**

Lina Schukur^1,*,+^, Tamara Zimmermann^1^, Ole Niewoehner^2^, Grainne Kerr^1^, Scott Gleim^2^, Beatrice Bauer-Probst^1^, Britta Knapp^2^, Giorgio G. Galli^1^, Xiaoyou Liang^2^, Angelica Mendiola^3^, John Reece-Hoyes^2^, Melivoia Rapti^1^, Ines Barbosa^1^, Markus Reschke^1,#^, Thomas Radimerski^1,≠,#^ and Claudio R. Thoma^2,#,*^

^1^Novartis Institutes for Biomedical Research (NIBR) Oncology, Novartis; ^2^NIBR Chemical Biology and Therapeutics, Novartis; ^3^Genomics Institute of the Novartis Research Foundation; ^≠^present address: Basilea Pharmaceutica; +present address: Pharmaceutical Research and Early Development, Roche Innovation Center Basel, F. Hoffmann-La Roche Ltd

^#^co-last author; ^*^co-correspondence

Corresponding authors: [claudio.thoma@novartis.com](mailto:claudio.thoma@novartis.com) and [lina.schukur@roche.com](mailto:lina.schukur@roche.com)


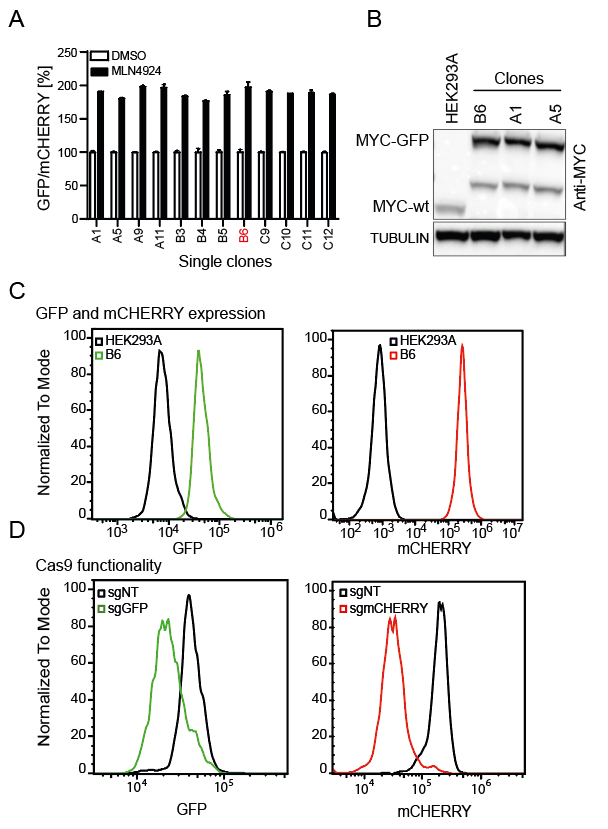


Supplementary figure 1: Single cell clone selection and characterization. (**A**) Single cell clones were generated from multi-clonal HEK293A cell population, stably expressing C-terminal GFP-chysel-mCHERRY MYC tag and Cas9 protein. Selected clones are treated with 1µM MLN4924 for 24h followed by FACS analysis. (**B**) Western blot analysis showing MYC expression with or without GFP fusion in HEK293A wild type cells compared to clone B6, A1 and A5. (**C**) FACS-measured GFP and mCHERRY expression level of clone B6. (**D**) Cas9 functional assay with decrease in GFP and mCHERRY ratio upon transfection with sgRNA targeting GFP (sgGFP) or mCHERRY (sgmCHERRY), respectively.


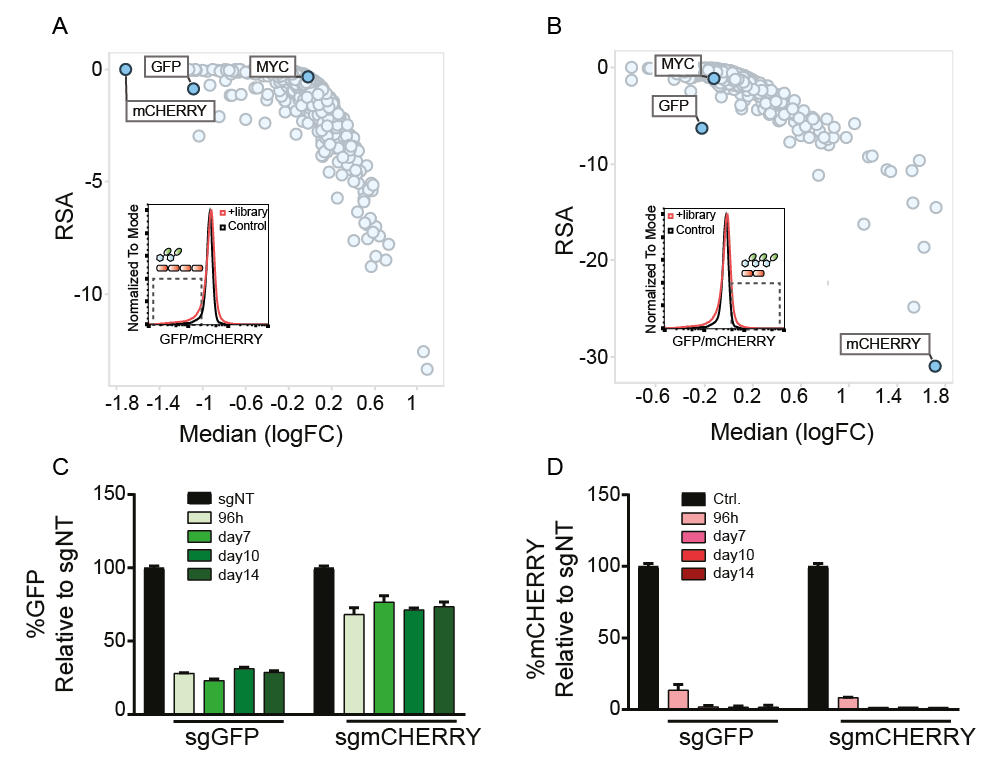
Supplementary figure 2: Enrichment and depletion of control genes in the UPS screen. (**A**) Plots showing gene enrichment score in low GFP/mCHERRY (A) or high GFP/mCHERRY (**B**)-sorted cells relative to the unsorted cell population. Values are visualized as LogFC. Relative changes of GFP (**C**) or mCHERRY (**D**) in B6 clone transfected with sgRNA targeting GFP (sgGFP) and mCHERRY (sgmCHERRY) over time.


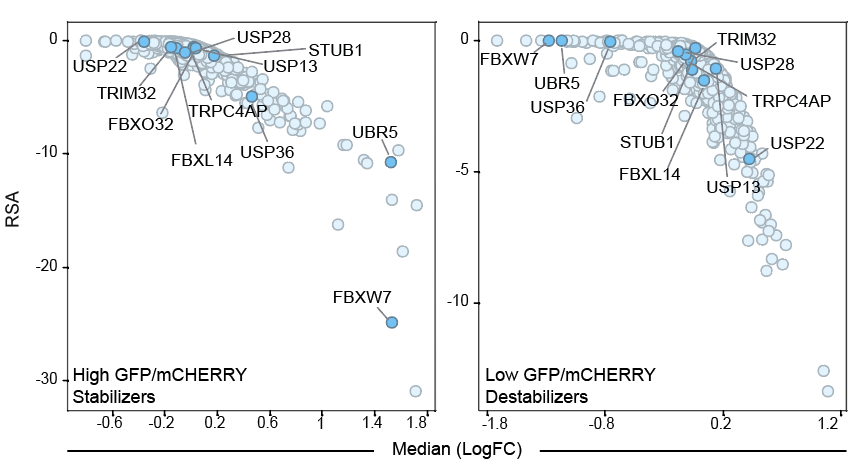
Supplementary figure 3: Enrichment of published MYC regulators. Plots showing gene enrichment score in high or low GFP/mCHERRY-sorted cells relative to unsorted cell population comparing published MYC regulators. Values are visualized as LogFC.


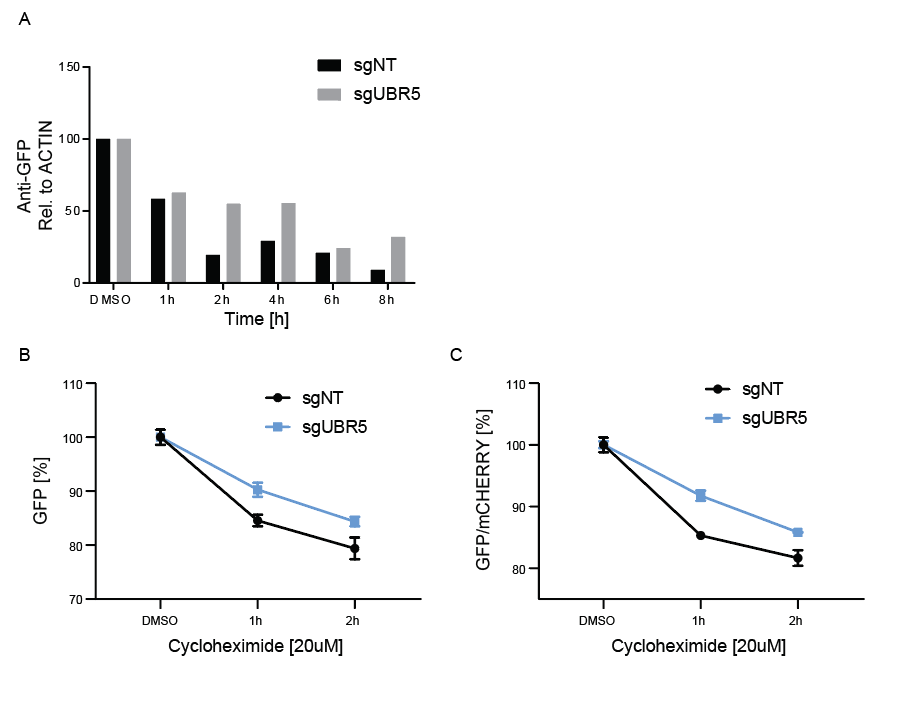


Supplementary figure 4: Quantification analysis of MYC-GFP stabilization and increase in MYC half-life upon knockout of UBR5. (**A**) WB quantification analysis from Figure 4A. (**B, C**) Single cell clone B6 was transfected with sgNT, or sgUBR5 and treated with cycloheximide. Intensity of GFP or GFP/mCHERRY was measured using FACS at indicated time points of cycloheximide treatment and normalized to DMSO.


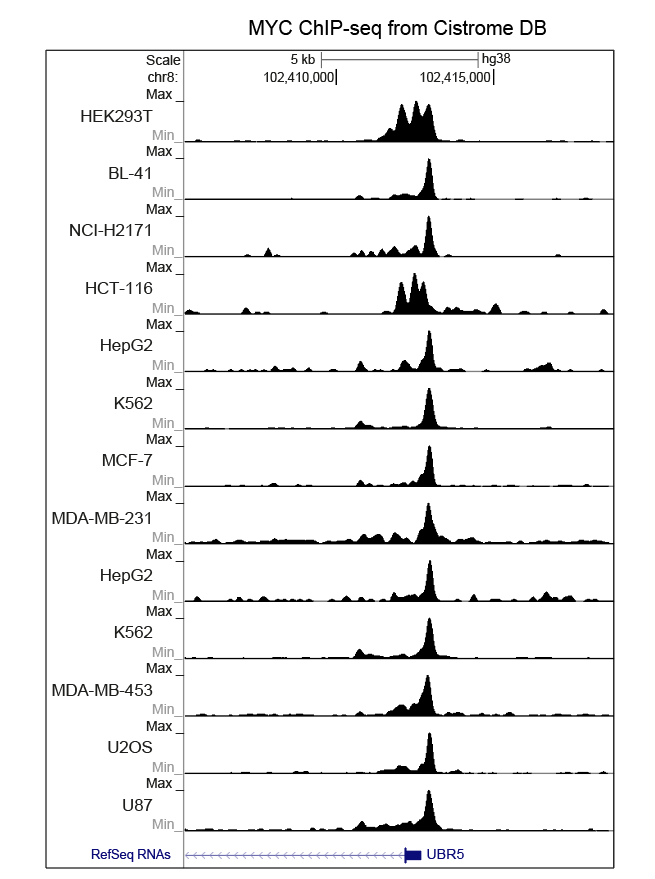


Supplementary figure 5: MYC binding sites identified upstream of UBR5 transcription start site. ChIP-sequencing data available from the Cistrome database ([www.cistrome.org/db](http://www.cistrome.org/db)) were used to analyze MYC-binding sites within the UBR5 promoter region. We used the UCSC genome browser ([www.genome.ucsc.edu](http://www.genome.ucsc.edu/)) for visualization.


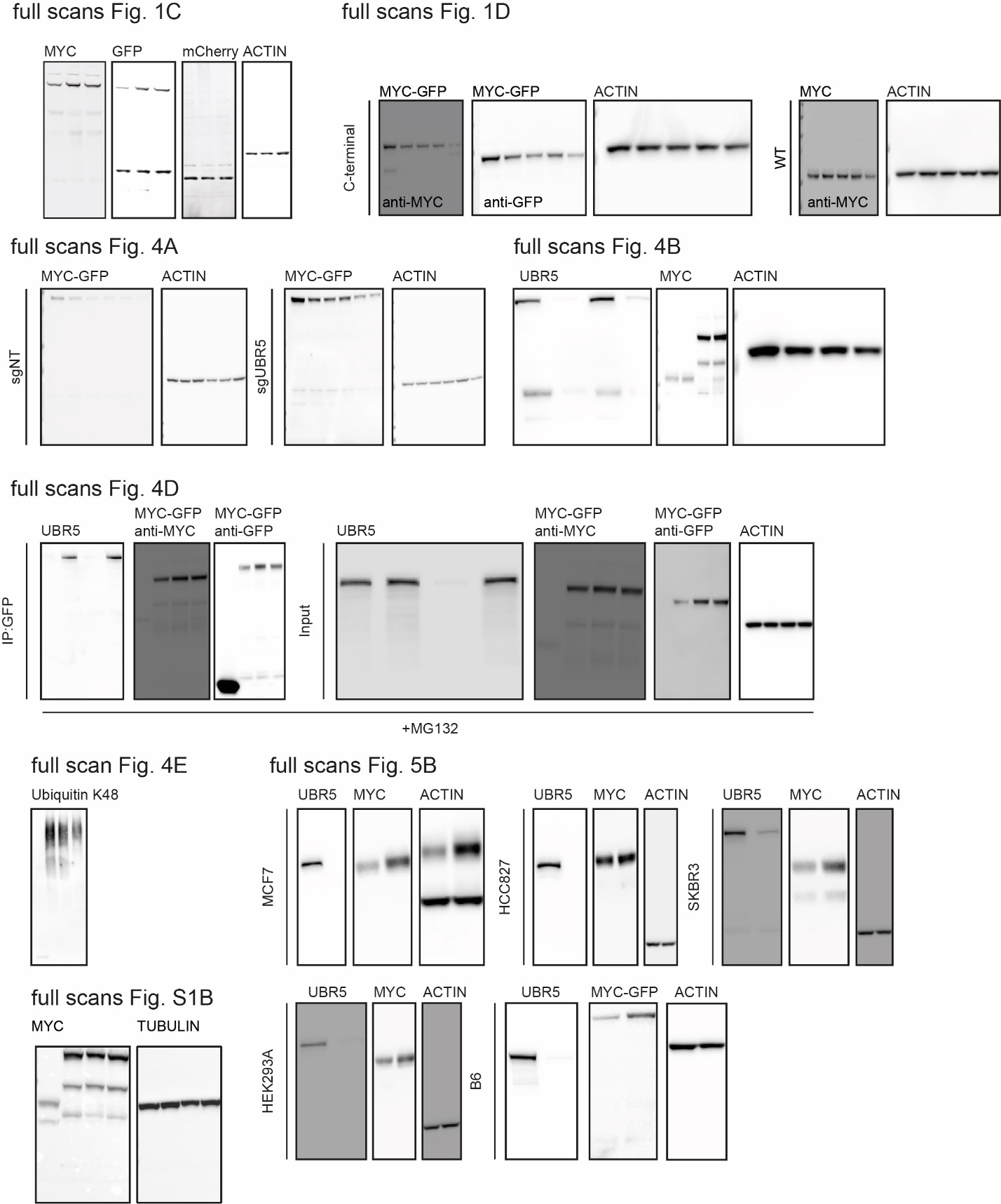


Supplementary figure 6: full scans of all western blots from figures 1C, 1D, 4A, 4B, 4D, 4E, 5B and supplementary figure 1B, western blots were made from SDS-PAGE mini-gels.

|  | Median (LogFC) [Average of med.logFC] | | | | RSA (stabilizer) [Average of LogP RSA score] | | | |
| --- | --- | --- | --- | --- | --- | --- | --- | --- |
|  |  |  |  |  |  |  |  |  |
|  | HIGH vs Unsorted | | LOW vs Unsorted | | HIGH vs Unsorted | | LOW vs Unsorted | |
| Gene Name | Day 7 | Day 14 | Day 7 | Day 14 | Day 7 | Day 14 | Day 7 | Day 14 |
| ABTB1 | 0.008 | 0.143 | -0.039 | 0.084 | -0.670 | -1.560 | -1.349 | -1.208 |
| ABTB2 | 0.008 | 0.086 | 0.080 | -0.050 | -0.408 | -1.023 | -0.769 | -0.600 |
| ADGRA3 | -0.080 | 0.058 | 0.041 | 0.074 | -0.430 | -2.322 | -0.680 | -1.253 |
| ADGRE2 | -0.097 | -0.145 | 0.571 | 0.304 | -0.778 | -0.075 | -8.760 | -4.425 |
| ADORA1 | -0.088 | -0.057 | 0.023 | 0.134 | -0.282 | -0.899 | -1.292 | -1.616 |
| AGER | -0.023 | -0.064 | 0.327 | 0.039 | -0.707 | -1.394 | -2.839 | -1.354 |
| AKTIP | -0.185 | -0.155 | 0.074 | 0.100 | -0.199 | -0.272 | -1.600 | -1.563 |
| AMBRA1 | 0.361 | 0.299 | -0.283 | -0.438 | -2.577 | -3.426 | -0.297 | -0.076 |
| AMFR | -0.296 | -0.063 | 0.038 | 0.006 | -0.076 | -0.374 | -0.853 | -0.670 |
| AMOT | 0.182 | -0.061 | -0.079 | -0.022 | -1.262 | -0.847 | -0.703 | -0.569 |
| ANAPC1 | 0.092 | -0.468 | 0.108 | 0.018 | -1.246 | -0.632 | -1.763 | -0.573 |
| ANAPC10 | 0.142 | 0.185 | 0.066 | 0.054 | -1.434 | -2.752 | -1.573 | -2.016 |
| ANAPC11 | 0.318 | 0.031 | -0.061 | -0.138 | -3.847 | -1.571 | -1.005 | -1.176 |
| ANAPC2 | -0.351 | -0.020 | -0.231 | 0.158 | -0.798 | -1.811 | -1.889 | -1.408 |
| ANAPC4 | 0.082 | 0.222 | 0.151 | -0.079 | -1.274 | -2.277 | -2.699 | -1.839 |
| ANAPC5 | -0.118 | 0.036 | 0.170 | 0.121 | -0.669 | -2.116 | -2.187 | -2.067 |
| ANAPC7 | -0.142 | -0.242 | 0.186 | 0.184 | -0.168 | -0.221 | -1.839 | -1.719 |
| ANKFY1 | 0.478 | -0.150 | -0.042 | 0.031 | -3.481 | -0.124 | -0.886 | -1.128 |
| ANKIB1 | 0.068 | -0.067 | 0.171 | 0.032 | -1.111 | -1.006 | -1.556 | -1.250 |
| ANKRD31 | -0.140 | -0.043 | 0.201 | 0.038 | -0.400 | -1.538 | -2.145 | -1.989 |
| AP4E1 | 0.198 | 0.162 | -0.179 | -0.133 | -1.384 | -1.843 | -0.657 | -0.683 |
| AQP5 | 0.061 | 0.033 | -0.045 | 0.097 | -0.803 | -0.795 | -0.925 | -1.032 |
| AREL1 | 0.370 | -0.063 | -0.060 | -0.030 | -3.467 | -0.404 | -0.321 | -0.724 |
| ARIH1 | 0.072 | -0.067 | 0.046 | 0.187 | -1.920 | -1.682 | -1.379 | -1.636 |
| ARIH2 | 0.044 | 0.158 | -0.237 | -0.074 | -1.181 | -1.595 | -0.132 | -0.637 |
| ARMC5 | 0.188 | 0.352 | -0.802 | -0.235 | -1.472 | -4.810 | -0.025 | -0.345 |
| ARPP21 | -0.072 | 0.015 | 0.030 | 0.009 | -0.674 | -1.499 | -0.682 | -0.769 |
| ASB1 | -0.033 | -0.085 | -0.179 | 0.013 | -0.347 | -0.733 | -1.009 | -0.659 |
| ASB10 | -0.110 | -0.010 | -0.165 | 0.044 | -0.552 | -0.572 | -0.186 | -1.030 |
| ASB11 | 0.099 | 0.148 | 0.060 | -0.049 | -1.572 | -1.325 | -1.100 | -0.717 |
| ASB12 | 0.134 | -0.072 | -0.259 | -0.039 | -3.473 | -0.389 | -0.304 | -0.691 |
| ASB13 | -0.057 | -0.235 | 0.050 | -0.055 | -1.001 | -0.152 | -2.065 | -0.512 |
| ASB14 | -0.099 | -0.029 | -0.096 | -0.064 | -0.139 | -1.167 | -0.416 | -0.605 |
| ASB15 | 0.132 | -0.071 | 0.135 | -0.003 | -1.208 | -0.270 | -1.668 | -0.612 |
| ASB16 | 0.282 | 0.267 | -0.209 | -0.157 | -2.471 | -2.654 | -0.648 | -1.606 |
| ASB17 | 0.033 | -0.027 | 0.103 | -0.055 | -0.546 | -0.468 | -1.380 | -0.788 |
| ASB18 | -0.230 | -0.078 | -0.068 | 0.017 | -0.082 | -0.522 | -0.636 | -0.806 |
| ASB2 | 0.081 | -0.060 | -0.059 | -0.073 | -1.066 | -1.192 | -0.678 | -1.080 |
| ASB3 | -0.158 | -0.062 | -0.066 | 0.035 | -0.386 | -1.176 | -0.543 | -1.398 |
| ASB4 | -0.412 | 0.043 | -0.120 | 0.202 | -0.158 | -0.682 | -0.707 | -2.483 |
| ASB5 | -0.092 | -0.087 | 0.026 | -0.017 | -0.856 | -1.303 | -0.550 | -2.024 |
| ASB6 | 0.087 | 0.045 | 0.188 | -0.059 | -0.754 | -0.818 | -1.341 | -0.239 |
| ASB7 | 0.072 | -0.134 | 0.000 | 0.103 | -1.174 | -0.539 | -0.976 | -1.012 |
| ASB8 | -0.101 | -0.006 | 0.088 | -0.007 | -0.188 | -0.388 | -1.607 | -1.039 |
| ASB9 | 0.325 | 0.083 | -0.002 | -0.063 | -4.123 | -0.691 | -0.885 | -0.388 |
| ASIC1 | -0.011 | 0.017 | 0.099 | 0.080 | -0.533 | -1.792 | -1.129 | -1.241 |
| ASNSD1 | -0.272 | -0.074 | 0.149 | 0.167 | -0.124 | -1.015 | -1.466 | -2.030 |
| ATG12 | 0.002 | -0.071 | -0.041 | 0.046 | -0.933 | -0.694 | -0.336 | -1.095 |
| ATG5 | -0.066 | -0.058 | 0.113 | 0.096 | -0.708 | -0.806 | -3.140 | -3.143 |
| ATG9B | -0.104 | 0.145 | 0.114 | 0.040 | -0.360 | -1.198 | -1.334 | -0.783 |
| ATP5PF | -0.285 | 0.079 | -0.215 | 0.174 | -0.698 | -0.807 | -0.429 | -1.617 |
| ATRX | -0.035 | -0.109 | -0.006 | 0.042 | -0.425 | -0.219 | -0.881 | -0.694 |
| ATXN3 | 0.121 | 0.063 | -0.043 | -0.039 | -2.833 | -1.926 | -0.648 | -1.232 |
| BABAM1 | -0.159 | -0.160 | 0.578 | 0.478 | -0.194 | -0.808 | -5.382 | -7.319 |
| BABAM2 | -0.149 | 0.013 | -0.004 | 0.048 | -0.222 | -0.918 | -0.984 | -1.300 |
| BACH1 | -0.321 | -0.023 | -0.100 | 0.064 | -0.183 | -1.041 | -1.026 | -1.986 |
| BACH2 | 0.161 | -0.074 | 0.204 | 0.082 | -1.383 | -0.748 | -1.682 | -2.094 |
| BAP1 | 0.438 | 0.476 | -0.961 | -0.722 | -3.155 | -4.291 | -0.070 | -0.042 |
| BARD1 | 0.150 | 0.439 | 0.128 | -0.344 | -1.120 | -3.496 | -1.521 | -0.950 |
| BCAP29 | -0.194 | -0.120 | 0.161 | 0.011 | -0.543 | -0.300 | -1.612 | -1.057 |
| BFAR | 0.037 | -0.017 | -0.111 | -0.026 | -1.528 | -0.736 | -0.648 | -0.822 |
| BIRC2 | -0.044 | -0.158 | 0.020 | -0.125 | -1.132 | -0.291 | -0.838 | -0.920 |
| BIRC3 | -0.093 | 0.029 | -0.090 | -0.195 | -0.428 | -1.451 | -0.378 | -0.317 |
| BIRC7 | 0.228 | 0.186 | -0.161 | 0.037 | -1.767 | -2.174 | -0.386 | -1.018 |
| BIRC8 | 0.070 | -0.061 | 0.110 | 0.113 | -1.325 | -0.414 | -1.651 | -1.898 |
| BMI1 | -0.305 | -0.083 | 0.224 | 0.082 | -0.289 | -0.639 | -2.043 | -1.409 |
| BRAP | -0.111 | 0.038 | -0.224 | -0.260 | -0.525 | -1.332 | -0.623 | -0.128 |
| BRCA1 | 0.311 | 0.002 | -0.118 | -0.132 | -3.038 | -1.573 | -0.824 | -0.111 |
| BRCC3 | 0.026 | 0.056 | 0.025 | 0.000 | -1.254 | -0.928 | -0.971 | -0.839 |
| BRWD1 | -0.221 | -0.195 | -0.007 | -0.017 | -0.138 | -0.127 | -1.223 | -1.179 |
| BRWD3 | 0.147 | 0.018 | 0.010 | 0.055 | -1.608 | -1.105 | -0.962 | -0.707 |
| BTBD1 | -0.068 | 0.025 | -0.141 | -0.151 | -1.025 | -0.782 | -0.493 | -0.421 |
| BTBD10 | -0.055 | 0.087 | 0.006 | -0.107 | -0.534 | -0.905 | -0.841 | -0.504 |
| BTBD11 | 0.079 | -0.068 | -0.017 | 0.047 | -0.896 | -0.225 | -1.206 | -0.983 |
| BTBD16 | -0.219 | -0.324 | 0.144 | 0.012 | -0.425 | -0.202 | -1.337 | -0.569 |
| BTBD17 | 0.167 | 0.011 | -0.152 | -0.129 | -1.428 | -1.115 | -1.525 | -1.408 |
| BTBD18 | 0.243 | 0.072 | -0.238 | -0.140 | -1.653 | -0.773 | -0.303 | -0.495 |
| BTBD19 | -0.163 | -0.024 | -0.102 | 0.207 | -0.313 | -0.822 | -0.377 | -2.088 |
| BTBD2 | -0.175 | -0.140 | 0.140 | 0.196 | -0.407 | -0.195 | -1.238 | -2.076 |
| BTBD3 | -0.083 | -0.123 | 0.105 | 0.139 | -0.874 | -0.218 | -1.414 | -2.113 |
| BTBD6 | 0.167 | 0.070 | 0.028 | -0.002 | -1.685 | -1.006 | -0.511 | -0.700 |
| BTBD7 | -0.009 | -0.052 | 0.177 | 0.127 | -1.344 | -0.647 | -2.173 | -1.723 |
| BTBD8 | -0.096 | -0.157 | 0.006 | 0.039 | -0.199 | -0.518 | -1.010 | -0.916 |
| BTBD9 | -0.234 | -0.166 | 0.305 | 0.216 | -0.152 | -0.119 | -2.710 | -2.296 |
| BTRC | 0.117 | 0.045 | 0.067 | -0.026 | -1.307 | -1.209 | -1.409 | -0.503 |
| BUB3 | -0.254 | -0.012 | -0.239 | -0.026 | -0.837 | -1.039 | -1.381 | -1.480 |
| C1orf68 | -0.284 | -0.026 | 0.045 | 0.033 | -0.379 | -0.243 | -1.148 | -1.383 |
| C2orf15 | -0.065 | -0.068 | 0.146 | 0.001 | -0.696 | -0.265 | -1.557 | -1.141 |
| CAND1 | 0.196 | 0.224 | 0.076 | -0.114 | -1.458 | -2.391 | -0.765 | -0.151 |
| CAND2 | -0.256 | 0.120 | -0.109 | 0.045 | -0.300 | -1.080 | -0.569 | -1.360 |
| CAVIN2 | -0.211 | -0.053 | -0.013 | -0.007 | -0.235 | -0.468 | -0.740 | -0.949 |
| CBL | 0.185 | 0.150 | 0.053 | 0.009 | -1.714 | -1.882 | -0.805 | -0.504 |
| CBLB | 0.022 | -0.011 | -0.102 | -0.068 | -0.964 | -0.343 | -0.713 | -0.510 |
| CBLC | -0.164 | -0.288 | 0.254 | 0.251 | -0.110 | -0.073 | -2.634 | -3.953 |
| CBLL1 | 0.167 | 0.052 | -0.198 | -0.152 | -1.215 | -1.036 | -0.243 | -0.454 |
| CBLL2 | 0.043 | 0.028 | -0.198 | -0.003 | -0.693 | -0.741 | -0.354 | -0.528 |
| CCDC9B | 0.237 | 0.228 | -0.163 | -0.156 | -4.419 | -4.040 | -0.482 | -0.473 |
| CCIN | -0.074 | -0.100 | 0.129 | 0.195 | -0.668 | -0.383 | -1.341 | -1.727 |
| CCNB1IP1 | 0.228 | -0.083 | -0.130 | -0.131 | -2.095 | -0.377 | -0.624 | -0.101 |
| CCNC | 0.754 | 0.844 | -0.997 | -0.962 | -6.645 | -7.765 | -0.060 | -0.251 |
| CCND1 | 0.082 | 0.077 | -0.033 | -0.127 | -1.173 | -1.624 | -0.524 | -0.813 |
| CCNE1 | -0.043 | 0.027 | 0.444 | 0.286 | -0.652 | -1.320 | -6.356 | -2.910 |
| CCNE2 | 0.033 | -0.028 | -0.129 | -0.131 | -1.597 | -0.832 | -0.633 | -0.482 |
| CCNF | -0.218 | -0.138 | -0.027 | -0.031 | -0.585 | -0.163 | -0.422 | -0.928 |
| CDC16 | -0.043 | 0.108 | 0.054 | -0.054 | -1.109 | -1.310 | -2.286 | -0.283 |
| CDC20 | -0.320 | -0.040 | -0.228 | -0.083 | -1.092 | -1.119 | -1.418 | -1.363 |
| CDC23 | -0.055 | 0.608 | 0.297 | 0.025 | -3.071 | -5.398 | -2.746 | -3.288 |
| CDC34 | -0.118 | -0.090 | 0.297 | 0.384 | -0.202 | -0.586 | -2.352 | -5.217 |
| CDCA7 | 0.069 | -0.070 | -0.012 | -0.054 | -1.208 | -0.627 | -0.865 | -0.873 |
| CDK2 | -0.099 | -0.043 | 0.099 | -0.025 | -0.519 | -0.350 | -1.886 | -0.616 |
| CDK4 | -0.032 | 0.125 | 0.001 | 0.114 | -1.222 | -1.023 | -0.442 | -2.214 |
| CDK6 | -0.085 | 0.032 | -0.076 | 0.021 | -0.317 | -2.231 | -0.517 | -1.932 |
| CDK9 | 0.604 | 0.336 | -0.654 | -0.060 | -7.045 | -3.412 | -0.019 | -1.015 |
| CDRT1 | -0.123 | -0.064 | -0.181 | -0.155 | -0.490 | -0.621 | -0.190 | -0.223 |
| CDX4 | 0.240 | 0.068 | 0.149 | -0.069 | -2.124 | -0.632 | -1.932 | -0.970 |
| CEP152 | 0.211 | 0.171 | 0.168 | -0.129 | -1.511 | -2.240 | -1.393 | -0.198 |
| CES1 | -0.068 | 0.040 | 0.109 | -0.006 | -0.410 | -0.580 | -1.759 | -1.614 |
| CGRRF1 | 0.150 | 0.005 | -0.054 | -0.059 | -2.548 | -1.656 | -0.251 | -1.097 |
| CHFR | -0.177 | -0.063 | 0.026 | 0.035 | -0.749 | -0.478 | -0.896 | -1.865 |
| CISH | -0.152 | 0.003 | -0.093 | 0.036 | -0.077 | -1.418 | -0.285 | -2.220 |
| CLCF1 | 0.085 | 0.217 | -0.093 | -0.157 | -0.976 | -2.071 | -0.638 | -0.521 |
| CMA1 | 0.405 | 0.017 | -0.126 | 0.033 | -3.887 | -0.656 | -0.402 | -0.566 |
| CMSS1 | 0.198 | 0.036 | 0.100 | -0.114 | -1.478 | -0.863 | -1.911 | -0.575 |
| CMTM6 | -0.074 | -0.085 | 0.075 | -0.062 | -0.672 | -0.532 | -1.055 | -0.488 |
| CNOT4 | -0.200 | -0.134 | -0.093 | -0.034 | -0.222 | -0.109 | -1.184 | -0.994 |
| CNTNAP2 | 0.169 | -0.063 | -0.037 | -0.043 | -1.652 | -0.789 | -0.694 | -1.087 |
| COP1 | -0.098 | 0.119 | 0.042 | -0.037 | -1.068 | -1.507 | -0.780 | -2.180 |
| COPS2 | 0.673 | 0.484 | -0.668 | -0.600 | -7.237 | -4.338 | -0.139 | -0.068 |
| COPS3 | 0.614 | 0.548 | -0.443 | -0.548 | -5.517 | -5.914 | -0.048 | -0.043 |
| COPS4 | 0.756 | 0.827 | -1.119 | -0.920 | -6.601 | -6.599 | -0.024 | -1.153 |
| COPS5 | 0.834 | 0.369 | -0.863 | -0.013 | -8.005 | -4.329 | -0.053 | -1.120 |
| COPS6 | 0.712 | 0.506 | -0.637 | 0.052 | -5.319 | -4.225 | -0.085 | -0.804 |
| COPS7A | 0.088 | 0.160 | 0.010 | -0.082 | -1.253 | -1.399 | -0.635 | -0.524 |
| COPS7B | -0.063 | 0.018 | -0.034 | 0.029 | -0.263 | -0.668 | -1.086 | -0.692 |
| COPS8 | 0.512 | 0.485 | -0.391 | -0.564 | -7.731 | -6.099 | -0.199 | -0.088 |
| CPSF1 | 0.512 | -0.299 | -0.081 | -0.458 | -3.824 | -1.935 | -0.577 | -0.629 |
| CRBN | -0.066 | -0.037 | 0.030 | 0.088 | -0.725 | -0.648 | -0.836 | -1.022 |
| CSHL1 | -0.092 | -0.121 | -0.319 | -0.107 | -0.426 | -0.224 | -0.293 | -0.224 |
| CUL1 | 1.315 | 0.827 | -0.693 | -0.177 | -10.580 | -6.619 | -0.032 | -0.585 |
| CUL2 | 0.026 | -0.015 | 0.191 | 0.017 | -0.543 | -0.351 | -2.104 | -2.049 |
| CUL3 | 0.216 | -0.089 | -0.023 | 0.237 | -1.407 | -0.357 | -0.761 | -2.149 |
| CUL4A | 0.082 | -0.031 | 0.154 | -0.112 | -0.800 | -1.250 | -2.628 | -0.469 |
| CUL4B | 0.166 | 0.259 | -0.094 | -0.280 | -1.868 | -3.548 | -0.153 | -0.204 |
| CUL5 | 0.213 | 0.132 | -0.172 | -0.145 | -2.389 | -1.689 | -0.227 | -0.231 |
| CUL7 | 0.111 | -0.108 | 0.079 | 0.062 | -0.759 | -0.123 | -1.166 | -0.988 |
| CUL9 | -0.284 | -0.108 | 0.195 | 0.058 | -0.523 | -0.285 | -1.370 | -0.660 |
| CXADR | -0.127 | -0.136 | 0.143 | 0.110 | -0.331 | -0.758 | -2.168 | -1.700 |
| CYLD | -0.202 | 0.030 | -0.043 | 0.141 | -0.335 | -1.618 | -0.485 | -2.254 |
| DCAF1 | 0.413 | -0.065 | -0.018 | 0.091 | -3.513 | -1.513 | -1.788 | -4.325 |
| DCAF10 | -0.141 | -0.083 | 0.006 | 0.001 | -0.197 | -0.434 | -1.799 | -1.062 |
| DCAF11 | 0.328 | 0.008 | -0.027 | -0.244 | -2.961 | -0.604 | -0.514 | -0.567 |
| DCAF12 | -0.170 | 0.024 | -0.123 | -0.170 | -0.417 | -0.569 | -0.255 | -0.165 |
| DCAF12L1 | 0.002 | 0.140 | -0.088 | -0.041 | -1.070 | -1.218 | -0.506 | -0.417 |
| DCAF12L2 | 0.020 | -0.150 | 0.129 | -0.436 | -0.812 | -0.269 | -1.244 | -0.140 |
| DCAF13 | 0.431 | 0.838 | -0.812 | -0.028 | -2.905 | -7.657 | -0.034 | -0.383 |
| DCAF15 | 0.016 | -0.176 | 0.650 | 0.206 | -0.815 | -0.023 | -7.424 | -2.545 |
| DCAF16 | 0.029 | -0.149 | -0.055 | 0.071 | -1.155 | -0.444 | -0.931 | -1.536 |
| DCAF17 | -0.089 | 0.083 | 0.127 | 0.035 | -0.770 | -1.112 | -1.096 | -0.986 |
| DCAF4 | 0.220 | 0.131 | -0.244 | -0.053 | -2.522 | -1.918 | -0.412 | -0.708 |
| DCAF4L1 | -0.094 | -0.043 | 0.036 | -0.081 | -0.845 | -1.359 | -1.210 | -0.595 |
| DCAF4L2 | 0.134 | -0.044 | -0.146 | 0.084 | -1.025 | -0.748 | -0.436 | -2.001 |
| DCAF5 | 0.291 | 0.149 | -0.217 | -0.130 | -2.991 | -2.476 | -0.537 | -0.372 |
| DCAF6 | -0.006 | -0.181 | 0.095 | -0.114 | -0.441 | -0.609 | -2.353 | -0.334 |
| DCAF7 | -0.367 | 0.103 | 0.148 | 0.436 | -0.264 | -0.897 | -1.317 | -5.607 |
| DCAF8 | -0.083 | 0.035 | 0.012 | -0.110 | -0.656 | -0.485 | -0.911 | -0.827 |
| DCAF8L1 | 0.279 | 0.087 | -0.086 | -0.055 | -3.704 | -2.481 | -0.465 | -0.940 |
| DCAF8L2 | 0.029 | 0.110 | 0.002 | 0.060 | -0.931 | -2.302 | -0.888 | -1.012 |
| DCST1 | 0.112 | -0.081 | 0.369 | 0.212 | -1.039 | -0.117 | -4.441 | -2.257 |
| DCUN1D1 | -0.074 | -0.182 | 0.075 | 0.190 | -0.246 | -0.393 | -1.113 | -4.926 |
| DDA1 | 0.076 | 0.183 | 0.188 | 0.262 | -1.201 | -1.507 | -1.856 | -3.946 |
| DDB1 | 0.279 | 0.090 | -0.292 | -0.211 | -2.075 | -1.264 | -0.437 | -0.197 |
| DDB2 | 0.284 | 0.016 | -0.105 | -0.190 | -2.102 | -1.874 | -0.212 | -0.145 |
| DET1 | -0.025 | 0.252 | -0.239 | -0.252 | -0.956 | -2.621 | -0.293 | -0.638 |
| DMWD | -0.070 | -0.228 | 0.562 | 0.314 | -0.896 | -0.052 | -6.664 | -4.611 |
| DPF1 | 0.121 | 0.032 | 0.503 | 0.382 | -0.890 | -1.099 | -5.449 | -6.211 |
| DTL | -0.426 | 0.021 | -0.744 | 0.229 | -0.467 | -3.377 | -1.163 | -5.573 |
| DTX1 | -0.178 | 0.064 | 0.153 | 0.280 | -0.220 | -0.768 | -1.268 | -3.228 |
| DTX2 | -0.017 | -0.019 | -0.054 | -0.134 | -0.439 | -0.564 | -0.606 | -0.483 |
| DTX3 | -0.195 | -0.121 | -0.076 | -0.165 | -0.251 | -0.311 | -1.687 | -0.198 |
| DTX3L | 0.224 | 0.103 | -0.188 | -0.027 | -1.503 | -2.009 | -0.663 | -0.915 |
| DTX4 | 0.080 | 0.060 | 0.105 | -0.219 | -0.733 | -1.193 | -1.302 | -0.348 |
| DUS2 | 0.008 | -0.031 | -0.136 | 0.013 | -0.699 | -1.143 | -0.580 | -1.289 |
| DZIP3 | -0.068 | 0.038 | 0.014 | -0.070 | -0.550 | -1.161 | -1.458 | -0.517 |
| EBF4 | -0.047 | 0.195 | -0.051 | -0.044 | -0.703 | -2.358 | -0.757 | -0.934 |
| ECT2L | -0.152 | -0.019 | -0.085 | 0.037 | -0.286 | -0.635 | -0.344 | -1.689 |
| EEF1A1 | -0.055 | 0.407 | 0.371 | -0.169 | -0.609 | -4.555 | -2.914 | -0.092 |
| EIF3F | 0.628 | 0.286 | -1.060 | -0.734 | -5.517 | -3.538 | -0.033 | -0.216 |
| EIF3H | 0.184 | 0.454 | -0.280 | -0.388 | -1.998 | -5.508 | -0.073 | -0.110 |
| EIF4A3 | 0.310 | -0.156 | -0.010 | -0.182 | -3.064 | -0.850 | -1.124 | -3.094 |
| ELOA | 1.158 | 1.062 | -0.906 | -0.911 | -9.231 | -10.472 | -0.013 | -0.008 |
| ELOB | 0.000 | 0.072 | -0.125 | -0.010 | -0.524 | -1.126 | -0.470 | -0.899 |
| ELOC | 0.098 | 0.169 | 0.108 | 0.000 | -1.018 | -2.065 | -3.348 | -3.507 |
| ENC1 | -0.001 | -0.123 | 0.211 | 0.147 | -0.339 | -0.521 | -1.676 | -3.139 |
| EPHB1 | -0.038 | -0.069 | -0.171 | 0.035 | -1.279 | -0.352 | -0.360 | -0.773 |
| EPX | 0.379 | 0.069 | -0.020 | -0.035 | -2.803 | -2.269 | -0.815 | -0.444 |
| ERCC8 | -0.215 | -0.127 | 0.312 | 0.066 | -0.258 | -0.389 | -3.484 | -1.702 |
| FAM19A1 | -0.072 | -0.022 | 0.014 | 0.020 | -0.459 | -0.685 | -0.717 | -0.552 |
| FAM83C | -0.137 | 0.074 | -0.108 | 0.003 | -0.869 | -1.374 | -0.508 | -0.617 |
| FANCL | -0.188 | -0.059 | -0.047 | 0.095 | -0.399 | -0.522 | -0.580 | -1.571 |
| FBH1 | -0.172 | -0.199 | 0.215 | 0.084 | -0.821 | -0.458 | -1.510 | -1.640 |
| FBXL12 | -0.119 | -0.385 | 0.571 | 0.384 | -0.625 | -0.096 | -5.065 | -5.573 |
| FBXL13 | 0.068 | -0.039 | 0.097 | 0.066 | -1.381 | -1.173 | -0.763 | -1.451 |
| FBXL14 | -0.114 | -0.100 | 0.042 | -0.075 | -0.692 | -0.169 | -1.503 | -0.284 |
| FBXL15 | -0.096 | 0.099 | 0.294 | -0.023 | -0.780 | -1.226 | -2.803 | -1.201 |
| FBXL16 | -0.126 | -0.180 | 0.094 | -0.183 | -0.269 | -0.098 | -0.898 | -0.260 |
| FBXL17 | 0.008 | -0.076 | -0.090 | -0.043 | -0.613 | -0.461 | -0.269 | -0.737 |
| FBXL18 | -0.073 | 0.278 | -0.302 | -0.251 | -0.307 | -2.136 | -0.199 | -0.641 |
| FBXL19 | -0.121 | -0.186 | 0.170 | -0.021 | -0.803 | -0.314 | -1.265 | -0.582 |
| FBXL2 | -0.179 | -0.100 | -0.089 | -0.021 | -0.228 | -0.456 | -0.690 | -0.767 |
| FBXL20 | 0.279 | 0.165 | -0.146 | -0.198 | -2.437 | -1.472 | -0.463 | -0.335 |
| FBXL21 | -0.038 | -0.178 | 0.124 | -0.054 | -0.596 | -0.276 | -1.564 | -0.668 |
| FBXL22 | 0.263 | 0.019 | -0.007 | -0.102 | -2.098 | -0.819 | -0.853 | -0.129 |
| FBXL3 | -0.179 | -0.040 | -0.079 | 0.029 | -0.242 | -0.495 | -0.593 | -1.754 |
| FBXL4 | -0.199 | -0.130 | 0.119 | 0.057 | -0.273 | -0.733 | -1.359 | -1.203 |
| FBXL5 | -0.065 | -0.033 | 0.025 | 0.103 | -0.518 | -0.601 | -0.620 | -1.035 |
| FBXL6 | -0.268 | 0.008 | 0.024 | -0.045 | -0.079 | -1.512 | -1.035 | -0.701 |
| FBXL7 | -0.039 | -0.235 | 0.043 | 0.059 | -0.449 | -0.712 | -1.922 | -1.293 |
| FBXL8 | -0.667 | -0.032 | 0.113 | 0.167 | -0.118 | -0.376 | -1.725 | -1.764 |
| FBXO10 | 0.096 | -0.121 | 0.007 | -0.006 | -0.990 | -0.409 | -1.189 | -0.664 |
| FBXO11 | -0.049 | 0.033 | -0.429 | -0.475 | -0.546 | -0.775 | -0.091 | -0.014 |
| FBXO15 | -0.068 | 0.069 | -0.121 | 0.064 | -0.868 | -0.977 | -0.881 | -1.220 |
| FBXO16 | 0.022 | 0.050 | -0.038 | -0.114 | -0.486 | -1.588 | -0.394 | -0.659 |
| FBXO17 | -0.226 | -0.169 | 0.436 | 0.323 | -0.121 | -0.389 | -4.173 | -6.997 |
| FBXO2 | -0.150 | -0.126 | 0.033 | -0.218 | -0.618 | -0.118 | -0.932 | -0.758 |
| FBXO21 | 0.016 | 0.024 | 0.009 | -0.087 | -0.730 | -1.793 | -0.747 | -0.388 |
| FBXO22 | 0.220 | -0.088 | 0.000 | -0.114 | -2.331 | -0.932 | -0.794 | -0.431 |
| FBXO24 | -0.215 | -0.002 | -0.064 | 0.076 | -0.340 | -0.466 | -0.268 | -1.227 |
| FBXO25 | -0.098 | -0.171 | 0.018 | -0.097 | -0.404 | -0.547 | -0.691 | -0.608 |
| FBXO27 | -0.274 | -0.188 | 0.381 | 0.215 | -0.169 | -0.819 | -3.230 | -3.107 |
| FBXO28 | -0.121 | -0.084 | 0.203 | -0.070 | -0.325 | -0.370 | -1.708 | -0.397 |
| FBXO3 | 0.149 | 0.069 | -0.098 | -0.205 | -2.229 | -1.172 | -0.839 | -0.134 |
| FBXO30 | 0.077 | 0.025 | -0.032 | 0.106 | -0.857 | -1.292 | -0.696 | -1.623 |
| FBXO31 | -0.054 | -0.106 | -0.078 | 0.169 | -0.374 | -0.158 | -1.088 | -2.346 |
| FBXO32 | 0.019 | 0.084 | -0.069 | -0.041 | -0.526 | -1.408 | -0.766 | -0.517 |
| FBXO33 | 0.200 | -0.005 | -0.114 | -0.211 | -1.507 | -0.687 | -0.322 | -0.543 |
| FBXO34 | 0.232 | 0.029 | -0.020 | -0.030 | -1.738 | -0.849 | -0.615 | -1.444 |
| FBXO36 | 0.038 | 0.129 | -0.009 | 0.069 | -0.809 | -1.291 | -0.538 | -1.099 |
| FBXO38 | -0.101 | -0.050 | -0.107 | -0.005 | -0.228 | -0.704 | -0.729 | -0.457 |
| FBXO39 | 0.077 | -0.057 | 0.042 | -0.009 | -0.828 | -0.720 | -0.744 | -0.979 |
| FBXO4 | -0.212 | -0.193 | 0.225 | 0.244 | -0.161 | -0.981 | -1.921 | -2.929 |
| FBXO40 | -0.061 | 0.138 | 0.003 | -0.030 | -0.774 | -1.542 | -1.421 | -0.896 |
| FBXO41 | -0.163 | -0.058 | 0.017 | 0.001 | -0.326 | -0.465 | -1.160 | -0.640 |
| FBXO42 | 0.168 | 0.221 | -0.159 | -0.228 | -1.198 | -2.863 | -0.449 | -0.096 |
| FBXO43 | 0.094 | 0.077 | -0.035 | -0.118 | -1.481 | -0.972 | -0.959 | -0.404 |
| FBXO44 | -0.027 | 0.129 | 0.052 | 0.220 | -0.365 | -1.547 | -1.075 | -2.255 |
| FBXO45 | -0.022 | -0.022 | 0.028 | -0.040 | -0.611 | -0.468 | -1.504 | -1.139 |
| FBXO46 | -0.181 | -0.207 | 0.536 | 0.364 | -0.125 | -0.259 | -6.926 | -4.744 |
| FBXO47 | -0.062 | 0.066 | -0.011 | -0.028 | -1.984 | -0.790 | -0.411 | -0.678 |
| FBXO48 | -0.105 | 0.052 | -0.066 | 0.103 | -0.670 | -0.660 | -0.507 | -1.058 |
| FBXO5 | -0.450 | 0.303 | -0.266 | 0.021 | -1.216 | -3.263 | -1.212 | -0.972 |
| FBXO6 | -0.098 | -0.079 | 0.063 | -0.028 | -0.245 | -0.910 | -0.924 | -2.357 |
| FBXO7 | -0.074 | -0.071 | -0.333 | -0.169 | -0.298 | -0.826 | -0.101 | -0.418 |
| FBXO8 | -0.060 | -0.001 | -0.071 | -0.082 | -0.610 | -0.651 | -1.294 | -0.716 |
| FBXO9 | -0.078 | -0.173 | 0.102 | 0.035 | -0.394 | -0.295 | -1.554 | -1.095 |
| FBXW10 | 0.102 | 0.060 | 0.055 | -0.015 | -1.065 | -1.445 | -0.888 | -0.697 |
| FBXW11 | -0.095 | -0.050 | 0.013 | -0.302 | -0.795 | -0.573 | -0.557 | -0.191 |
| FBXW12 | 0.050 | -0.098 | 0.005 | -0.083 | -1.155 | -0.584 | -1.758 | -0.458 |
| FBXW2 | 0.038 | -0.072 | 0.048 | 0.024 | -0.944 | -0.244 | -0.852 | -0.863 |
| FBXW4 | -0.148 | -0.032 | 0.308 | 0.038 | -0.281 | -0.402 | -3.181 | -1.427 |
| FBXW5 | 0.040 | 0.074 | 0.202 | 0.002 | -0.569 | -1.007 | -1.905 | -0.927 |
| FBXW7 | 1.535 | 1.422 | -1.278 | -1.534 | -24.853 | -27.840 | -0.018 | -0.002 |
| FBXW8 | -0.184 | -0.039 | 0.149 | 0.023 | -0.414 | -0.584 | -1.871 | -0.778 |
| FBXW9 | -0.201 | -0.107 | 0.487 | 0.442 | -0.070 | -0.359 | -4.398 | -4.390 |
| FSCN1 | 0.037 | -0.222 | -0.013 | -0.319 | -0.698 | -0.085 | -0.598 | -0.105 |
| FSIP1 | 0.093 | 0.102 | 0.078 | -0.123 | -1.160 | -1.331 | -1.133 | -0.288 |
| FZD3 | -0.143 | 0.033 | -0.039 | -0.049 | -0.789 | -2.084 | -0.797 | -1.031 |
| FZR1 | -0.176 | 0.000 | 0.143 | 0.022 | -0.155 | -0.642 | -1.872 | -1.453 |
| G3BP1 | 0.549 | 0.548 | -0.577 | -0.628 | -5.667 | -5.602 | -0.123 | -0.063 |
| G3BP2 | 0.114 | 0.072 | -0.131 | -0.053 | -0.849 | -1.032 | -0.568 | -0.370 |
| GABRR1 | -0.031 | -0.151 | 0.208 | 0.082 | -0.787 | -0.416 | -2.501 | -1.402 |
| GAN | -0.015 | -0.018 | 0.093 | 0.010 | -0.648 | -0.322 | -2.172 | -0.387 |
| GFP | -0.223 | 0.264 | -1.090 | -0.317 | -6.331 | -10.352 | -0.848 | -5.155 |
| GFRAL | -0.260 | -0.074 | 0.186 | 0.121 | -0.309 | -0.630 | -1.519 | -1.956 |
| GLT1D1 | -0.048 | -0.067 | -0.029 | -0.094 | -0.600 | -1.224 | -0.902 | -1.002 |
| GMCL1 | -0.038 | 0.058 | 0.005 | 0.007 | -0.995 | -0.651 | -1.000 | -0.690 |
| GPS1 | 0.772 | 0.196 | -0.788 | 0.013 | -7.875 | -1.817 | -0.508 | -1.706 |
| GTF2H2 | 0.040 | -0.176 | 0.437 | 0.081 | -0.662 | -1.161 | -4.489 | -0.782 |
| GYS2 | -0.118 | -0.184 | 0.055 | -0.095 | -0.251 | -0.230 | -1.873 | -0.298 |
| GZF1 | -0.006 | 0.025 | 0.044 | -0.066 | -1.045 | -2.011 | -1.224 | -0.481 |
| HACE1 | -0.151 | -0.075 | 0.206 | 0.108 | -0.964 | -0.309 | -2.310 | -1.034 |
| HECTD1 | 0.159 | -0.038 | 0.210 | -0.091 | -2.172 | -0.633 | -2.304 | -0.347 |
| HECTD2 | -0.269 | 0.053 | 0.107 | 0.165 | -0.308 | -1.150 | -1.573 | -2.520 |
| HECTD3 | -0.015 | 0.002 | 0.025 | 0.089 | -0.324 | -0.447 | -0.639 | -1.840 |
| HECTD4 | -0.165 | -0.060 | 0.078 | 0.225 | -0.088 | -0.498 | -1.384 | -2.818 |
| HECW1 | -0.043 | -0.016 | -0.058 | 0.113 | -0.597 | -0.444 | -0.984 | -1.739 |
| HECW2 | -0.026 | -0.046 | -0.205 | 0.049 | -0.416 | -0.380 | -0.599 | -1.151 |
| HERC1 | -0.021 | 0.129 | -0.041 | -0.165 | -1.151 | -1.439 | -0.534 | -0.278 |
| HERC2 | 0.004 | 0.222 | 0.011 | -0.221 | -1.079 | -2.719 | -0.801 | -0.298 |
| HERC3 | -0.017 | 0.018 | -0.140 | -0.125 | -0.775 | -0.773 | -0.426 | -0.604 |
| HERC4 | -0.093 | 0.012 | 0.062 | 0.018 | -1.733 | -0.880 | -1.397 | -0.715 |
| HERC5 | -0.014 | 0.031 | -0.045 | -0.203 | -1.018 | -0.924 | -0.430 | -0.539 |
| HERC6 | -0.105 | -0.098 | 0.045 | -0.057 | -0.786 | -0.361 | -0.536 | -0.737 |
| HIC1 | -0.125 | 0.033 | 0.068 | -0.014 | -0.544 | -1.291 | -1.409 | -1.066 |
| HIC2 | 0.036 | 0.052 | -0.167 | -0.139 | -1.507 | -0.911 | -0.279 | -0.208 |
| HLA-DQB2 | -0.068 | -0.021 | 0.128 | 0.100 | -0.474 | -0.837 | -0.928 | -2.323 |
| HLTF | 0.319 | 0.003 | -0.118 | 0.005 | -2.299 | -1.408 | -0.750 | -0.894 |
| HRASLS | -0.140 | 0.182 | 0.044 | 0.135 | -0.319 | -2.093 | -0.498 | -1.674 |
| HSPA9 | 0.063 | 0.214 | -0.380 | -0.160 | -0.938 | -2.860 | -0.055 | -1.597 |
| HUWE1 | 0.203 | 0.313 | 0.093 | 0.246 | -1.370 | -2.736 | -1.213 | -3.278 |
| IBTK | -0.211 | -0.124 | 0.046 | 0.058 | -0.158 | -0.297 | -1.377 | -0.830 |
| IFNA16 | 0.087 | -0.178 | 0.069 | -0.060 | -0.713 | -0.285 | -1.087 | -0.411 |
| IPP | -0.113 | -0.121 | 0.187 | -0.017 | -1.437 | -0.193 | -1.973 | -0.853 |
| IRF1 | -0.098 | -0.139 | 0.057 | 0.081 | -0.936 | -0.491 | -2.056 | -1.486 |
| IRF2 | -0.221 | -0.223 | 0.009 | -0.144 | -0.181 | -0.375 | -0.854 | -0.624 |
| IRF2BP1 | 0.042 | -0.003 | 0.234 | 0.319 | -1.226 | -0.838 | -2.705 | -3.448 |
| IRF2BPL | 0.157 | 0.106 | -0.227 | 0.068 | -1.038 | -0.977 | -0.313 | -1.423 |
| IRF3 | -0.046 | -0.227 | 0.254 | 0.327 | -0.304 | -0.555 | -5.745 | -5.423 |
| IRF4 | 0.300 | -0.130 | 0.080 | 0.056 | -2.133 | -0.222 | -1.716 | -1.917 |
| IRF5 | -0.112 | -0.150 | -0.023 | 0.053 | -1.112 | -0.355 | -0.524 | -0.902 |
| IRF6 | -0.020 | -0.071 | -0.033 | -0.102 | -0.493 | -0.253 | -1.023 | -0.461 |
| IRF7 | 0.043 | 0.203 | -0.110 | 0.017 | -1.098 | -2.427 | -0.923 | -0.721 |
| IRF8 | -0.240 | 0.022 | -0.142 | 0.124 | -0.247 | -0.693 | -1.002 | -2.003 |
| IRF9 | 0.229 | -0.002 | -0.245 | -0.098 | -2.506 | -0.646 | -0.188 | -0.744 |
| IRGM | -0.027 | -0.014 | 0.094 | 0.080 | -0.263 | -0.768 | -1.073 | -1.333 |
| ISM2 | 0.216 | -0.128 | 0.018 | -0.121 | -1.727 | -0.251 | -1.935 | -0.363 |
| ITCH | -0.094 | 0.036 | 0.057 | -0.091 | -0.385 | -1.124 | -0.639 | -0.513 |
| ITGA1 | -0.269 | -0.164 | 0.077 | 0.157 | -0.187 | -0.283 | -2.100 | -2.321 |
| IVNS1ABP | -0.202 | -0.012 | 0.189 | 0.141 | -0.317 | -0.433 | -1.431 | -2.369 |
| JOSD1 | -0.012 | -0.078 | -0.065 | -0.028 | -0.411 | -0.266 | -0.581 | -0.573 |
| JOSD2 | 0.109 | -0.144 | 0.377 | 0.127 | -0.914 | -0.489 | -2.910 | -1.498 |
| JPH4 | 0.374 | 0.185 | 0.017 | -0.011 | -2.918 | -2.193 | -1.745 | -1.818 |
| KBTBD11 | 0.160 | -0.018 | -0.118 | 0.046 | -1.007 | -0.466 | -0.368 | -0.704 |
| KBTBD12 | 0.112 | 0.197 | 0.050 | 0.025 | -1.413 | -1.946 | -0.742 | -0.960 |
| KBTBD13 | 0.067 | 0.133 | -0.073 | -0.136 | -0.931 | -2.066 | -0.745 | -0.723 |
| KBTBD2 | -0.800 | -0.487 | 0.428 | 0.359 | -0.043 | -0.039 | -3.479 | -3.721 |
| KBTBD3 | 0.174 | 0.113 | 0.091 | -0.174 | -1.337 | -2.785 | -1.049 | -0.400 |
| KBTBD4 | 0.038 | 0.035 | 0.003 | -0.079 | -0.934 | -1.386 | -0.878 | -0.179 |
| KBTBD6 | -0.283 | -0.087 | -0.011 | -0.050 | -0.162 | -0.456 | -0.353 | -0.773 |
| KBTBD7 | -0.097 | -0.114 | -0.335 | 0.050 | -0.428 | -0.480 | -0.170 | -0.742 |
| KBTBD8 | -0.024 | -0.154 | -0.049 | 0.024 | -0.459 | -0.471 | -0.642 | -0.899 |
| KCNA1 | 0.015 | -0.020 | 0.203 | 0.234 | -0.389 | -1.285 | -2.913 | -2.365 |
| KCNA10 | -0.120 | -0.126 | 0.294 | 0.173 | -0.456 | -0.382 | -2.935 | -1.712 |
| KCNA2 | -0.097 | -0.140 | 0.245 | 0.161 | -0.474 | -0.476 | -3.212 | -1.524 |
| KCNA3 | -0.213 | -0.116 | 0.189 | 0.088 | -0.535 | -0.556 | -1.495 | -2.205 |
| KCNA4 | 0.077 | 0.211 | 0.034 | -0.050 | -0.889 | -2.903 | -0.580 | -0.261 |
| KCNA5 | -0.016 | -0.090 | -0.033 | 0.060 | -0.624 | -0.362 | -0.936 | -1.043 |
| KCNA6 | -0.256 | -0.181 | -0.077 | 0.040 | -0.475 | -0.372 | -1.862 | -0.708 |
| KCNA7 | -0.039 | -0.044 | 0.503 | 0.245 | -0.967 | -0.353 | -5.770 | -3.222 |
| KCNB1 | 0.010 | -0.105 | 0.015 | -0.121 | -0.487 | -0.626 | -0.843 | -0.449 |
| KCNB2 | -0.091 | 0.081 | -0.013 | -0.133 | -0.922 | -1.656 | -1.034 | -0.429 |
| KCNC1 | 0.415 | 0.235 | 0.169 | 0.101 | -2.879 | -2.484 | -1.539 | -1.216 |
| KCNC2 | -0.042 | -0.106 | 0.073 | -0.018 | -0.795 | -0.081 | -0.852 | -0.591 |
| KCNC3 | 0.007 | -0.214 | 0.412 | 0.181 | -1.206 | -0.119 | -7.629 | -3.288 |
| KCNC4 | -0.427 | -0.134 | 0.311 | 0.079 | -0.666 | -0.370 | -2.184 | -1.300 |
| KCND1 | 0.330 | 0.035 | -0.128 | -0.078 | -2.198 | -0.804 | -0.278 | -1.208 |
| KCND2 | -0.004 | -0.095 | -0.159 | -0.043 | -0.512 | -0.779 | -0.460 | -0.563 |
| KCND3 | -0.186 | 0.004 | 0.255 | 0.132 | -0.526 | -0.429 | -3.254 | -1.687 |
| KCNG1 | -0.175 | 0.075 | 0.122 | 0.123 | -0.237 | -1.106 | -1.016 | -1.588 |
| KCNG3 | 0.063 | 0.009 | 0.058 | -0.024 | -1.514 | -0.393 | -0.918 | -0.751 |
| KCNRG | -0.026 | -0.041 | -0.004 | -0.012 | -1.206 | -0.408 | -0.654 | -0.782 |
| KCNS1 | -0.128 | 0.158 | -0.078 | -0.029 | -0.508 | -1.834 | -0.251 | -0.507 |
| KCNS2 | -0.021 | 0.099 | -0.092 | -0.066 | -0.616 | -1.900 | -0.376 | -0.495 |
| KCNS3 | -0.115 | -0.056 | 0.062 | 0.009 | -0.860 | -0.509 | -1.161 | -0.667 |
| KCNV1 | -0.001 | -0.082 | -0.103 | -0.072 | -0.820 | -0.438 | -0.750 | -0.267 |
| KCTD1 | 0.097 | -0.086 | -0.096 | -0.017 | -1.064 | -0.373 | -0.423 | -1.479 |
| KCTD10 | 0.020 | -0.112 | 0.131 | 0.090 | -0.414 | -0.165 | -1.088 | -3.211 |
| KCTD12 | -0.195 | -0.121 | -0.117 | -0.231 | -0.106 | -0.389 | -0.329 | -0.167 |
| KCTD13 | -0.001 | -0.125 | -0.126 | -0.007 | -0.693 | -0.260 | -0.367 | -0.482 |
| KCTD14 | 0.154 | 0.123 | -0.191 | -0.223 | -1.256 | -1.125 | -0.314 | -0.372 |
| KCTD15 | -0.422 | -0.044 | 0.610 | 0.214 | -0.178 | -0.197 | -6.998 | -2.762 |
| KCTD16 | -0.038 | -0.133 | -0.068 | -0.088 | -1.127 | -0.548 | -0.850 | -0.694 |
| KCTD17 | -0.099 | -0.161 | -0.103 | -0.032 | -0.415 | -0.819 | -0.394 | -0.476 |
| KCTD2 | 0.165 | 0.013 | -0.243 | -0.097 | -2.357 | -1.152 | -0.284 | -1.788 |
| KCTD20 | -0.110 | -0.217 | 0.053 | 0.157 | -0.229 | -0.272 | -1.086 | -1.507 |
| KCTD21 | 0.064 | 0.020 | -0.185 | -0.257 | -1.038 | -0.864 | -0.309 | -0.424 |
| KCTD3 | -0.041 | -0.040 | 0.081 | 0.106 | -0.948 | -0.932 | -0.878 | -2.331 |
| KCTD4 | -0.069 | -0.116 | -0.045 | -0.130 | -0.751 | -0.356 | -0.425 | -0.380 |
| KCTD5 | 0.235 | 0.064 | -0.077 | -0.171 | -2.012 | -1.225 | -1.086 | -0.652 |
| KCTD6 | 0.108 | 0.025 | 0.022 | -0.175 | -0.947 | -1.059 | -0.833 | -0.213 |
| KCTD7 | -0.053 | -0.185 | -0.109 | 0.025 | -0.645 | -0.143 | -0.453 | -0.731 |
| KCTD8 | -0.062 | -0.096 | 0.138 | -0.074 | -0.502 | -0.450 | -1.237 | -0.385 |
| KCTD9 | 0.227 | -0.028 | -0.183 | -0.079 | -1.760 | -1.103 | -0.350 | -0.629 |
| KDM2A | 0.125 | 0.178 | -0.529 | -0.258 | -1.763 | -1.909 | -0.113 | -0.207 |
| KDM2B | -0.325 | -0.187 | -0.075 | 0.025 | -0.112 | -0.160 | -0.462 | -0.538 |
| KEAP1 | -0.203 | 0.036 | 0.363 | 0.453 | -0.296 | -0.490 | -4.209 | -7.096 |
| KLHDC10 | 0.122 | 0.002 | -0.145 | -0.063 | -0.966 | -0.710 | -0.460 | -1.299 |
| KLHDC2 | 0.105 | 0.066 | -0.406 | -0.192 | -1.787 | -0.821 | -1.356 | -0.763 |
| KLHDC3 | 0.118 | -0.005 | 0.185 | 0.228 | -1.135 | -0.359 | -2.039 | -2.419 |
| KLHL1 | -0.083 | -0.132 | -0.118 | 0.044 | -0.387 | -0.352 | -0.472 | -0.590 |
| KLHL10 | 0.043 | 0.070 | -0.057 | -0.085 | -0.830 | -0.749 | -1.165 | -0.467 |
| KLHL11 | -0.064 | -0.001 | -0.062 | 0.079 | -0.419 | -0.780 | -1.084 | -1.510 |
| KLHL12 | -0.048 | -0.047 | 0.105 | 0.129 | -0.303 | -0.422 | -1.073 | -1.304 |
| KLHL13 | 0.227 | 0.004 | -0.019 | -0.161 | -1.925 | -1.042 | -1.568 | -0.425 |
| KLHL14 | -0.034 | 0.081 | 0.077 | 0.194 | -0.469 | -0.832 | -2.213 | -2.961 |
| KLHL15 | 0.213 | -0.135 | -0.162 | -0.278 | -1.407 | -0.200 | -0.378 | -0.120 |
| KLHL17 | -0.190 | 0.008 | 0.095 | 0.086 | -0.692 | -0.429 | -1.817 | -1.448 |
| KLHL18 | -0.279 | -0.084 | -0.041 | -0.040 | -0.615 | -0.327 | -0.673 | -0.766 |
| KLHL2 | -0.136 | -0.089 | -0.049 | -0.103 | -0.395 | -0.953 | -0.497 | -0.878 |
| KLHL20 | -0.238 | -0.188 | 0.341 | 0.284 | -0.273 | -0.350 | -4.022 | -4.168 |
| KLHL21 | -0.154 | -0.151 | 0.094 | -0.145 | -0.450 | -0.159 | -0.918 | -0.280 |
| KLHL22 | 0.057 | -0.004 | -0.108 | -0.200 | -1.754 | -1.227 | -0.554 | -0.395 |
| KLHL23 | -0.144 | -0.080 | 0.266 | -0.070 | -0.826 | -0.443 | -2.493 | -1.003 |
| KLHL24 | -0.108 | 0.032 | -0.072 | -0.084 | -0.608 | -0.823 | -0.781 | -0.758 |
| KLHL25 | 0.152 | 0.103 | -0.030 | -0.018 | -1.068 | -2.823 | -0.864 | -0.292 |
| KLHL26 | -0.452 | -0.076 | 0.735 | 0.589 | -0.166 | -0.288 | -7.793 | -6.365 |
| KLHL28 | 0.248 | 0.130 | -0.075 | 0.002 | -2.547 | -2.426 | -0.372 | -1.584 |
| KLHL29 | -0.096 | -0.013 | -0.150 | -0.042 | -0.486 | -0.627 | -0.250 | -1.141 |
| KLHL3 | -0.020 | 0.010 | -0.018 | 0.144 | -0.300 | -0.400 | -0.723 | -1.956 |
| KLHL30 | -0.166 | -0.127 | 0.005 | -0.037 | -0.271 | -0.641 | -0.423 | -0.844 |
| KLHL31 | -0.031 | 0.017 | 0.173 | 0.023 | -0.351 | -0.578 | -1.717 | -0.888 |
| KLHL32 | 0.065 | -0.049 | 0.117 | 0.117 | -0.781 | -1.154 | -3.875 | -3.603 |
| KLHL33 | 0.278 | 0.103 | -0.146 | -0.020 | -2.187 | -1.398 | -0.809 | -0.401 |
| KLHL34 | 0.273 | 0.127 | -0.039 | 0.096 | -2.451 | -1.231 | -0.393 | -0.977 |
| KLHL35 | 0.229 | 0.001 | -0.071 | -0.148 | -1.752 | -0.526 | -1.037 | -0.647 |
| KLHL36 | -0.074 | -0.030 | 0.181 | -0.024 | -0.542 | -0.549 | -2.013 | -1.341 |
| KLHL38 | -0.015 | 0.233 | -0.262 | -0.007 | -1.214 | -2.310 | -0.435 | -1.153 |
| KLHL4 | 0.181 | 0.070 | -0.016 | 0.001 | -2.605 | -0.902 | -1.230 | -0.632 |
| KLHL40 | 0.009 | 0.008 | -0.125 | -0.051 | -0.549 | -0.551 | -0.238 | -0.892 |
| KLHL41 | 0.095 | -0.056 | 0.026 | 0.046 | -0.953 | -0.519 | -1.559 | -0.656 |
| KLHL42 | -0.170 | 0.024 | 0.034 | 0.198 | -0.646 | -0.798 | -1.591 | -2.096 |
| KLHL5 | 0.061 | 0.051 | -0.004 | -0.030 | -0.640 | -1.653 | -0.979 | -0.579 |
| KLHL6 | -0.171 | -0.015 | -0.125 | -0.033 | -0.212 | -0.935 | -0.499 | -1.255 |
| KLHL7 | -0.031 | 0.061 | -0.065 | 0.123 | -1.044 | -1.570 | -1.681 | -1.605 |
| KLHL8 | 0.036 | -0.077 | -0.180 | -0.086 | -0.528 | -0.672 | -0.277 | -0.508 |
| KLHL9 | -0.079 | -0.023 | -0.044 | 0.047 | -0.995 | -0.414 | -0.942 | -1.673 |
| KRT8 | -0.091 | 0.321 | -0.101 | 0.090 | -0.806 | -2.861 | -0.231 | -4.093 |
| KRTAP19-7 | -0.170 | -0.218 | -0.325 | -0.010 | -1.015 | -0.575 | -0.245 | -0.817 |
| LAMA4 | -0.004 | -0.004 | 0.150 | 0.083 | -0.858 | -0.691 | -1.861 | -2.183 |
| LCN1 | 0.026 | -0.063 | -0.120 | -0.199 | -0.814 | -0.413 | -0.127 | -0.266 |
| LEP | 0.013 | -0.186 | 0.034 | 0.012 | -0.522 | -0.330 | -0.919 | -0.504 |
| LGALS3BP | 0.061 | 0.024 | -0.017 | 0.010 | -0.686 | -1.354 | -0.524 | -0.521 |
| LGALS9C | 0.325 | 0.371 | -0.061 | -0.052 | -1.885 | -2.820 | -0.209 | -0.292 |
| LMO7 | -0.113 | 0.056 | -0.126 | -0.054 | -0.887 | -0.628 | -0.396 | -0.734 |
| LNX1 | 0.160 | -0.005 | -0.057 | -0.080 | -1.509 | -0.738 | -0.418 | -0.577 |
| LNX2 | -0.147 | 0.014 | -0.029 | 0.017 | -0.098 | -1.368 | -0.508 | -1.567 |
| LONRF1 | -0.069 | -0.048 | -0.091 | 0.128 | -0.675 | -1.024 | -0.470 | -1.755 |
| LONRF2 | -0.035 | -0.029 | 0.165 | -0.059 | -0.325 | -0.346 | -2.029 | -0.832 |
| LONRF3 | -0.022 | 0.075 | -0.173 | -0.152 | -0.486 | -1.278 | -0.249 | -0.630 |
| LPO | 0.169 | 0.118 | -0.137 | -0.092 | -2.760 | -1.091 | -0.259 | -0.678 |
| LRRC14 | 0.065 | -0.045 | 0.029 | -0.172 | -0.598 | -0.787 | -0.855 | -1.275 |
| LRRC29 | -0.152 | -0.096 | 0.083 | 0.174 | -0.303 | -0.287 | -0.669 | -2.138 |
| LRRC42 | -0.057 | 0.017 | -0.012 | -0.148 | -0.640 | -0.773 | -0.641 | -0.141 |
| LRRC66 | 0.087 | -0.043 | 0.038 | -0.126 | -1.824 | -0.605 | -1.369 | -0.711 |
| LRSAM1 | -0.109 | -0.087 | -0.075 | -0.168 | -0.882 | -0.444 | -0.832 | -0.360 |
| LTN1 | -0.442 | -0.269 | 0.241 | 0.186 | -0.050 | -0.087 | -1.934 | -2.724 |
| LZTR1 | 0.008 | 0.091 | 0.074 | -0.040 | -0.673 | -1.116 | -0.600 | -0.761 |
| MAGEB4 | 0.410 | 0.007 | -0.286 | -0.028 | -2.847 | -0.845 | -0.956 | -0.805 |
| MALT1 | 0.022 | -0.061 | -0.073 | -0.088 | -0.748 | -0.431 | -0.889 | -1.269 |
| MAP1LC3C | -0.161 | 0.025 | 0.095 | 0.035 | -0.411 | -0.879 | -0.984 | -0.900 |
| MARCH1 | 0.088 | 0.030 | 0.062 | -0.038 | -1.517 | -1.146 | -1.312 | -1.024 |
| MARCH2 | -0.007 | -0.050 | 0.559 | 0.381 | -0.445 | -0.394 | -6.619 | -7.486 |
| MARCH3 | -0.118 | -0.116 | 0.185 | 0.006 | -0.917 | -0.383 | -1.531 | -1.074 |
| MARCH4 | -0.051 | -0.034 | 0.057 | -0.029 | -0.568 | -0.253 | -2.123 | -0.537 |
| MARCH5 | -0.081 | -0.117 | 0.177 | 0.087 | -0.164 | -0.373 | -1.958 | -1.685 |
| MARCH6 | -0.267 | -0.175 | -0.091 | 0.036 | -0.357 | -0.302 | -0.559 | -0.543 |
| MARCH7 | -0.121 | 0.066 | 0.063 | -0.017 | -0.819 | -0.820 | -0.717 | -1.221 |
| MARCH8 | 0.140 | 0.028 | 0.206 | 0.196 | -1.250 | -1.605 | -2.824 | -3.322 |
| MARCH9 | -0.022 | 0.078 | 0.257 | 0.036 | -0.737 | -1.184 | -1.950 | -0.856 |
| MARCH10 | 0.091 | 0.150 | 0.018 | -0.007 | -1.183 | -2.229 | -1.995 | -0.596 |
| MARCH11 | 0.009 | -0.258 | 0.071 | 0.102 | -1.461 | -1.550 | -0.859 | -3.064 |
| MARK2 | 0.268 | 0.089 | 0.023 | 0.103 | -1.979 | -0.746 | -0.805 | -1.141 |
| mCHERRY | 1.713 | 1.719 | -1.718 | -2.429 | -30.947 | -36.730 | -0.001 | -0.001 |
| MDM2 | -0.164 | -0.106 | 0.273 | 0.028 | -0.159 | -0.412 | -2.356 | -1.166 |
| MDM4 | -0.033 | -0.089 | 0.004 | 0.033 | -0.328 | -0.545 | -2.266 | -0.955 |
| ME1 | 0.010 | -0.107 | 0.008 | 0.103 | -0.409 | -0.467 | -1.631 | -1.708 |
| MED12L | -0.194 | 0.116 | -0.066 | -0.034 | -0.700 | -0.983 | -1.250 | -0.627 |
| MEX3A | -0.176 | -0.089 | 0.242 | 0.142 | -0.278 | -0.255 | -1.908 | -1.800 |
| MEX3B | 0.274 | -0.029 | 0.151 | -0.148 | -2.591 | -1.147 | -1.229 | -0.535 |
| MEX3C | -0.042 | -0.080 | 0.052 | 0.048 | -0.359 | -0.324 | -1.099 | -0.883 |
| MEX3D | -0.296 | -0.175 | 0.394 | 0.408 | -1.080 | -0.120 | -3.412 | -4.275 |
| MGRN1 | -0.053 | -0.189 | -0.298 | -0.065 | -0.294 | -0.460 | -0.194 | -0.457 |
| MIB1 | -0.308 | -0.210 | 0.063 | 0.186 | -0.040 | -0.167 | -1.243 | -2.011 |
| MIB2 | -0.184 | 0.015 | 0.052 | -0.038 | -0.191 | -0.526 | -0.957 | -1.538 |
| MICAL1 | -0.273 | 0.057 | 0.089 | 0.077 | -0.578 | -0.897 | -0.768 | -0.753 |
| MID1 | 0.195 | 0.043 | -0.098 | -0.010 | -1.871 | -0.630 | -0.425 | -1.132 |
| MID2 | -0.080 | -0.027 | -0.109 | -0.048 | -0.348 | -0.623 | -0.574 | -0.237 |
| MKRN1 | -0.135 | -0.190 | -0.130 | 0.066 | -0.283 | -0.225 | -2.888 | -1.699 |
| MKRN2 | -0.103 | -0.037 | 0.027 | -0.161 | -0.301 | -0.606 | -0.651 | -0.306 |
| MKRN3 | 0.190 | 0.266 | -0.035 | -0.080 | -1.651 | -2.727 | -0.877 | -0.652 |
| MNAT1 | -0.026 | 0.001 | 0.293 | 0.210 | -0.340 | -0.370 | -4.701 | -5.077 |
| MSI1 | -0.159 | -0.138 | 0.074 | 0.003 | -0.231 | -0.752 | -0.937 | -1.564 |
| MSL2 | -0.137 | -0.158 | 0.237 | 0.239 | -0.125 | -0.540 | -3.505 | -2.946 |
| MTRNR2L7 | 0.023 | 0.015 | 0.068 | 0.175 | -0.696 | -1.111 | -1.427 | -2.145 |
| MUL1 | -0.268 | -0.172 | 0.201 | -0.032 | -0.259 | -0.336 | -2.474 | -0.798 |
| MYC | -0.120 | 0.115 | -0.021 | 0.039 | -1.115 | -1.500 | -0.342 | -0.697 |
| MYCBP2 | -0.306 | -0.180 | -0.162 | -0.024 | -0.484 | -0.576 | -0.403 | -1.527 |
| MYLIP | 0.057 | 0.161 | 0.197 | 0.130 | -1.062 | -1.446 | -2.450 | -1.979 |
| MYNN | -0.098 | 0.002 | 0.036 | -0.058 | -0.968 | -1.034 | -1.057 | -0.518 |
| NACC1 | -0.280 | 0.062 | 0.533 | 0.398 | -0.237 | -1.144 | -7.559 | -6.206 |
| NACC2 | 0.158 | -0.054 | -0.096 | -0.103 | -1.520 | -0.175 | -0.770 | -0.199 |
| NAE1 | 1.342 | -0.138 | -0.785 | -0.240 | -10.805 | -0.564 | -0.021 | -0.579 |
| NDUFA4 | -0.039 | -0.070 | 0.069 | 0.096 | -0.563 | -0.308 | -0.879 | -2.092 |
| NEDD4 | 0.215 | 0.005 | -0.108 | -0.304 | -1.753 | -0.765 | -1.373 | -0.450 |
| NEDD4L | -0.104 | -0.078 | 0.048 | 0.036 | -0.358 | -0.424 | -0.646 | -1.147 |
| NEDD8 | 1.721 | 0.593 | -1.471 | -0.045 | -14.497 | -6.030 | -0.012 | -2.698 |
| NEURL1 | -0.121 | -0.187 | 0.235 | 0.055 | -0.687 | -0.507 | -2.169 | -1.040 |
| NEURL1B | -0.150 | -0.033 | -0.009 | -0.051 | -0.253 | -0.438 | -0.566 | -0.608 |
| NEURL2 | 0.002 | 0.072 | -0.131 | 0.023 | -0.393 | -2.306 | -0.750 | -1.576 |
| NEURL3 | -0.126 | -0.053 | 0.261 | 0.047 | -0.135 | -0.290 | -2.783 | -0.804 |
| NEXMIF | 0.099 | 0.127 | -0.227 | -0.100 | -1.255 | -1.068 | -0.194 | -0.493 |
| NFATC4 | 0.129 | -0.121 | -0.064 | -0.041 | -1.107 | -0.531 | -0.205 | -0.206 |
| NFX1 | -0.141 | -0.007 | 0.115 | -0.079 | -0.332 | -1.612 | -1.486 | -0.499 |
| NFXL1 | 0.000 | 0.043 | -0.003 | -0.125 | -1.292 | -1.092 | -0.710 | -0.291 |
| NHLRC1 | -0.043 | 0.107 | -0.137 | 0.058 | -0.446 | -1.162 | -0.686 | -1.074 |
| NKAIN3 | -0.052 | 0.096 | -0.162 | -0.017 | -0.691 | -1.427 | -0.414 | -0.961 |
| NOSIP | 0.048 | -0.205 | 0.258 | -0.009 | -0.793 | -0.201 | -2.709 | -0.546 |
| NSD2 | 0.107 | 0.053 | -0.117 | -0.044 | -2.309 | -1.841 | -0.619 | -0.997 |
| NSMCE1 | -0.074 | 0.047 | -0.044 | 0.093 | -0.421 | -1.659 | -1.417 | -1.454 |
| OAT | 0.490 | 0.037 | -0.085 | 0.044 | -4.767 | -0.946 | -1.199 | -0.840 |
| OR10A4 | 0.051 | 0.040 | 0.104 | -0.164 | -1.187 | -0.836 | -0.941 | -0.566 |
| OR10S1 | -0.131 | 0.173 | 0.009 | -0.067 | -0.427 | -1.936 | -0.687 | -0.385 |
| OR10X1 | -0.065 | 0.022 | -0.015 | 0.066 | -0.208 | -1.069 | -2.010 | -1.068 |
| OR10Z1 | -0.160 | 0.086 | 0.061 | 0.210 | -0.415 | -0.960 | -2.195 | -2.672 |
| OR13C8 | -0.026 | -0.116 | -0.025 | 0.055 | -0.750 | -1.153 | -0.811 | -2.397 |
| OR13H1 | 0.228 | 0.108 | -0.007 | -0.037 | -2.722 | -1.469 | -0.983 | -1.403 |
| OR13J1 | 0.094 | 0.027 | -0.060 | -0.042 | -0.971 | -1.125 | -1.091 | -0.377 |
| OR1F1 | 0.131 | 0.017 | -0.053 | 0.054 | -1.472 | -0.567 | -0.239 | -1.236 |
| OR2AG2 | 0.111 | 0.112 | -0.020 | -0.029 | -2.683 | -1.945 | -0.380 | -0.607 |
| OR2C1 | 0.040 | -0.001 | -0.024 | 0.082 | -0.566 | -1.104 | -0.620 | -0.812 |
| OR2W1 | 0.109 | -0.142 | 0.036 | 0.013 | -1.309 | -0.114 | -0.885 | -1.518 |
| OR3A1 | -0.003 | -0.097 | 0.003 | -0.136 | -0.727 | -0.869 | -0.958 | -0.108 |
| OR3A2 | -0.126 | -0.033 | 0.084 | -0.141 | -0.277 | -0.482 | -0.818 | -0.269 |
| OR4C16 | 0.136 | 0.138 | 0.026 | -0.079 | -2.021 | -2.018 | -0.609 | -0.377 |
| OR4P4 | 0.106 | 0.144 | -0.131 | -0.243 | -1.089 | -1.602 | -0.638 | -0.403 |
| OR4X1 | 0.165 | 0.175 | -0.214 | -0.067 | -1.073 | -2.264 | -0.738 | -0.294 |
| OR52B2 | -0.038 | 0.003 | -0.112 | -0.106 | -0.820 | -0.925 | -0.684 | -0.163 |
| OR52B6 | -0.081 | -0.001 | -0.014 | -0.113 | -0.405 | -1.876 | -0.626 | -0.374 |
| OR52D1 | 0.105 | 0.066 | -0.072 | -0.045 | -2.776 | -2.158 | -0.205 | -0.599 |
| OR52J3 | 0.176 | 0.124 | 0.125 | -0.107 | -1.529 | -2.150 | -1.366 | -0.777 |
| OR52N1 | 0.079 | 0.034 | -0.080 | -0.018 | -0.858 | -0.816 | -0.874 | -0.601 |
| OR5C1 | 0.034 | 0.123 | 0.036 | -0.097 | -1.865 | -1.121 | -1.154 | -0.577 |
| OR5R1 | 0.089 | 0.114 | -0.171 | -0.179 | -0.768 | -1.061 | -0.354 | -0.442 |
| OR6C6 | -0.140 | -0.036 | -0.036 | -0.013 | -0.790 | -0.330 | -0.798 | -0.593 |
| OR6K2 | -0.069 | 0.104 | 0.121 | 0.177 | -0.792 | -0.814 | -1.226 | -2.607 |
| OR6N1 | -0.064 | -0.102 | 0.147 | 0.065 | -1.022 | -0.914 | -1.466 | -1.796 |
| OR6X1 | 0.139 | 0.032 | 0.012 | 0.027 | -1.671 | -0.556 | -0.521 | -0.507 |
| OR7D2 | -0.090 | -0.068 | 0.529 | 0.484 | -0.503 | -0.320 | -4.298 | -5.612 |
| OR7G3 | -0.026 | -0.112 | 0.471 | 0.275 | -0.569 | -0.565 | -4.544 | -3.687 |
| OR9A2 | -0.110 | -0.055 | -0.045 | -0.046 | -0.262 | -1.113 | -0.811 | -1.336 |
| OSBPL10 | -0.001 | -0.068 | -0.050 | 0.055 | -0.754 | -0.760 | -0.869 | -1.483 |
| OSBPL9 | -0.103 | -0.143 | 0.261 | -0.026 | -0.182 | -0.208 | -3.485 | -0.530 |
| OSM | -0.011 | 0.070 | -0.263 | -0.129 | -0.542 | -1.756 | -0.505 | -0.255 |
| OSTM1 | -0.081 | -0.098 | 0.044 | 0.081 | -0.648 | -0.330 | -1.055 | -1.897 |
| OTUB1 | 0.198 | 0.171 | -0.335 | -0.439 | -1.213 | -1.897 | -0.059 | -0.150 |
| OTUB2 | 0.224 | 0.086 | 0.053 | 0.052 | -1.731 | -1.255 | -1.621 | -1.344 |
| OTUD1 | -0.297 | -0.214 | 0.209 | 0.038 | -0.556 | -0.158 | -1.393 | -2.114 |
| OTUD4 | 0.190 | 0.041 | -0.183 | -0.101 | -1.757 | -1.721 | -0.153 | -0.421 |
| OTUD5 | 1.617 | 1.820 | -1.346 | -1.663 | -18.607 | -20.059 | -0.009 | -0.003 |
| OTUD6B | -0.046 | 0.218 | -0.092 | -0.211 | -1.953 | -2.683 | -0.625 | -0.292 |
| OTUD7A | 0.055 | -0.017 | 0.110 | -0.240 | -0.787 | -1.464 | -0.943 | -0.228 |
| OTUD7B | -0.193 | -0.071 | 0.408 | 0.128 | -0.271 | -0.275 | -3.063 | -2.016 |
| P2RX4 | 0.106 | 0.015 | 0.333 | 0.137 | -0.964 | -0.904 | -2.732 | -1.837 |
| PAN2 | -0.264 | -0.147 | 0.173 | -0.046 | -0.074 | -0.168 | -2.028 | -0.688 |
| PARP11 | -0.068 | 0.072 | -0.034 | 0.098 | -0.651 | -0.949 | -0.400 | -3.380 |
| PATZ1 | 0.268 | 0.216 | -0.304 | -0.127 | -1.776 | -3.011 | -0.267 | -0.778 |
| PAX1 | -0.144 | 0.020 | 0.009 | -0.062 | -1.003 | -0.479 | -0.454 | -0.215 |
| PCDHB9 | -0.059 | -0.001 | 0.132 | 0.071 | -0.471 | -0.680 | -1.053 | -1.104 |
| PCGF1 | -0.092 | 0.065 | 0.246 | 0.171 | -0.430 | -0.677 | -2.177 | -3.351 |
| PCGF2 | 0.281 | 0.101 | -0.095 | -0.014 | -2.622 | -1.811 | -0.525 | -1.442 |
| PCGF3 | -0.162 | -0.090 | -0.043 | -0.022 | -0.319 | -0.107 | -0.618 | -0.651 |
| PCGF5 | -0.130 | -0.122 | 0.081 | -0.065 | -0.368 | -0.176 | -1.293 | -0.506 |
| PCGF6 | -0.025 | -0.153 | 0.219 | 0.340 | -0.842 | -0.508 | -1.865 | -4.328 |
| PCP2 | 0.000 | -0.264 | 0.543 | 0.180 | -1.006 | -0.142 | -5.361 | -3.295 |
| PDZRN3 | 0.268 | -0.134 | -0.031 | -0.111 | -2.051 | -0.773 | -0.644 | -0.719 |
| PDZRN4 | -0.246 | 0.013 | 0.098 | 0.128 | -0.190 | -1.040 | -1.250 | -2.123 |
| PELI1 | -0.009 | 0.059 | 0.032 | 0.039 | -0.369 | -0.619 | -1.210 | -0.681 |
| PELI2 | 0.230 | 0.157 | 0.062 | 0.024 | -2.581 | -1.481 | -0.591 | -0.783 |
| PELI3 | 0.002 | -0.022 | -0.144 | -0.090 | -2.184 | -0.607 | -0.318 | -0.745 |
| PEX10 | -0.089 | -0.003 | 0.085 | 0.017 | -0.216 | -0.761 | -2.763 | -1.619 |
| PEX12 | -0.034 | 0.083 | -0.107 | 0.004 | -1.434 | -1.279 | -0.728 | -0.491 |
| PEX2 | 0.030 | -0.002 | 0.056 | -0.023 | -1.391 | -1.155 | -0.631 | -0.789 |
| PF4V1 | 0.008 | -0.051 | -0.141 | -0.035 | -1.178 | -0.333 | -0.415 | -0.645 |
| PHF5A | -0.058 | 0.000 | -0.072 | -0.103 | -0.808 | -2.188 | -2.308 | -2.432 |
| PHF7 | -0.198 | -0.116 | 0.019 | -0.082 | -0.120 | -0.584 | -1.279 | -0.747 |
| PHIP | -0.196 | -0.073 | 0.294 | 0.126 | -0.365 | -0.458 | -2.499 | -2.601 |
| PHLPP1 | -0.102 | -0.060 | 0.074 | -0.018 | -0.649 | -0.469 | -1.067 | -0.702 |
| PHLPP2 | -0.208 | -0.038 | 0.105 | 0.076 | -0.126 | -0.366 | -0.771 | -0.949 |
| PHRF1 | 0.005 | 0.081 | -0.062 | -0.110 | -0.533 | -0.956 | -0.421 | -0.419 |
| PIAS1 | -0.158 | 0.180 | -0.112 | -0.122 | -0.380 | -3.038 | -0.756 | -0.531 |
| PIAS2 | -0.023 | 0.167 | -0.065 | 0.057 | -0.768 | -1.574 | -0.462 | -1.408 |
| PIAS3 | 0.006 | -0.084 | 0.271 | -0.006 | -0.471 | -0.189 | -2.244 | -1.259 |
| PIAS4 | -0.019 | 0.226 | 0.176 | 0.317 | -0.675 | -1.780 | -3.437 | -4.177 |
| PJA1 | 0.034 | 0.015 | -0.171 | -0.036 | -0.487 | -1.042 | -0.404 | -0.586 |
| PJA2 | -0.066 | 0.075 | 0.092 | -0.075 | -0.950 | -0.805 | -1.214 | -0.671 |
| PLAUR | -0.138 | -0.082 | 0.364 | 0.238 | -0.490 | -0.294 | -3.866 | -3.583 |
| PLD3 | -0.165 | -0.263 | 0.588 | 0.161 | -0.345 | -0.131 | -5.136 | -2.299 |
| PLK1 | 0.109 | 0.342 | 0.148 | 0.026 | -4.339 | -2.108 | -1.965 | -1.790 |
| PLSCR1 | -0.017 | 0.006 | -0.028 | -0.090 | -0.462 | -0.593 | -1.836 | -0.409 |
| PML | 0.092 | 0.177 | -0.208 | -0.039 | -1.099 | -1.989 | -0.209 | -0.571 |
| POLR2A | 0.528 | -0.226 | 0.228 | -0.018 | -3.736 | -3.064 | -2.406 | -0.750 |
| POMP | 0.382 | 0.179 | -0.381 | -0.267 | -3.824 | -1.537 | -0.029 | -0.385 |
| PPIL2 | -0.001 | 0.526 | 1.054 | -0.009 | -0.389 | -4.530 | -12.574 | -1.252 |
| PRAC1 | -0.174 | 0.066 | -0.107 | -0.100 | -0.489 | -1.822 | -0.947 | -0.252 |
| PRKN | -0.110 | 0.024 | 0.091 | 0.034 | -0.690 | -0.577 | -0.877 | -0.804 |
| PROP1 | -0.251 | -0.101 | -0.050 | 0.041 | -0.259 | -0.474 | -0.228 | -0.975 |
| PRPF19 | 0.059 | 0.033 | -0.236 | 0.061 | -1.136 | -1.179 | -0.409 | -1.295 |
| PRPF4 | -0.653 | -0.135 | 0.212 | 0.326 | -0.097 | -0.674 | -2.041 | -4.329 |
| PRSS22 | -0.079 | 0.037 | -0.115 | 0.026 | -0.844 | -1.180 | -0.665 | -2.507 |
| PRSS56 | -0.164 | -0.095 | 0.202 | -0.025 | -0.864 | -0.521 | -1.748 | -0.598 |
| PSMA1 | 0.542 | 0.327 | -0.130 | -0.420 | -4.891 | -3.986 | -1.850 | -0.035 |
| PSMA2 | 0.579 | 0.000 | -0.729 | 0.047 | -6.534 | -3.124 | -1.158 | -1.130 |
| PSMA3 | 0.436 | 0.456 | -1.080 | -0.089 | -3.680 | -3.770 | -0.049 | -3.148 |
| PSMA4 | 0.578 | 0.546 | -0.844 | -0.481 | -4.740 | -4.648 | -2.111 | -0.836 |
| PSMA5 | 0.814 | 0.582 | -0.510 | 0.002 | -5.375 | -4.447 | -0.834 | -1.143 |
| PSMA6 | 0.717 | 0.033 | -0.527 | -0.396 | -5.774 | -0.857 | -0.104 | -0.892 |
| PSMA7 | 0.979 | -0.055 | -0.291 | -0.058 | -7.265 | -1.410 | -0.056 | -0.612 |
| PSMB3 | 0.638 | 0.263 | -0.423 | -0.076 | -6.080 | -2.500 | -2.372 | -1.356 |
| PSMB4 | 0.050 | -0.004 | -0.391 | -0.464 | -2.325 | -2.004 | -1.115 | -2.462 |
| PSMB5 | 0.105 | 0.361 | -1.038 | -0.038 | -3.935 | -3.785 | -2.966 | -2.866 |
| PSMB6 | 0.820 | 0.656 | -0.837 | 0.087 | -7.420 | -5.343 | -0.074 | -2.220 |
| PSMC1 | 0.732 | 0.014 | -0.339 | -0.047 | -5.952 | -1.748 | -0.209 | -0.801 |
| PSMD14 | 0.226 | 0.332 | -0.701 | -0.378 | -2.138 | -2.551 | -0.045 | -1.691 |
| PSMD7 | 0.160 | 0.247 | -0.399 | -0.176 | -1.637 | -2.279 | -0.078 | -0.071 |
| PSMF1 | 0.002 | 0.126 | -0.017 | -0.020 | -0.964 | -1.031 | -0.315 | -0.531 |
| PSMG1 | -0.083 | 0.474 | 0.023 | -0.295 | -0.321 | -4.210 | -0.978 | -0.796 |
| PSMG3 | 0.525 | 0.065 | -0.542 | -0.282 | -4.712 | -1.151 | -0.057 | -1.224 |
| PTGER4 | -0.275 | -0.177 | 0.180 | 0.222 | -0.242 | -0.064 | -1.437 | -2.349 |
| PTOV1 | 0.105 | -0.122 | 0.387 | 0.338 | -1.774 | -0.401 | -3.902 | -3.727 |
| RAB40A | 0.262 | 0.288 | -0.158 | -0.026 | -1.838 | -2.218 | -0.407 | -0.576 |
| RAB40B | -0.051 | -0.005 | 0.082 | -0.135 | -0.384 | -0.765 | -0.882 | -0.126 |
| RAB40C | -0.010 | 0.132 | -0.235 | -0.041 | -1.524 | -1.668 | -0.365 | -0.566 |
| RABGAP1L | -0.091 | 0.051 | 0.141 | 0.086 | -0.981 | -1.419 | -3.038 | -3.178 |
| RABGEF1 | -0.135 | 0.304 | 0.188 | 0.016 | -0.512 | -2.355 | -1.236 | -0.459 |
| RAD18 | 0.038 | 0.032 | 0.007 | -0.037 | -1.026 | -1.044 | -0.575 | -1.008 |
| RAE1 | 0.225 | 0.052 | 0.277 | 0.008 | -2.397 | -1.557 | -2.083 | -1.375 |
| RAG1 | 0.060 | 0.058 | -0.176 | -0.135 | -1.073 | -2.058 | -0.436 | -0.281 |
| RAN | -0.803 | -0.070 | -0.733 | -0.109 | -1.333 | -0.199 | -0.197 | -0.232 |
| RAPSN | 0.245 | 0.053 | -0.118 | -0.232 | -1.738 | -0.651 | -0.438 | -0.263 |
| RASGRF1 | -0.123 | 0.176 | -0.113 | -0.048 | -0.365 | -4.344 | -0.256 | -0.666 |
| RBBP6 | 1.123 | 0.733 | -1.068 | -0.504 | -16.242 | -12.124 | -0.006 | -0.023 |
| RBCK1 | 0.036 | -0.028 | 0.029 | -0.066 | -0.985 | -0.490 | -1.450 | -0.623 |
| RBX1 | 0.873 | 0.481 | -0.697 | -0.424 | -7.250 | -3.841 | -0.014 | -0.938 |
| RC3H1 | -0.219 | -0.017 | 0.176 | 0.040 | -0.742 | -0.683 | -3.287 | -1.094 |
| RC3H2 | -0.054 | 0.125 | -0.060 | -0.003 | -0.547 | -1.610 | -0.251 | -0.559 |
| RCBTB1 | 0.088 | 0.008 | 0.090 | -0.090 | -2.054 | -0.805 | -1.903 | -0.586 |
| RCBTB2 | -0.065 | -0.074 | -0.024 | 0.004 | -1.141 | -0.500 | -0.483 | -0.918 |
| RCHY1 | 0.138 | 0.076 | 0.003 | -0.050 | -1.286 | -1.904 | -0.746 | -1.043 |
| RFFL | -0.120 | 0.048 | -0.007 | -0.041 | -1.256 | -2.172 | -0.936 | -0.670 |
| RFPL1 | -0.018 | 0.250 | -0.152 | -0.111 | -0.399 | -2.300 | -0.312 | -0.536 |
| RFPL2 | -0.039 | 0.245 | 0.042 | 0.095 | -0.298 | -2.796 | -0.899 | -0.893 |
| RFPL3 | -0.070 | -0.069 | -0.262 | -0.203 | -0.652 | -0.302 | -0.162 | -0.411 |
| RFPL4A | -0.007 | 0.018 | 0.512 | 0.353 | -0.731 | -0.698 | -6.848 | -6.910 |
| RFPL4B | -0.188 | -0.095 | 0.017 | 0.130 | -0.561 | -0.706 | -0.437 | -1.619 |
| RFWD3 | -0.138 | -0.232 | 0.152 | -0.015 | -0.532 | -0.324 | -1.171 | -0.899 |
| RGS7 | -0.126 | -0.080 | 0.287 | 0.115 | -0.420 | -0.336 | -2.864 | -1.201 |
| RHEX | -0.105 | 0.007 | -0.097 | 0.092 | -0.324 | -0.443 | -0.446 | -2.021 |
| RHOBTB1 | -0.111 | 0.082 | 0.234 | 0.389 | -0.391 | -1.301 | -2.185 | -3.886 |
| RHOBTB2 | -0.173 | -0.028 | -0.026 | -0.037 | -0.568 | -0.682 | -0.277 | -0.311 |
| RHOBTB3 | 0.115 | -0.042 | 0.083 | 0.016 | -0.903 | -0.558 | -0.807 | -0.686 |
| RING1 | 0.005 | -0.083 | 0.084 | 0.134 | -0.446 | -0.147 | -1.011 | -1.252 |
| RLIM | 0.290 | 0.462 | -0.440 | -0.539 | -2.240 | -7.368 | -0.291 | -0.175 |
| RNF10 | -0.471 | -0.130 | 0.063 | 0.080 | -0.062 | -0.464 | -2.206 | -1.715 |
| RNF103 | -0.076 | -0.133 | 0.079 | 0.000 | -0.474 | -0.406 | -2.241 | -0.708 |
| RNF11 | -0.003 | 0.037 | 0.252 | 0.134 | -0.630 | -0.560 | -2.553 | -2.349 |
| RNF111 | 0.167 | 0.047 | -0.092 | -0.170 | -1.788 | -1.733 | -0.430 | -0.402 |
| RNF112 | 0.171 | 0.167 | -0.005 | -0.080 | -1.186 | -1.790 | -1.382 | -0.586 |
| RNF113A | 0.273 | -0.043 | -0.145 | -0.194 | -3.028 | -2.306 | -0.730 | -1.454 |
| RNF113B | -0.131 | -0.031 | -0.097 | -0.164 | -0.280 | -0.403 | -0.931 | -0.402 |
| RNF114 | 0.036 | 0.030 | 0.114 | 0.035 | -0.751 | -0.538 | -1.136 | -1.235 |
| RNF115 | -0.362 | -0.041 | -0.006 | 0.136 | -0.084 | -0.718 | -1.652 | -1.370 |
| RNF121 | 0.044 | 0.051 | -0.159 | -0.119 | -1.094 | -0.850 | -0.894 | -0.374 |
| RNF122 | -0.123 | -0.066 | -0.027 | -0.081 | -0.939 | -0.468 | -0.293 | -0.548 |
| RNF123 | 0.092 | -0.031 | -0.116 | -0.047 | -1.739 | -0.620 | -0.666 | -0.453 |
| RNF125 | -0.234 | -0.108 | -0.004 | 0.047 | -0.776 | -0.794 | -0.534 | -0.890 |
| RNF126 | 0.110 | 0.213 | 0.159 | 0.201 | -0.966 | -1.930 | -1.611 | -2.972 |
| RNF128 | -0.013 | 0.064 | -0.096 | -0.041 | -0.989 | -1.246 | -0.298 | -0.741 |
| RNF13 | -0.037 | 0.051 | -0.167 | -0.030 | -0.767 | -0.873 | -0.844 | -0.439 |
| RNF130 | -0.010 | -0.022 | -0.018 | 0.144 | -0.498 | -0.259 | -0.585 | -1.364 |
| RNF133 | -0.251 | 0.003 | -0.253 | 0.024 | -0.146 | -0.673 | -0.347 | -1.181 |
| RNF135 | 0.342 | -0.028 | -0.231 | -0.189 | -2.477 | -0.501 | -0.300 | -0.319 |
| RNF138 | -0.014 | 0.009 | -0.032 | -0.157 | -0.941 | -0.844 | -1.332 | -0.473 |
| RNF139 | -0.088 | -0.069 | -0.074 | -0.072 | -0.497 | -0.362 | -0.441 | -0.533 |
| RNF14 | -0.041 | -0.178 | -0.018 | 0.052 | -0.332 | -0.441 | -1.612 | -1.356 |
| RNF141 | 0.117 | 0.099 | -0.062 | -0.065 | -0.908 | -0.939 | -0.557 | -0.145 |
| RNF144A | -0.009 | 0.025 | 0.099 | -0.062 | -0.879 | -1.031 | -1.160 | -1.449 |
| RNF144B | -0.318 | -0.166 | 0.051 | -0.021 | -0.791 | -0.096 | -1.095 | -0.588 |
| RNF145 | -0.106 | -0.132 | -0.002 | -0.098 | -0.577 | -0.638 | -1.958 | -0.449 |
| RNF146 | -0.111 | -0.081 | 0.401 | 0.242 | -0.475 | -0.492 | -3.437 | -3.991 |
| RNF148 | -0.049 | -0.019 | 0.050 | -0.004 | -0.560 | -0.888 | -0.803 | -0.795 |
| RNF149 | -0.194 | -0.143 | 0.153 | 0.153 | -0.426 | -0.326 | -1.326 | -1.430 |
| RNF150 | 0.100 | 0.075 | -0.182 | 0.063 | -1.382 | -2.365 | -0.219 | -1.317 |
| RNF151 | -0.142 | -0.032 | -0.240 | -0.092 | -0.835 | -0.435 | -0.504 | -0.418 |
| RNF152 | 0.184 | -0.127 | 0.055 | -0.082 | -1.413 | -0.336 | -0.762 | -0.535 |
| RNF157 | 0.175 | 0.100 | -0.064 | -0.026 | -1.217 | -1.629 | -0.882 | -0.370 |
| RNF165 | -0.177 | -0.007 | 0.013 | 0.076 | -0.084 | -0.598 | -0.992 | -1.595 |
| RNF166 | -0.075 | -0.210 | -0.027 | -0.097 | -0.453 | -0.155 | -0.408 | -0.541 |
| RNF167 | 0.313 | 0.003 | -0.236 | 0.046 | -3.344 | -1.050 | -0.175 | -0.959 |
| RNF168 | 0.071 | -0.075 | 0.070 | -0.007 | -0.732 | -1.385 | -0.743 | -1.165 |
| RNF169 | 0.053 | 0.093 | -0.197 | -0.014 | -0.859 | -0.928 | -0.107 | -0.362 |
| RNF17 | -0.130 | 0.034 | -0.059 | 0.041 | -0.316 | -0.911 | -0.365 | -1.040 |
| RNF170 | -0.054 | -0.020 | -0.108 | -0.017 | -0.990 | -0.887 | -0.970 | -0.521 |
| RNF175 | -0.120 | -0.022 | 0.011 | -0.077 | -0.395 | -0.520 | -0.703 | -0.483 |
| RNF180 | 0.035 | -0.095 | 0.264 | 0.121 | -0.901 | -0.573 | -4.122 | -2.293 |
| RNF181 | -0.151 | 0.006 | 0.042 | 0.065 | -0.353 | -0.548 | -0.855 | -1.427 |
| RNF182 | -0.061 | -0.166 | 0.140 | -0.091 | -0.404 | -0.458 | -1.566 | -1.019 |
| RNF183 | -0.295 | -0.028 | 0.073 | 0.101 | -0.245 | -0.338 | -1.254 | -1.205 |
| RNF185 | -0.151 | -0.072 | -0.122 | -0.127 | -0.546 | -0.798 | -0.998 | -1.276 |
| RNF186 | -0.062 | -0.100 | 0.095 | 0.061 | -0.273 | -0.577 | -1.412 | -1.236 |
| RNF187 | -0.219 | -0.013 | -0.010 | 0.234 | -0.191 | -1.367 | -1.320 | -3.105 |
| RNF19A | -0.128 | -0.057 | -0.122 | -0.115 | -0.827 | -0.629 | -0.501 | -0.318 |
| RNF19B | -0.005 | -0.138 | -0.007 | 0.032 | -1.084 | -0.471 | -1.281 | -0.666 |
| RNF2 | -0.007 | 0.023 | 0.187 | 0.071 | -0.591 | -0.621 | -1.402 | -1.086 |
| RNF20 | -0.037 | 0.021 | -0.408 | -0.324 | -0.392 | -1.359 | -0.142 | -1.115 |
| RNF207 | 0.030 | 0.019 | -0.021 | 0.110 | -0.540 | -0.747 | -0.649 | -1.646 |
| RNF208 | 0.150 | 0.286 | -0.194 | 0.219 | -1.409 | -2.969 | -0.120 | -2.046 |
| RNF212 | -0.107 | 0.017 | -0.208 | -0.121 | -0.813 | -0.804 | -0.298 | -0.295 |
| RNF213 | -0.014 | 0.191 | -0.237 | -0.048 | -0.704 | -2.396 | -0.551 | -0.377 |
| RNF214 | 0.147 | 0.161 | -0.168 | 0.044 | -1.454 | -2.623 | -0.572 | -0.707 |
| RNF215 | 0.028 | -0.117 | -0.126 | -0.192 | -0.918 | -0.349 | -0.169 | -0.075 |
| RNF216 | 0.067 | -0.019 | -0.095 | -0.037 | -0.934 | -0.510 | -0.554 | -0.591 |
| RNF217 | -0.155 | -0.028 | 0.051 | 0.073 | -0.289 | -0.522 | -0.879 | -1.258 |
| RNF219 | 0.106 | 0.022 | -0.064 | -0.026 | -1.888 | -0.782 | -0.859 | -1.704 |
| RNF220 | -0.142 | -0.083 | 0.303 | 0.033 | -0.354 | -0.673 | -2.972 | -2.016 |
| RNF223 | 0.070 | -0.194 | 0.027 | -0.088 | -1.061 | -0.501 | -1.357 | -0.709 |
| RNF24 | 0.166 | 0.121 | 0.030 | 0.062 | -1.766 | -1.626 | -0.713 | -1.607 |
| RNF25 | -0.275 | -0.220 | -0.116 | 0.051 | -0.287 | -0.204 | -0.611 | -2.177 |
| RNF26 | 0.120 | 0.104 | -0.067 | -0.065 | -1.385 | -1.024 | -0.504 | -0.315 |
| RNF31 | 0.208 | 0.073 | -0.125 | 0.031 | -1.684 | -0.958 | -0.365 | -1.353 |
| RNF32 | -0.059 | -0.157 | -0.043 | -0.144 | -0.361 | -0.148 | -1.045 | -0.879 |
| RNF34 | -0.165 | -0.104 | -0.202 | 0.085 | -0.174 | -0.684 | -0.398 | -1.010 |
| RNF38 | -0.255 | -0.051 | -0.076 | -0.039 | -0.114 | -0.472 | -0.966 | -0.679 |
| RNF39 | 0.060 | -0.285 | 0.231 | -0.073 | -0.838 | -0.758 | -2.894 | -0.702 |
| RNF4 | -0.188 | -0.121 | -0.031 | 0.032 | -0.791 | -0.395 | -0.951 | -0.776 |
| RNF40 | 0.215 | 0.289 | -0.584 | -0.057 | -1.400 | -2.455 | -0.158 | -1.233 |
| RNF41 | -0.073 | 0.054 | 0.167 | -0.022 | -0.768 | -1.400 | -1.523 | -0.591 |
| RNF43 | 0.320 | 0.020 | -0.094 | -0.067 | -3.238 | -1.007 | -0.917 | -0.563 |
| RNF44 | -0.162 | -0.044 | 0.104 | 0.086 | -0.453 | -1.238 | -1.116 | -1.159 |
| RNF5 | 0.098 | -0.012 | 0.000 | 0.144 | -1.197 | -0.860 | -1.103 | -2.597 |
| RNF6 | -0.160 | -0.172 | -0.105 | -0.200 | -0.218 | -0.102 | -0.262 | -0.159 |
| RNF7 | 0.173 | 0.046 | 0.008 | -0.084 | -1.498 | -1.417 | -0.731 | -1.010 |
| RNF8 | -0.088 | -0.162 | -0.028 | -0.058 | -1.200 | -0.986 | -1.479 | -0.682 |
| RNFT1 | 0.021 | 0.141 | -0.065 | -0.098 | -0.999 | -2.395 | -0.641 | -0.911 |
| RNFT2 | 0.081 | 0.046 | -0.028 | 0.002 | -0.691 | -1.029 | -0.854 | -0.627 |
| RPA4 | 0.146 | 0.051 | -0.135 | 0.014 | -1.796 | -1.157 | -0.394 | -0.980 |
| RPL17 | 0.272 | 0.000 | 0.186 | 0.000 | -2.504 | -0.307 | -2.633 | -1.220 |
| RPS15A | 1.042 | 0.000 | -0.642 | -0.015 | -5.835 | -1.547 | -1.111 | -0.346 |
| RPS18 | 0.548 | 0.329 | -0.220 | 0.051 | -3.425 | -4.386 | -0.117 | -2.829 |
| RSPRY1 | -0.112 | -0.094 | 0.038 | -0.062 | -0.373 | -0.466 | -1.364 | -0.458 |
| RTN1 | 0.319 | 0.141 | -0.243 | 0.048 | -3.443 | -2.915 | -0.198 | -1.157 |
| RTRAF | 0.069 | -0.143 | 0.075 | 0.115 | -1.095 | -0.354 | -0.912 | -1.612 |
| SAE1 | 0.004 | -0.526 | 0.060 | -0.039 | -0.560 | -0.199 | -1.431 | -1.530 |
| SAR1B | -0.116 | -0.214 | 0.021 | -0.089 | -0.354 | -0.101 | -0.855 | -0.609 |
| SART3 | -0.360 | -0.052 | 0.219 | 0.372 | -0.291 | -0.232 | -2.138 | -4.677 |
| SCGB3A1 | 0.076 | 0.072 | -0.050 | -0.080 | -1.124 | -0.756 | -0.866 | -1.001 |
| SELE | -0.182 | -0.199 | 0.089 | 0.041 | -0.204 | -0.443 | -0.819 | -1.003 |
| SEMA3C | -0.062 | -0.028 | 0.041 | 0.077 | -0.354 | -1.089 | -1.926 | -2.747 |
| SEMA3F | -0.189 | -0.103 | -0.111 | -0.020 | -0.561 | -0.191 | -0.318 | -1.667 |
| SF3B2 | 0.039 | 0.291 | 0.207 | 0.056 | -1.802 | -3.367 | -2.497 | -0.829 |
| SF3B3 | -0.310 | -0.047 | 0.113 | -0.258 | -2.462 | -0.598 | -3.850 | -1.721 |
| SH3RF1 | -0.129 | 0.099 | 0.055 | 0.027 | -0.305 | -0.815 | -0.889 | -1.050 |
| SH3RF2 | -0.217 | -0.039 | -0.115 | 0.060 | -0.605 | -0.491 | -0.950 | -1.019 |
| SH3RF3 | -0.430 | -0.005 | 0.146 | 0.214 | -0.291 | -0.864 | -1.413 | -3.079 |
| SHARPIN | 0.017 | -0.142 | 0.124 | 0.070 | -0.393 | -0.434 | -1.392 | -0.891 |
| SHKBP1 | -0.125 | -0.156 | 0.585 | 0.398 | -0.738 | -0.402 | -7.258 | -4.617 |
| SHPRH | -0.030 | -0.092 | 0.034 | -0.055 | -1.236 | -0.858 | -1.030 | -0.676 |
| SIAH1 | -0.034 | 0.072 | 0.297 | 0.367 | -0.754 | -1.587 | -2.417 | -4.418 |
| SIAH2 | 0.030 | 0.025 | -0.082 | 0.014 | -0.870 | -1.244 | -0.475 | -1.063 |
| SIDT1 | 0.212 | 0.027 | -0.038 | -0.020 | -1.501 | -1.125 | -0.453 | -0.592 |
| SIRPD | 0.022 | 0.173 | 0.052 | 0.052 | -0.420 | -1.335 | -1.005 | -0.796 |
| SKP1 | 0.415 | 0.082 | -0.655 | -0.024 | -3.249 | -1.064 | -0.020 | -0.950 |
| SKP2 | -0.206 | -0.045 | 0.449 | -0.223 | -0.673 | -2.344 | -5.054 | -0.726 |
| SLX4 | -0.011 | 0.157 | 0.056 | -0.047 | -0.532 | -1.273 | -1.227 | -1.901 |
| SMU1 | 0.136 | 0.038 | -0.021 | 0.014 | -1.303 | -1.210 | -2.394 | -1.606 |
| SMURF1 | 0.160 | -0.048 | -0.005 | -0.006 | -2.922 | -0.381 | -1.737 | -1.816 |
| SMURF2 | -0.116 | 0.207 | -0.215 | -0.072 | -0.438 | -2.754 | -0.361 | -0.383 |
| SMYD3 | -0.087 | -0.211 | -0.010 | -0.036 | -0.294 | -0.239 | -1.048 | -0.772 |
| SOCS1 | 0.014 | -0.041 | -0.126 | 0.044 | -0.696 | -0.469 | -0.211 | -1.056 |
| SOCS2 | -0.034 | 0.048 | 0.339 | 0.038 | -1.189 | -0.936 | -2.753 | -0.669 |
| SOCS3 | 0.200 | 0.068 | 0.159 | -0.057 | -1.559 | -1.417 | -1.181 | -0.390 |
| SOCS4 | 0.297 | 0.034 | 0.044 | -0.001 | -4.345 | -0.598 | -0.778 | -1.109 |
| SOCS5 | 0.003 | -0.134 | 0.187 | 0.188 | -0.752 | -0.553 | -1.645 | -1.731 |
| SOCS6 | -0.121 | -0.048 | -0.133 | 0.128 | -0.257 | -0.639 | -0.535 | -1.734 |
| SOCS7 | -0.027 | 0.115 | -0.294 | -0.031 | -1.096 | -1.120 | -0.166 | -0.493 |
| SORCS3 | -0.266 | 0.003 | 0.052 | 0.046 | -0.357 | -0.604 | -0.971 | -1.245 |
| SOWAHC | 0.026 | 0.106 | 0.072 | 0.215 | -0.782 | -0.916 | -1.766 | -2.285 |
| SPATA2 | -0.046 | -0.042 | -0.139 | -0.082 | -0.468 | -0.822 | -0.458 | -0.258 |
| SPOP | 0.691 | 0.346 | 0.035 | 0.043 | -5.114 | -4.609 | -0.704 | -2.000 |
| SPOPL | -0.078 | 0.061 | 0.042 | -0.004 | -0.171 | -1.559 | -0.845 | -0.713 |
| SPSB1 | -0.280 | -0.035 | 0.021 | -0.010 | -0.049 | -0.260 | -0.506 | -1.394 |
| SPSB2 | -0.148 | 0.014 | -0.062 | 0.049 | -0.399 | -0.517 | -0.706 | -0.968 |
| SPSB3 | -0.044 | 0.078 | -0.206 | -0.055 | -0.697 | -0.794 | -0.075 | -0.291 |
| SPSB4 | -0.014 | 0.126 | 0.088 | 0.017 | -0.427 | -1.148 | -1.555 | -0.536 |
| SRSF3 | 0.155 | -0.039 | -0.107 | -0.021 | -1.691 | -0.981 | -0.913 | -1.054 |
| STAMBP | 0.038 | -0.059 | -0.364 | -0.415 | -0.705 | -0.463 | -0.198 | -0.060 |
| STAMBPL1 | -0.131 | -0.026 | 0.149 | 0.055 | -0.335 | -0.244 | -1.948 | -1.185 |
| STUB1 | 0.173 | -0.098 | -0.035 | 0.033 | -1.335 | -0.517 | -0.285 | -1.184 |
| SULT1A1 | -0.016 | -0.105 | -0.161 | -0.033 | -1.132 | -0.631 | -0.277 | -1.021 |
| SYNE2 | 0.340 | -0.047 | -0.114 | -0.135 | -4.869 | -0.361 | -0.424 | -0.334 |
| SYP | 0.082 | -0.056 | -0.195 | -0.190 | -0.857 | -0.597 | -0.629 | -0.273 |
| SYVN1 | 0.223 | 0.132 | -0.130 | -0.104 | -1.674 | -2.042 | -0.637 | -0.171 |
| TACR1 | -0.126 | 0.013 | 0.064 | 0.007 | -0.313 | -0.980 | -0.973 | -0.824 |
| TAF1D | -0.235 | -0.088 | 0.169 | -0.023 | -0.412 | -0.333 | -1.812 | -2.473 |
| TCEAL1 | 0.262 | 0.099 | -0.163 | 0.039 | -2.336 | -1.346 | -1.001 | -0.658 |
| TCEAL4 | 0.001 | -0.066 | -0.057 | -0.129 | -1.032 | -0.728 | -0.272 | -0.238 |
| TMEM168 | -0.138 | -0.089 | -0.032 | 0.019 | -0.548 | -0.834 | -0.927 | -0.622 |
| TMEM189 | -0.055 | 0.313 | -0.273 | -0.063 | -0.513 | -2.470 | -0.195 | -0.897 |
| TMEM189-UBE2V1 | -0.137 | -0.090 | -0.022 | -0.051 | -0.845 | -0.659 | -0.694 | -0.278 |
| TMPRSS11D | -0.033 | -0.058 | -0.101 | -0.035 | -0.647 | -1.403 | -0.794 | -0.464 |
| TNFAIP1 | 0.136 | 0.187 | -0.229 | 0.010 | -0.934 | -1.616 | -0.143 | -1.066 |
| TNFAIP3 | -0.071 | 0.009 | 0.096 | 0.154 | -0.428 | -1.314 | -0.845 | -2.350 |
| TOPORS | -0.173 | -0.075 | 0.021 | -0.070 | -0.221 | -0.484 | -0.685 | -0.671 |
| TOR1AIP1 | 0.087 | -0.073 | 0.170 | 0.050 | -0.923 | -0.542 | -1.733 | -0.961 |
| TOR1AIP2 | -0.068 | -0.142 | 0.160 | 0.076 | -0.618 | -0.955 | -3.685 | -1.868 |
| TRAF2 | 0.231 | -0.306 | 0.253 | -0.021 | -1.556 | -0.075 | -2.034 | -1.310 |
| TRAF3 | -0.036 | -0.042 | 0.179 | 0.217 | -0.601 | -0.250 | -2.661 | -2.903 |
| TRAF4 | 0.118 | 0.232 | -0.201 | -0.167 | -1.952 | -2.763 | -0.441 | -0.337 |
| TRAF5 | 0.017 | 0.011 | 0.125 | 0.096 | -1.382 | -0.831 | -1.492 | -1.875 |
| TRAF6 | 0.237 | 0.188 | -0.250 | -0.134 | -1.724 | -1.620 | -0.352 | -0.603 |
| TRAF7 | 0.006 | 0.092 | -0.317 | 0.057 | -1.006 | -0.872 | -0.578 | -0.746 |
| TRAIP | -0.226 | -0.006 | -0.051 | 0.090 | -0.070 | -1.648 | -0.640 | -1.770 |
| TRIM10 | -0.198 | -0.083 | 0.153 | 0.039 | -0.556 | -0.771 | -1.783 | -0.775 |
| TRIM11 | -0.123 | -0.161 | 0.137 | 0.167 | -0.502 | -0.356 | -2.024 | -2.128 |
| TRIM13 | -0.005 | -0.036 | -0.076 | -0.043 | -0.581 | -0.218 | -0.651 | -0.361 |
| TRIM14 | -0.210 | -0.083 | -0.192 | 0.034 | -0.175 | -1.086 | -0.232 | -0.674 |
| TRIM15 | -0.259 | -0.012 | 0.160 | 0.103 | -0.499 | -1.275 | -1.741 | -1.345 |
| TRIM17 | -0.257 | -0.018 | 0.157 | 0.050 | -0.634 | -0.514 | -1.811 | -1.945 |
| TRIM2 | -0.182 | 0.038 | 0.041 | -0.100 | -0.498 | -0.632 | -0.800 | -0.749 |
| TRIM21 | -0.021 | -0.010 | -0.022 | -0.133 | -0.807 | -0.721 | -0.398 | -0.672 |
| TRIM22 | -0.019 | 0.051 | -0.016 | -0.138 | -0.743 | -0.665 | -0.404 | -0.411 |
| TRIM23 | -0.195 | -0.174 | 0.114 | 0.083 | -0.170 | -0.225 | -1.367 | -1.737 |
| TRIM24 | -0.129 | -0.100 | 0.037 | -0.077 | -0.188 | -0.386 | -1.056 | -0.455 |
| TRIM25 | 0.254 | 0.028 | -0.241 | -0.005 | -1.711 | -0.660 | -0.224 | -0.460 |
| TRIM26 | -0.147 | -0.145 | 0.429 | 0.122 | -0.713 | -0.406 | -4.644 | -1.098 |
| TRIM27 | 0.007 | -0.035 | 0.211 | 0.004 | -0.439 | -0.261 | -1.426 | -0.878 |
| TRIM28 | -0.164 | 0.415 | 0.431 | 0.029 | -0.250 | -4.466 | -4.075 | -1.586 |
| TRIM3 | 0.129 | 0.147 | -0.081 | -0.194 | -0.847 | -1.372 | -1.159 | -0.324 |
| TRIM31 | -0.057 | 0.041 | 0.103 | 0.176 | -0.325 | -0.737 | -1.972 | -1.777 |
| TRIM32 | -0.155 | -0.094 | -0.064 | 0.137 | -0.618 | -0.227 | -1.106 | -1.447 |
| TRIM33 | -0.083 | -0.051 | 0.095 | -0.021 | -0.276 | -0.479 | -0.880 | -0.564 |
| TRIM34 | 0.035 | 0.084 | -0.067 | -0.035 | -0.829 | -0.935 | -0.756 | -0.329 |
| TRIM35 | 0.023 | 0.056 | 0.051 | -0.033 | -0.880 | -0.862 | -0.910 | -0.802 |
| TRIM36 | 0.060 | -0.083 | 0.072 | -0.055 | -1.828 | -0.370 | -2.748 | -0.920 |
| TRIM37 | 0.015 | -0.094 | 0.042 | -0.406 | -0.741 | -0.165 | -2.020 | -0.275 |
| TRIM38 | -0.326 | -0.057 | 0.114 | -0.071 | -0.310 | -0.283 | -1.174 | -0.463 |
| TRIM39 | -0.089 | -0.176 | 0.244 | 0.146 | -0.336 | -0.178 | -2.580 | -1.494 |
| TRIM4 | -0.253 | -0.075 | 0.232 | 0.082 | -0.493 | -0.295 | -1.734 | -1.470 |
| TRIM40 | -0.072 | -0.034 | 0.039 | 0.139 | -0.273 | -1.121 | -0.898 | -2.798 |
| TRIM41 | -0.172 | -0.209 | 0.115 | 0.057 | -0.305 | -0.291 | -1.202 | -0.705 |
| TRIM42 | 0.041 | 0.069 | -0.126 | -0.005 | -0.913 | -1.112 | -0.366 | -0.824 |
| TRIM43 | -0.143 | 0.012 | 0.451 | 0.086 | -0.121 | -0.628 | -3.636 | -1.436 |
| TRIM45 | 0.023 | -0.087 | 0.350 | -0.024 | -0.824 | -0.161 | -4.252 | -1.524 |
| TRIM46 | -0.026 | -0.062 | 0.137 | 0.160 | -0.822 | -0.262 | -1.261 | -1.923 |
| TRIM47 | 0.180 | 0.181 | -0.149 | -0.154 | -1.491 | -2.178 | -0.094 | -1.134 |
| TRIM48 | 0.349 | 0.458 | -0.062 | -0.224 | -4.764 | -3.730 | -0.952 | -0.074 |
| TRIM49 | 0.324 | 0.220 | -0.072 | -0.254 | -2.430 | -2.334 | -0.500 | -0.042 |
| TRIM49B | 0.356 | 0.190 | -0.073 | -0.113 | -2.528 | -1.649 | -0.445 | -0.248 |
| TRIM49D1 | 0.460 | 0.645 | -0.154 | -0.099 | -2.712 | -5.570 | -0.187 | -0.229 |
| TRIM5 | 0.084 | 0.006 | -0.071 | -0.099 | -0.852 | -0.714 | -0.552 | -0.477 |
| TRIM50 | -0.323 | -0.059 | -0.007 | 0.168 | -1.153 | -0.596 | -0.858 | -2.720 |
| TRIM51 | 0.624 | 0.342 | -0.281 | -0.236 | -4.526 | -4.106 | -0.557 | -0.445 |
| TRIM52 | 0.121 | -0.033 | -0.019 | -0.010 | -0.973 | -0.890 | -0.805 | -1.578 |
| TRIM54 | -0.125 | -0.016 | -0.164 | 0.045 | -0.797 | -0.799 | -0.584 | -1.309 |
| TRIM55 | 0.114 | -0.036 | -0.032 | -0.059 | -1.334 | -0.884 | -1.350 | -0.492 |
| TRIM56 | -0.023 | -0.075 | 0.060 | -0.148 | -0.710 | -1.986 | -0.634 | -2.763 |
| TRIM58 | -0.252 | -0.070 | -0.088 | -0.002 | -0.423 | -0.560 | -0.578 | -0.967 |
| TRIM59 | 0.080 | -0.152 | -0.083 | -0.065 | -0.988 | -0.389 | -0.392 | -1.046 |
| TRIM6 | -0.082 | 0.141 | -0.032 | -0.059 | -0.583 | -1.549 | -0.822 | -0.551 |
| TRIM60 | 0.097 | 0.055 | -0.003 | -0.044 | -0.879 | -0.666 | -0.607 | -0.323 |
| TRIM61 | 0.010 | -0.005 | -0.157 | -0.152 | -1.336 | -0.940 | -0.952 | -0.426 |
| TRIM62 | -0.130 | -0.066 | 0.160 | 0.139 | -0.396 | -1.069 | -1.764 | -1.579 |
| TRIM63 | -0.070 | 0.011 | 0.062 | 0.062 | -0.434 | -0.980 | -1.156 | -2.481 |
| TRIM64 | 0.379 | 0.346 | -0.069 | -0.269 | -3.731 | -3.556 | -0.849 | -0.142 |
| TRIM65 | -0.108 | 0.086 | -0.298 | -0.139 | -0.670 | -0.892 | -0.300 | -0.298 |
| TRIM67 | -0.218 | -0.038 | 0.197 | 0.167 | -0.363 | -0.456 | -2.360 | -2.135 |
| TRIM68 | 0.152 | 0.091 | -0.030 | -0.129 | -1.618 | -0.776 | -0.474 | -0.313 |
| TRIM69 | -0.056 | 0.248 | -0.140 | -0.173 | -0.591 | -2.121 | -0.553 | -0.314 |
| TRIM6-TRIM34 | -0.080 | 0.149 | -0.071 | -0.128 | -0.463 | -1.239 | -0.730 | -0.708 |
| TRIM7 | 0.034 | -0.012 | 0.090 | -0.011 | -1.825 | -0.451 | -1.838 | -0.835 |
| TRIM71 | -0.222 | 0.035 | 0.043 | 0.179 | -0.279 | -1.254 | -0.787 | -2.903 |
| TRIM72 | -0.062 | -0.084 | 0.002 | -0.065 | -0.416 | -0.392 | -0.786 | -0.245 |
| TRIM73 | -0.125 | -0.105 | 0.155 | 0.052 | -0.429 | -0.313 | -1.551 | -0.696 |
| TRIM77 | 0.196 | 0.174 | -0.258 | -0.066 | -1.740 | -1.727 | -0.439 | -0.413 |
| TRIM8 | -0.384 | -0.108 | -0.055 | 0.011 | -0.208 | -0.501 | -0.445 | -1.264 |
| TRIM9 | 0.155 | -0.019 | -0.146 | -0.020 | -2.374 | -0.478 | -0.306 | -1.307 |
| TRIML1 | -0.063 | -0.179 | -0.009 | -0.102 | -2.049 | -0.348 | -1.574 | -0.641 |
| TRIP12 | 0.339 | 0.385 | -0.396 | -0.480 | -2.546 | -3.346 | -0.290 | -0.088 |
| TRPC4AP | 0.018 | 0.018 | -0.113 | -0.026 | -0.693 | -0.809 | -0.548 | -0.574 |
| TRPV6 | 0.021 | -0.008 | -0.056 | 0.089 | -0.775 | -0.508 | -0.471 | -1.083 |
| TSG101 | 0.091 | 0.181 | -0.722 | -0.259 | -1.266 | -2.111 | -0.069 | -1.332 |
| TSPAN13 | -0.136 | -0.157 | 0.044 | 0.100 | -0.301 | -0.106 | -1.174 | -2.297 |
| TSPAN17 | -0.381 | -0.215 | 0.001 | -0.097 | -0.046 | -0.485 | -0.387 | -0.188 |
| TTBK1 | -0.224 | -0.002 | -0.018 | 0.261 | -0.363 | -0.347 | -1.261 | -2.453 |
| TTC3 | -0.072 | -0.036 | 0.007 | 0.020 | -1.323 | -0.497 | -0.815 | -1.212 |
| TULP4 | -0.119 | -0.073 | -0.003 | 0.041 | -0.200 | -0.993 | -1.088 | -1.842 |
| UBA1 | 1.190 | 0.588 | -0.478 | -0.342 | -9.205 | -6.272 | -0.076 | -1.287 |
| UBA2 | -0.343 | 0.012 | -0.088 | -0.196 | -0.255 | -3.087 | -1.048 | -0.700 |
| UBA3 | 1.531 | 0.530 | -0.931 | -0.649 | -14.077 | -4.238 | -0.717 | -1.447 |
| UBA5 | -0.001 | 0.093 | 0.097 | 0.112 | -0.851 | -1.391 | -1.184 | -1.837 |
| UBA6 | -0.047 | -0.062 | 0.133 | -0.024 | -0.350 | -0.468 | -3.661 | -0.921 |
| UBA7 | -0.142 | -0.079 | -0.003 | -0.060 | -0.357 | -0.391 | -0.369 | -0.204 |
| UBE2A | 0.587 | 0.590 | -0.526 | -0.464 | -5.286 | -5.167 | -0.049 | -0.034 |
| UBE2B | -0.210 | 0.006 | -0.045 | 0.081 | -0.430 | -0.831 | -1.383 | -1.204 |
| UBE2C | 0.027 | 0.612 | -0.231 | -0.078 | -1.173 | -7.147 | -0.176 | -0.309 |
| UBE2D1 | -0.049 | 0.105 | 0.096 | 0.050 | -0.661 | -1.079 | -1.329 | -1.090 |
| UBE2D2 | 0.070 | 0.098 | -0.089 | 0.019 | -0.969 | -1.108 | -0.277 | -0.555 |
| UBE2D3 | 0.416 | 0.229 | -0.494 | -0.424 | -5.503 | -2.025 | -0.152 | -0.095 |
| UBE2D4 | -0.098 | -0.126 | -0.129 | 0.103 | -0.757 | -0.670 | -0.496 | -1.994 |
| UBE2E2 | -0.075 | -0.049 | -0.039 | 0.075 | -1.464 | -0.262 | -0.638 | -1.551 |
| UBE2E3 | 0.199 | -0.161 | 0.251 | 0.034 | -1.483 | -0.410 | -2.459 | -0.616 |
| UBE2F | 0.110 | 0.133 | -0.136 | 0.004 | -1.967 | -4.127 | -0.507 | -1.213 |
| UBE2G1 | 0.260 | -0.190 | 0.141 | 0.156 | -1.632 | -0.139 | -1.528 | -1.989 |
| UBE2G2 | 0.015 | -0.162 | 0.102 | 0.242 | -0.425 | -0.098 | -1.051 | -2.624 |
| UBE2H | 0.051 | 0.009 | -0.102 | -0.359 | -0.737 | -0.605 | -0.281 | -0.247 |
| UBE2I | -0.356 | -0.482 | -0.596 | -0.591 | -0.929 | -1.301 | -2.220 | -0.894 |
| UBE2J1 | 0.057 | -0.036 | 0.169 | 0.020 | -1.294 | -0.512 | -1.284 | -0.545 |
| UBE2J2 | -0.191 | -0.071 | -0.007 | 0.008 | -0.829 | -0.275 | -0.869 | -0.475 |
| UBE2K | 0.428 | 0.548 | -0.779 | -0.308 | -5.476 | -8.285 | -0.058 | -0.081 |
| UBE2L3 | 0.701 | 0.389 | 0.335 | 0.299 | -5.310 | -4.176 | -2.970 | -4.042 |
| UBE2L6 | 0.134 | 0.123 | -0.187 | -0.157 | -0.873 | -2.315 | -0.377 | -0.144 |
| UBE2M | 0.745 | 0.755 | -0.239 | -0.096 | -11.187 | -6.079 | -0.069 | -2.642 |
| UBE2N | -0.089 | -0.038 | 0.134 | 0.177 | -0.417 | -0.228 | -1.954 | -1.915 |
| UBE2NL | 0.067 | 0.075 | 0.090 | 0.144 | -0.669 | -1.710 | -0.763 | -1.365 |
| UBE2O | 0.151 | 0.302 | -0.356 | -0.105 | -1.401 | -3.597 | -0.096 | -0.333 |
| UBE2Q1 | -0.187 | -0.208 | 0.406 | 0.253 | -0.295 | -0.164 | -4.985 | -3.869 |
| UBE2Q2 | 0.165 | 0.156 | -0.052 | 0.008 | -1.267 | -2.251 | -0.857 | -1.374 |
| UBE2QL1 | 0.056 | -0.245 | 0.268 | 0.031 | -1.338 | -0.548 | -2.069 | -1.108 |
| UBE2R2 | -0.051 | -0.046 | 0.072 | 0.254 | -0.278 | -0.240 | -0.746 | -2.746 |
| UBE2S | -0.035 | 0.136 | 0.190 | 0.169 | -0.887 | -2.785 | -1.317 | -1.767 |
| UBE2T | -0.052 | -0.069 | -0.027 | 0.126 | -0.863 | -1.332 | -0.829 | -1.501 |
| UBE2U | -0.130 | 0.008 | 0.008 | 0.059 | -0.937 | -0.672 | -0.604 | -1.745 |
| UBE2V1 | -0.054 | -0.017 | -0.066 | -0.211 | -1.478 | -0.482 | -0.446 | -0.506 |
| UBE2V2 | 0.123 | -0.010 | -0.099 | -0.183 | -1.784 | -0.414 | -0.821 | -0.306 |
| UBE2W | -0.320 | -0.011 | -0.020 | 0.191 | -0.116 | -0.779 | -0.956 | -2.485 |
| UBE2Z | -0.008 | -0.085 | 0.101 | 0.149 | -1.720 | -0.411 | -1.340 | -1.791 |
| UBE3A | -0.024 | 0.144 | 0.102 | -0.058 | -0.710 | -1.740 | -1.480 | -0.440 |
| UBE3B | -0.112 | -0.129 | 0.071 | -0.130 | -0.209 | -0.427 | -2.000 | -0.929 |
| UBE3C | 0.142 | 0.208 | -0.177 | -0.198 | -1.049 | -3.163 | -0.469 | -0.283 |
| UBE3D | -0.153 | 0.108 | -0.076 | 0.046 | -0.709 | -0.908 | -0.993 | -0.894 |
| UBE4A | 0.192 | 0.098 | -0.212 | -0.077 | -1.431 | -0.998 | -0.226 | -0.798 |
| UBE4B | 0.012 | 0.088 | -0.007 | 0.042 | -0.781 | -0.778 | -0.591 | -0.916 |
| UBL4A | 0.102 | 0.102 | -0.202 | -0.057 | -1.000 | -1.299 | -0.237 | -0.210 |
| UBOX5 | 0.258 | 0.178 | -0.289 | -0.218 | -2.646 | -2.543 | -0.207 | -0.412 |
| UBR1 | 0.008 | 0.003 | -0.030 | -0.033 | -0.580 | -0.897 | -0.757 | -1.101 |
| UBR2 | 0.095 | 0.103 | 0.003 | 0.040 | -0.938 | -1.149 | -1.605 | -2.720 |
| UBR3 | -0.023 | 0.021 | 0.232 | 0.021 | -0.601 | -0.770 | -2.048 | -1.238 |
| UBR4 | 0.357 | 0.273 | 0.156 | -0.439 | -2.395 | -3.849 | -2.977 | -0.876 |
| UBR5 | 1.524 | 1.525 | -1.166 | -1.198 | -10.698 | -15.368 | -0.002 | -0.012 |
| UBR7 | -0.028 | 0.042 | 0.004 | -0.048 | -0.511 | -0.951 | -0.621 | -0.669 |
| UCHL1 | -0.118 | 0.075 | -0.077 | -0.078 | -0.132 | -0.649 | -0.935 | -1.020 |
| UCHL3 | 0.067 | 0.035 | -0.038 | -0.012 | -1.258 | -1.574 | -0.683 | -1.565 |
| UCHL5 | -0.153 | -0.136 | 0.219 | 0.201 | -0.184 | -0.105 | -1.534 | -2.982 |
| UFC1 | -0.386 | -0.139 | 0.208 | 0.212 | -0.035 | -0.355 | -2.109 | -3.539 |
| UFD1 | 0.309 | -0.131 | 0.017 | -0.191 | -3.737 | -1.920 | -0.475 | -0.752 |
| UHRF1 | -0.484 | -0.579 | 0.364 | -0.251 | -0.084 | -0.867 | -3.927 | -0.977 |
| UHRF2 | 0.014 | 0.026 | -0.098 | 0.041 | -1.131 | -1.170 | -0.992 | -1.332 |
| UIMC1 | -0.105 | -0.150 | 0.117 | -0.116 | -0.559 | -0.621 | -2.382 | -0.976 |
| UNC5C | 0.124 | 0.048 | -0.101 | -0.189 | -1.692 | -1.429 | -0.873 | -0.204 |
| UNK | -0.054 | 0.187 | -0.101 | 0.022 | -0.481 | -2.156 | -0.318 | -1.263 |
| UNKL | 0.087 | 0.144 | -0.077 | -0.001 | -1.952 | -2.186 | -0.647 | -0.880 |
| USP1 | -0.305 | -0.105 | 0.184 | -0.161 | -0.303 | -0.481 | -3.329 | -0.921 |
| USP10 | -0.135 | 0.036 | -0.508 | -0.128 | -1.009 | -0.733 | -0.193 | -0.117 |
| USP11 | 0.173 | -0.153 | -0.015 | -0.139 | -1.875 | -0.334 | -0.489 | -0.450 |
| USP12 | -0.102 | 0.019 | 0.157 | 0.091 | -1.115 | -1.049 | -1.899 | -1.015 |
| USP13 | 0.032 | 0.029 | 0.139 | -0.009 | -0.678 | -0.654 | -1.084 | -0.463 |
| USP14 | 0.117 | 0.252 | -0.118 | 0.111 | -1.830 | -2.119 | -0.249 | -1.426 |
| USP15 | 0.109 | 0.042 | -0.024 | -0.002 | -1.081 | -0.848 | -1.198 | -1.132 |
| USP16 | -0.165 | -0.022 | -0.095 | -0.122 | -0.925 | -0.587 | -0.494 | -0.578 |
| USP18 | 0.035 | 0.085 | -0.067 | -0.010 | -0.779 | -1.242 | -1.076 | -0.384 |
| USP19 | -0.330 | -0.270 | 0.066 | -0.037 | -0.081 | -0.197 | -0.754 | -0.470 |
| USP2 | 0.010 | 0.153 | 0.111 | -0.120 | -1.765 | -1.228 | -0.886 | -0.480 |
| USP20 | -0.120 | 0.025 | -0.125 | -0.132 | -0.233 | -1.589 | -0.506 | -0.333 |
| USP21 | -0.160 | -0.065 | 0.200 | 0.073 | -0.435 | -0.735 | -1.714 | -0.995 |
| USP22 | -0.357 | -0.455 | 0.425 | 0.288 | -0.129 | -0.073 | -4.513 | -2.968 |
| USP25 | -0.335 | -0.249 | -0.116 | -0.009 | -0.293 | -0.306 | -0.806 | -1.179 |
| USP26 | 0.023 | 0.115 | 0.006 | 0.013 | -0.714 | -1.408 | -0.529 | -1.154 |
| USP28 | -0.049 | -0.017 | -0.180 | -0.054 | -1.071 | -1.192 | -0.423 | -1.213 |
| USP29 | -0.318 | -0.165 | 0.168 | 0.201 | -0.386 | -0.409 | -2.173 | -2.576 |
| USP3 | 0.064 | 0.207 | -0.027 | -0.002 | -0.989 | -3.043 | -0.661 | -0.855 |
| USP30 | 0.014 | -0.069 | 0.035 | 0.060 | -0.501 | -0.457 | -1.610 | -1.157 |
| USP32 | 0.136 | -0.026 | 0.104 | 0.039 | -1.056 | -1.244 | -1.264 | -0.838 |
| USP33 | -0.013 | -0.115 | 0.001 | 0.101 | -1.145 | -0.349 | -0.749 | -1.673 |
| USP36 | 0.463 | 0.492 | -0.756 | -0.084 | -4.898 | -5.441 | -0.062 | -0.915 |
| USP37 | 0.150 | 0.188 | -0.086 | -0.088 | -1.683 | -2.838 | -0.847 | -0.963 |
| USP38 | -0.076 | 0.029 | -0.182 | -0.142 | -1.909 | -0.921 | -0.251 | -0.553 |
| USP39 | -0.243 | 0.183 | 1.091 | 0.409 | -0.196 | -1.967 | -13.353 | -5.991 |
| USP4 | -0.050 | 0.018 | -0.011 | 0.024 | -0.940 | -0.395 | -0.735 | -1.084 |
| USP42 | -0.212 | -0.100 | -0.033 | -0.020 | -0.277 | -0.447 | -1.345 | -0.692 |
| USP43 | 0.094 | 0.040 | -0.039 | 0.049 | -1.093 | -2.439 | -0.320 | -1.207 |
| USP44 | -0.263 | -0.135 | 0.039 | 0.046 | -0.307 | -0.591 | -1.293 | -0.956 |
| USP45 | 0.040 | -0.089 | -0.073 | 0.109 | -0.612 | -0.302 | -0.651 | -1.905 |
| USP46 | -0.086 | -0.110 | -0.078 | -0.086 | -0.620 | -0.811 | -0.704 | -0.195 |
| USP48 | 0.009 | 0.092 | -0.107 | -0.099 | -0.381 | -1.517 | -0.765 | -0.462 |
| USP49 | -0.076 | -0.097 | 0.361 | 0.095 | -0.388 | -0.411 | -3.384 | -1.229 |
| USP5 | 1.580 | 0.286 | -0.423 | 0.043 | -9.654 | -2.392 | -0.050 | -0.717 |
| USP50 | 0.179 | 0.218 | -0.099 | -0.207 | -1.911 | -3.005 | -0.617 | -0.464 |
| USP53 | 0.002 | -0.069 | -0.097 | -0.053 | -0.498 | -0.900 | -1.217 | -0.720 |
| USP7 | -0.183 | 0.103 | 0.079 | 0.223 | -0.394 | -1.370 | -0.970 | -2.399 |
| USP8 | 0.265 | 0.094 | -0.602 | -0.514 | -1.903 | -1.448 | -0.129 | -0.089 |
| USPL1 | -0.013 | -0.162 | 0.123 | 0.075 | -0.540 | -0.533 | -1.279 | -1.836 |
| VARS | 0.093 | -0.030 | -0.066 | 0.049 | -1.233 | -1.220 | -0.853 | -1.328 |
| VCP | 0.877 | 0.422 | -0.490 | 0.326 | -6.178 | -4.120 | -0.825 | -3.997 |
| VCPIP1 | 0.088 | 0.123 | -0.028 | 0.056 | -1.133 | -1.826 | -0.834 | -1.270 |
| VHL | 0.024 | 0.035 | -0.131 | 0.033 | -0.521 | -0.940 | -0.757 | -1.341 |
| VPS11 | 0.397 | 0.296 | -0.350 | -0.268 | -3.520 | -3.176 | -0.191 | -0.149 |
| VPS18 | 0.529 | 0.259 | -0.295 | -0.463 | -4.255 | -4.927 | -0.314 | -0.143 |
| VPS28 | 0.174 | -0.189 | -1.125 | 0.010 | -1.885 | -1.451 | -0.005 | -1.032 |
| VPS41 | -0.132 | 0.019 | -0.061 | -0.026 | -0.634 | -0.866 | -0.815 | -0.459 |
| VPS8 | -0.063 | 0.006 | -0.102 | -0.016 | -1.614 | -0.699 | -0.785 | -1.651 |
| WDR20 | -0.072 | 0.029 | 0.061 | 0.027 | -0.893 | -0.610 | -0.767 | -0.826 |
| WDR48 | -0.364 | -0.167 | 0.374 | 0.189 | -0.522 | -0.406 | -3.587 | -1.867 |
| WDSUB1 | 0.003 | -0.066 | 0.379 | -0.002 | -0.738 | -0.381 | -3.439 | -0.447 |
| WDTC1 | -0.138 | 0.001 | -0.037 | -0.089 | -0.626 | -0.320 | -0.334 | -0.676 |
| WSB1 | -0.026 | -0.065 | 0.109 | 0.045 | -0.534 | -0.145 | -2.726 | -1.015 |
| WSB2 | -0.004 | -0.035 | 0.161 | 0.032 | -0.906 | -1.086 | -2.509 | -0.851 |
| WWP1 | 0.009 | 0.137 | -0.082 | 0.065 | -0.933 | -1.500 | -0.720 | -0.918 |
| WWP2 | -0.145 | -0.044 | 0.081 | 0.075 | -0.151 | -0.513 | -1.104 | -1.495 |
| XIAP | 0.278 | 0.399 | -0.082 | 0.218 | -2.415 | -3.572 | -0.733 | -3.022 |
| ZBTB1 | 0.182 | -0.093 | -0.047 | 0.064 | -1.564 | -0.625 | -0.705 | -2.145 |
| ZBTB10 | -0.083 | 0.022 | 0.178 | 0.195 | -0.776 | -0.989 | -4.561 | -2.648 |
| ZBTB11 | 0.791 | 0.359 | -0.105 | -0.103 | -7.284 | -2.833 | -0.563 | -0.735 |
| ZBTB12 | 0.030 | -0.048 | 0.103 | -0.056 | -0.844 | -0.381 | -0.940 | -0.389 |
| ZBTB14 | -0.223 | -0.108 | 0.175 | 0.046 | -0.316 | -0.579 | -1.785 | -1.812 |
| ZBTB16 | 0.050 | -0.159 | -0.270 | -0.091 | -0.869 | -0.884 | -0.278 | -0.869 |
| ZBTB17 | -0.573 | -0.298 | 0.703 | 0.368 | -0.002 | -0.635 | -8.511 | -4.634 |
| ZBTB18 | 0.069 | -0.004 | 0.111 | 0.072 | -0.717 | -0.601 | -1.215 | -1.297 |
| ZBTB2 | -0.116 | 0.133 | 0.111 | 0.306 | -0.243 | -1.041 | -1.893 | -6.016 |
| ZBTB20 | -0.042 | 0.103 | 0.096 | -0.012 | -0.279 | -1.275 | -0.905 | -0.764 |
| ZBTB21 | -0.288 | -0.088 | 0.214 | 0.011 | -0.086 | -0.434 | -1.531 | -1.045 |
| ZBTB22 | -0.281 | -0.057 | -0.064 | 0.027 | -0.125 | -0.471 | -1.368 | -0.493 |
| ZBTB24 | -0.239 | -0.153 | 0.027 | 0.148 | -0.246 | -0.188 | -1.622 | -2.351 |
| ZBTB25 | -0.001 | -0.090 | -0.048 | -0.071 | -0.779 | -0.435 | -0.478 | -0.677 |
| ZBTB26 | -0.205 | -0.100 | -0.072 | -0.064 | -0.531 | -0.822 | -0.589 | -0.807 |
| ZBTB3 | 0.206 | 0.054 | -0.284 | -0.214 | -2.338 | -0.996 | -0.176 | -0.293 |
| ZBTB32 | -0.039 | -0.161 | 0.611 | 0.332 | -0.284 | -0.152 | -8.326 | -3.203 |
| ZBTB33 | 0.079 | 0.024 | -0.062 | 0.081 | -1.028 | -0.751 | -0.718 | -2.240 |
| ZBTB37 | -0.021 | 0.004 | 0.024 | 0.082 | -0.311 | -0.929 | -0.852 | -1.416 |
| ZBTB38 | 0.150 | 0.137 | -0.067 | 0.061 | -1.939 | -1.483 | -0.591 | -1.016 |
| ZBTB39 | -0.255 | 0.029 | 0.100 | 0.000 | -0.110 | -0.876 | -0.868 | -1.093 |
| ZBTB4 | 0.057 | 0.206 | -0.163 | 0.143 | -1.137 | -1.991 | -0.253 | -1.456 |
| ZBTB40 | -0.145 | 0.116 | 0.008 | 0.065 | -0.199 | -1.150 | -0.457 | -1.628 |
| ZBTB41 | -0.144 | 0.002 | -0.080 | 0.094 | -0.653 | -0.561 | -1.591 | -1.454 |
| ZBTB43 | -0.179 | 0.042 | -0.056 | 0.011 | -0.627 | -1.160 | -0.761 | -0.986 |
| ZBTB44 | -0.054 | 0.073 | -0.127 | -0.191 | -0.421 | -0.771 | -0.666 | -0.222 |
| ZBTB45 | -0.196 | -0.126 | 0.499 | 0.439 | -0.087 | -0.207 | -5.913 | -7.227 |
| ZBTB46 | 0.127 | 0.116 | 0.001 | -0.034 | -1.071 | -1.107 | -1.123 | -0.916 |
| ZBTB48 | -0.019 | 0.105 | 0.125 | 0.170 | -0.702 | -1.580 | -1.504 | -3.451 |
| ZBTB49 | -0.121 | 0.067 | 0.061 | -0.058 | -0.263 | -0.753 | -1.283 | -0.413 |
| ZBTB5 | -0.027 | 0.036 | 0.079 | -0.096 | -1.305 | -1.276 | -1.567 | -0.905 |
| ZBTB6 | -0.161 | -0.126 | -0.041 | -0.090 | -0.085 | -0.493 | -0.544 | -0.542 |
| ZBTB7A | 0.059 | 0.142 | 0.181 | 0.068 | -0.943 | -1.501 | -1.703 | -1.112 |
| ZBTB7B | -0.272 | 0.050 | 0.303 | 0.126 | -0.269 | -0.947 | -3.901 | -2.372 |
| ZBTB7C | -0.060 | -0.154 | -0.050 | -0.030 | -0.885 | -0.338 | -0.603 | -0.430 |
| ZBTB8A | -0.293 | -0.088 | 0.158 | 0.025 | -0.158 | -0.645 | -1.392 | -1.172 |
| ZBTB9 | -0.106 | -0.054 | 0.008 | 0.086 | -0.275 | -0.406 | -0.899 | -1.721 |
| ZFAND3 | -0.160 | -0.110 | 0.089 | -0.022 | -0.174 | -0.558 | -0.967 | -0.646 |
| ZFAND4 | 0.025 | -0.146 | 0.155 | -0.008 | -0.804 | -0.749 | -1.078 | -1.178 |
| ZFAND5 | 0.110 | -0.005 | 0.001 | 0.036 | -1.323 | -0.846 | -0.575 | -0.598 |
| ZFAND6 | 0.127 | 0.095 | 0.118 | -0.166 | -1.560 | -1.889 | -0.895 | -0.230 |
| ZFPL1 | 0.079 | -0.058 | -0.069 | -0.142 | -1.231 | -0.750 | -0.653 | -0.606 |
| ZNF131 | 0.042 | -0.036 | -0.029 | -0.054 | -1.207 | -1.716 | -1.242 | -0.220 |
| ZNF598 | -0.310 | 0.046 | 0.078 | 0.139 | -0.294 | -0.693 | -1.109 | -2.785 |
| ZNF7 | 0.049 | 0.073 | -0.066 | 0.043 | -1.402 | -1.380 | -0.662 | -1.046 |
| ZNF860 | -0.056 | -0.062 | -0.141 | -0.033 | -1.176 | -1.005 | -0.459 | -0.761 |
| ZNRF1 | -0.040 | -0.005 | -0.052 | 0.149 | -0.518 | -1.288 | -0.648 | -1.312 |
| ZNRF2 | 0.033 | -0.162 | 0.001 | -0.199 | -0.554 | -0.142 | -0.445 | -0.180 |
| ZNRF3 | -0.101 | -0.023 | -0.164 | -0.197 | -0.523 | -0.508 | -0.203 | -0.143 |
| ZNRF4 | -0.169 | 0.020 | 0.347 | 0.212 | -0.310 | -0.369 | -2.493 | -1.899 |
| ZRANB1 | 0.059 | 0.183 | 0.259 | 0.177 | -1.258 | -2.362 | -1.964 | -2.214 |
| ZSWIM2 | -0.284 | -0.084 | -0.032 | -0.120 | -0.215 | -0.760 | -0.560 | -0.636 |

Supplementary table 1**:** A comprehensive list of the median LogFC score of the genes included in the library and the according RSA values represented as average LogP RSA score in high and low GFP expressing cells on day 7 and day 14 post-infection.


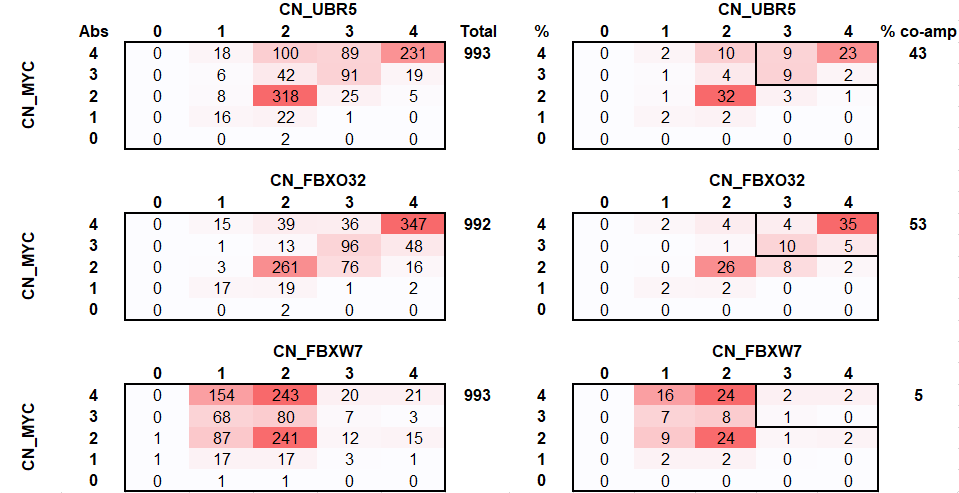
 Supplementary table 2**:** Correlation of cell line copy number (CN) estimates of UBR5, FBXO32, and FBXW7 show co-amplification of UBR5 and FBXO32 with MYC amplifications (see Fig. 5D). Represented are absolute values from > 990 cell lines analyzed (left, Abs) and relative values (right, %). % co-amplifications are calculated are represented in bins with CN>2.
